# *Ex Uno Plures*: Clonal Reinforcement Drives Evolution of a Simple Microbial Community

**DOI:** 10.1371/journal.pgen.1004430

**Published:** 2014-06-26

**Authors:** Margie Kinnersley, Jared Wenger, Evgueny Kroll, Julian Adams, Gavin Sherlock, Frank Rosenzweig

**Affiliations:** 1 Division of Biological Sciences, The University of Montana, Missoula, Montana, United States of America; 2 Department of Genetics, Stanford University School of Medicine, Stanford, California, United States of America; 3 Departments of Molecular, Cellular and Developmental Biology and Ecology and Evolutionary Biology, University of Michigan, Ann Arbor, Michigan, United States of America; University of Michigan, United States of America

## Abstract

A major goal of genetics is to define the relationship between phenotype and genotype, while a major goal of ecology is to identify the rules that govern community assembly. Achieving these goals by analyzing natural systems can be difficult, as selective pressures create dynamic fitness landscapes that vary in both space and time. Laboratory experimental evolution offers the benefit of controlling variables that shape fitness landscapes, helping to achieve both goals. We previously showed that a clonal population of *E. coli* experimentally evolved under continuous glucose limitation gives rise to a genetically diverse community consisting of one clone, CV103, that best scavenges but incompletely utilizes the limiting resource, and others, CV101 and CV116, that consume its overflow metabolites. Because this community can be disassembled and reassembled, and involves cooperative interactions that are stable over time, its genetic diversity is sustained by clonal reinforcement rather than by clonal interference. To understand the genetic factors that produce this outcome, and to illuminate the community's underlying physiology, we sequenced the genomes of ancestral and evolved clones. We identified ancestral mutations in intermediary metabolism that may have predisposed the evolution of metabolic interdependence. Phylogenetic reconstruction indicates that the lineages that gave rise to this community diverged early, as CV103 shares only one Single Nucleotide Polymorphism with the other evolved clones. Underlying CV103's phenotype we identified a set of mutations that likely enhance glucose scavenging and maintain redox balance, but may do so at the expense of carbon excreted in overflow metabolites. Because these overflow metabolites serve as growth substrates that are differentially accessible to the other community members, and because the scavenging lineage shares only one SNP with these other clones, we conclude that this lineage likely served as an “engine” generating diversity by creating new metabolic niches, but not the occupants themselves.

## Author Summary

The variability of natural systems makes it difficult to deduce how organisms' genotypes manifest as phenotypes, and how communities of interacting organisms arise. Using laboratory experimental evolution we can control this variation. We previously showed that a population of *E. coli* that originated from a single clone and was cultured in the presence of a single limiting resource, evolves into a stable, three-membered community, wherein one clone excretes metabolites that the others utilize as carbon sources. To discern the genetic factors at work in producing this outcome and to illuminate the community's physiology, we sequenced the genomes of the ancestral and evolved clones. We identified in the ancestor mutations that may have predisposed evolution of cross-feeding. We found that the lineages which gave rise to the community diverged early on, and that the numerically dominant lineage that best scavenges limiting glucose does so as a result of adaptive mutations that enhance glucose uptake but favor fermentative over respiratory pathways, resulting in overflow metabolites. Because this clone produces secondary resources that sustain other community members, and because it shares with them only one mutation, we conclude that it is an “engine” generating diversity by creating new niches, but not the occupants themselves.

## Introduction

*It is interesting to contemplate a tangled bank, clothed with many plants of many kinds, with birds singing on the bushes, with various insects flitting about, and with worms crawling through the damp earth, and to reflect that these elaborately constructed forms, so different from each other, and dependent upon each other in so complex a manner, have all been produced by laws acting around us…* Darwin, 1859

Illuminating the laws that produce Darwin's “tangled bank” remains one of the great challenges of biology, one that requires understanding how differences among forms are selected for, and how interdependence among forms is generated. In natural environments, meeting this challenge is complicated by the fact that selection pressures often vary widely over both space and time. Laboratory evolution experiments, in particular those using microbes, offer an attractive alternative by which to study, under controlled conditions, both the dynamic interplay between genotype and phenotype as well as the interactions among phenotypes in simple communities. Classical models of large asexually evolving populations led to the expectation that in simple environments complexity should be transient and limited in scope [1], [2]. Experimental evidence now suggests otherwise. Multiple genotypes that arise from a single ancestral clone can coexist over evolutionary time; in other words, *ex uno plures* (out of one many). This phenomenon has been documented in spatially and temporally unstructured chemostats [3], [4], in temporally-structured batch cultures [5]–[9] and in spatially-structured microcosms [10]. In each setting, the emergence and persistence of polymorphism in the absence of sexual recombination requires that cohabitants opportunistically exploit unoccupied niche space, and/or accept trade-offs between being a specialist and a generalist [11]–[13]. In serial dilution batch culture, multiple growth parameters can come under selection [14]: different clones may arise having reduced lag time, increased maximum specific growth rate, or enhanced capacity to survive at high cell densities in the presence of low nutrients [15]. Periodic changes in population density and nutrient levels may bring balancing selection to bear on these different phenotypes (e.g., [8]), especially if mutations are antagonistically pleiotropic [16], [17]. In spatially structured environments selection may favor mutants better adapted to particular regions or better able to colonize microhabitats formed at the boundaries between such regions. By contrast, in continuous nutrient-limited environments, theory predicts that selection will favor clones better able to scavenge the limiting resource or more efficiently convert that resource to progeny [18], [19]. Ultimately, the outcome of the ‘evolutionary play’ in any of these ‘ecological theaters’ (*sensu* [20]) will depend on founder genotype, mutation rate, the complexity of genetic pathways leading to different adaptive strategies, as well as pleiotropy [21] and epistatic interactions [22].

Increasing evidence points to the possibility of not one, but three potential outcomes when asexual microbes evolve in simple environments: *clonal succession*, where a population is successively swept by clones of higher fitness arising in the dominant lineage [23], [24]; *clonal interference*, where fixation of a single fittest clone is deferred because independent beneficial mutations arise in multiple, independent clones that compete with one another and reduce each other's fitness [25]–[27], and what we propose to call *clonal reinforcement*, where the emergence of one genotype favors the emergence and persistence of other genotypes via cooperative interactions. Because cooperation is now recognized to be at least as important as competition in driving biological innovation and in structuring communities [28], [29], we investigated the genetic and environmental factors that foster interdependence in an evolving lab population.

Previously [30], we examined the transcriptomes of an experimentally evolved *E. coli* community in which three different strains that arose from a common ancestor stably coexisted in a simple, unstructured environment through metabolic cross-feeding [3], [31]. This system is unusual in that it clearly involves symbiotic interactions among ecotypes that co-evolved in the absence of spatial or temporal variation. Remarkably, the community's expression profile did not proportionately represent the sum of each strain's expression profile grown singly under evolutionary conditions, indicating that the whole was not the sum of its parts. Instead, expression data suggested that consumption of one ecotype's overflow metabolites by the others relieved feedback inhibition, adding a layer of complexity to the evolved clones' interactions. Using a candidate gene approach we identified two mutations in the ancestral strain that may have predisposed clonal reinforcement [30]. To better understand how physiological performance and expression profile map onto genotype, and to identify the molecular basis of community interactions we sequenced the genomes of each of the three community members and their common ancestor. We found unexpectedly high levels of genetic variation in the three-membered community as a whole and the numerically dominant strain in particular, as well as a strong mutational bias due to specific lesions in DNA repair. The dominant community member is a hypermutator that excels at acquiring glucose, but does so at the expense of carbon excreted as overflow metabolites to which it has limited access; this trade-off effectively opens up new niches for other genotypes. We discovered a set of adaptive mutations in this clone that have not been previously reported to co-occur in other *E. coli* evolution experiments, specifically those that enhance glucose acquisition and may serve to maintain redox balance, but may also increase net flux to overflow metabolites under aerobic conditions. Because this community member releases growth substrates that are differentially accessible to other community members, and because it shares only one SNP mutation with these strains, we suggest that it acts as an engine generating biodiversity, creating new metabolic niches, but not necessarily the occupants themselves.

## Results and Discussion

### Description of the community

In previous publications [3], [30], [31] we described certain features of a stable polymorphism that arose from a single clone during the course of 765 generations of aerobic, glucose-limited culture at 30°C (D≈0.20 hr^−1^) (**Table 1**). The four clones isolated from this population (CV101, C103, CV115 and CV116) were originally distinguished by their differences in antibiotic resistance and colony morphology, and later shown to exhibit strain-specific rates of glucose uptake, residual metabolite concentrations and expression profiles. In reconstruction experiments performed under evolutionary conditions CV101, CV103 and CV116 were shown to stably coexist at frequencies of approximately 0.10, 0.65 and 0.025, respectively. Because CV115 was not stably maintained, the interactions among CV101, CV103 and CV116 were studied more intensively. Based on differences in glucose uptake and residual substrate concentrations, and the fact that strains' steady state frequencies could be predictably altered by exogenously increasing the concentrations of residual metabolites, we concluded that this community was sustained by positive density-dependent interactions in the form of cross-feeding, in which the numerically dominant strain CV103 best takes up the limiting resource glucose, but excretes acetate and glycerol (and/or a closely-related compound, glycerol 3-phosphate) (J. Adams unpublished results). These two overflow metabolites are then scavenged by CV101 and CV116, respectively. Transcriptional profiling of each clone in monoculture relative to the common ancestor, JA122, revealed gene expression differences among the evolved isolates related to carbon metabolism and expression differences specific to CV103 consistent with activation of stress response pathways and loss of motility.

**Table 1:** Strain characteristics.

Strain	Relevant Characteristics^2^	Mutation rate (/cell/gen)	Specific growth rate (hr^−1^)^2^	Relative growth yield^3^	Rate of glucose uptake (µmol αMG/min/gm)^3^	Equilibrium [glucose] (nmol/mL)^3^	Equilibrium [acetate] (nmol/mL)^3^
**K12 MG1655**	CGSC 6300F- rph-1 λ-	3.60×10^−9^					
**RH201** 1	CGSC 5346F- *thi 1leu6 thiI lacY1 tonA21 supE44 hss1 glpR200*						
**JA104**	Deriv. of RH 201 F- *thi 1 lacY1 araD139gdh supE44 hss1*; lysogenic for λ						
**JA122**	As JA104 but contains plasmid pBR322Δ5	1.00×10^−7^	0.44±0.01	1.14±0.02	1.19±0.09	1.84±0.48	194±20
**CV101**	Derivative of JA122; isolated after 773 generations, Amp^R^	1.33×10^−7^	0.50±0.02	1.11±0.02	1.66±0.06	0.88±0.31	0±0
**CV103**	As CV101 but independent isolate, forms small colonies on TA agar, Amp^R^	9.20×10^−7^	0.40±0.01	0.81±0.04	2.46±0.16	0.07±0.03	252±70
**CV115**	Derivative of JA122, isolated after 773 generations, lacks plasmid	1.14×10^−7^	0.55±0.02	1.11±0.02	ND	ND	ND
**CV116**	As CV115 but forms small colonies on TA agar	1.10×10^−7^	0.60±0.01	1.20±0.03	1.61±0.11	0.19±0.05	40±25

1 Ref. [149].

2 Data from [3], Table 1.

3 Data from [31], Table 2.

### Whole genome re-sequencing

As summarized in **Table 2**, 584 mutations (580 SNPs, two insertions and two deletions) were identified among the four evolved clones. This number is substantially higher than has been previously reported for laboratory evolution experiments of similar duration conducted under similar conditions [32]. A majority of these mutations, 504 (86%), is in coding regions and 374 (64%) are non-synonymous, while 428 (73%), are unique to CV103. Almost all (99.7% (578)) SNPs are GC→TA transversions, suggesting defects in DNA repair among all of the evolved isolates. Strong transversion bias has been noted in other evolution experiments, and is likely due to defective repair of oxidatively damaged G:C base-pairs [32], [33].

**Table 2:** Types of Single Nucleotide Polymorphisms (SNPs) and their distribution among evolved strains.

Total mutations	584
Insertions/deletions	4
SNPs	580
SNPs in coding regions	502 (86.6%)
SNPs causing missense mutations	335 (57.8%)
SNPs causing nonsense mutations	39 (6.7%)
G to A transitions	1 (0.2%)
C to T transitions	1 (0.2%)
**G to T transversions**	293 (50.5%)
**C to A transversions**	285 (49.1%)

### The community consists of lineages that diverged early in the history of the experiment

**Figure 1:**
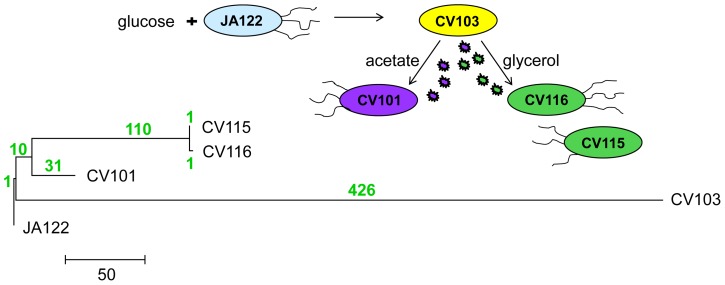
Phylogeny of strains based on whole-genome sequence. 580 SNPs across all four genomes were used to reconstruct evolutionary relationships between consortium members using maximum likelihood. The number of SNPs that distinguish each lineage are shown above each branch in green.

Phylogenetic analyses of the whole genome sequences [30] show that the dominant clone CV103 is part of a highly divergent lineage, while CV115 and CV116 are very closely related to one another, and share a common ancestor with CV101 (**Fig. 1**). Indeed, as CV115 and CV116 differ by only two SNPs, one a synonymous substitution (*gutQ*, A221A), the other C-terminal (*ycaO* G546V), and because only CV101, CV103 and CV116 could be stably maintained at steady state, we restrict most of our discussion to these three clones. Remarkably, the evolved clones all share only one SNP. Thus, it is likely that the CV103 and CV101/CV115/CV116 lineages diverged from one another early in the experiment and that stable co-existence of more than one lineage was an early feature of the population. In fact, previous work documented a variant similar to CV103 (small colony, ampicillin-resistant) had risen to appreciable frequency by generation 340 [3]. This result is not unexpected, as subsequent to the observations of Helling et al. 1987, a number of other reports appeared showing that multiple genotypes can coexist in nutrient-limited chemostats [34]–[38]. An unanticipated finding in our experiments was the large number of SNPs that accumulated along the CV103 branch.

### Mutation rate varies among evolved strains and their common ancestor

Fluctuation analyses performed on the ancestral strain, JA122, the canonical *E. coli* strain K12 MG1655, and the four co-evolved clones revealed that the founder strain, JA122, is a mutator whose mutation rate is approximately thirty-fold higher than *E. coli* K12 MG1655 (**Table 1**). This observation can be explained by a nonsense mutation in JA122 affecting the adenine glycosylase mismatch repair enzyme MutY (L299*). Defects in MutY are known to cause a GC→TA transversion bias [33], [39] and have been observed by others in glucose limited chemostat experiments [40], [41]. Evolved strains CV101, CV115 and CV116 all had mutation rates similar to JA122, while CV103's mutation rate was almost 10-fold higher again (**Table 1**). This difference can be attributed to a second mutation resulting in an amino acid substitution (A56D) in the base-excision repair pathway DNA glycosylase, MutM. Increased mutation rate has been shown to be selected for in microbial evolution experiments performed under a variety of conditions [33], [41]–[45] where it can strongly influence evolutionary dynamics [46]. The fitness of mutators need not decrease over time; Maharjan et al. [47] recently showed that mutators arising under glucose limitation gain fitness relative to their ancestor as a result of adaptive mutations in, for example, *rpoS* and *mglD*. Mutator lineages persisted because most of the other mutations accumulating in their backgrounds were neutral in their effect on fitness, deleterious mutations being purged as the lineages evolved. A similar mechanism may be at work in our experiment, with an appreciable number of mutations in CV103 being neutral.

### Certain transcriptional units are repeatedly mutated

Given the large number of SNPs, we sought to determine whether certain genes or transcription units carried multiple mutations, either because they had had been selected for or because their inactivation allowed neutral mutations to accumulate. Overall, 52 transcription units and 37 individual genes contained more than one SNP (**Table S1**). Twenty-five of the single/multiple gene hits occur exclusively in strain CV103 (**Table S1**). Four of these (*eno*, *maeA*, *malG* and *ptsI*) are directly involved in glucose uptake/metabolism suggesting that they contribute to the superior glucose uptake kinetics of this clone. Five CV103-specific substitutions affect flagellar synthesis: three SNPs occur in *fliM*, which encodes a flagellar motor switch protein, one occurs in *fliF*, which encodes the flagellar M ring protein, and one occurs in *fliH*, which encodes another flagellar biosynthesis protein. These findings are consistent with the previously observed down-regulation of flagellar genes in CV103 and the fact this strain is non-motile [30]. When all of the evolved isolates are considered, it is perhaps surprising that CV116, thought to salvage excreted glycerol/glycerol phosphate from CV103, has a number of mutations (10) in transcription units that are also mutated in CV103 (**Table S1**). The same is not true for CV101, the strain that salvages overflow acetate (**Table S1**). While most of these SNPs are silent mutations, they could plausibly affect RNA stability or translation. Of the non-silent changes, particularly noteworthy are four mutations (three in CV103 and one in CV116) that affect NADH:ubiquinone oxidoreductase I, as well as additional mutations affecting genes involved in anaerobic formate production and electron transfer (**Table S1**). Because both CV103 and CV116 show enhanced glucose uptake relative to CV101 and the ancestral strain (**Table 1** and [3]), these changes may be shared features of an adaptive response to rapid glucose consumption and concomitant overproduction of NADH (discussed below).

### Certain SNPs are associated with strain-specific differences in transcription and/or translation

Because we previously observed significant differences in gene expression among evolved isolates grown in monoculture [30], we sought to identify SNPs that could explain these differences. Because the great majority of these transcriptional differences were manifest in CV103, we focused on genes that could directly or indirectly regulate loci differentially expressed in this strain and on the 428 genes associated with a SNP in CV103. The intersection of these two gene lists yielded three global regulators that could explain the majority of transcriptional differences between CV103 and the other evolved isolates: *rpoD*, *csrA* and *sdiA*. RpoD, the housekeeping sigma factor, controls transcription of over 2,300 genes. The ancestor of the four evolved isolates, JA122, has an amber nonsense mutation (E26*, GAG→TAG) in *rpoD* that likely leads to reduced translation of RpoD, but not necessarily its complete absence, as JA122 also carries the supE44 amber suppressor. CV103 has an additional silent mutation in *rpoD* (T459T, ACC→ACA) that does not affect protein sequence but may affect translation, as the mutant codon ACA is 30% less common than the wild-type codon ACC [48]. The regulatory protein CsrA favors gluconeogenesis and glycogen synthesis over glycolysis. While the CV103 mutation in *csrA* is also silent (G27G, GGC→GGA), the resulting codon change is from one that is common (*f* = 0.40) to one that is rare (*f* = 0.09). Finally, *sdiA* encodes an N-acylhomoserine-L-lactone receptor that functions in quorum sensing. In CV103, a SNP is located 27 base-pairs upstream of the *sdiA* transcriptional start site in a region that encodes a small RNA, RNA0-361. The function of this sRNA is unknown, but it has been repeatedly identified in screens for small RNAs that interact with the global RNA chaperone, Hfq [49], [50], in which CV103 has a missense mutation (see below).

### Mutations that alter glucose transport: The outer membrane

**Figure 2:**
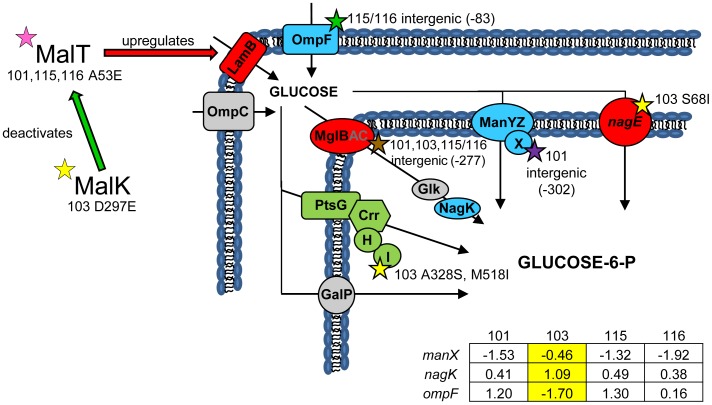
Gene expression and SNPs among loci that mediate glucose uptake. Several SNPs occur in or upstream of genes known or suspected to be involved in glucose uptake. Loci shown in green have lower monoculture transcription levels in all evolved isolates compared to the ancestor JA122, while loci shown in red have elevated monoculture transcript levels. Grey denotes no change in transcript level detected. Loci depicted in blue have different gene expression levels depending on the strain tested, and for these genes, the normalized log_2_ expression ratios of the evolved strain relative to JA122 are shown in the inset table (see Materials and Methods). Positive values indicate increased expression in the evolved isolates while negative values denote decreased expression. SNPs thought to affect proteins involved in glucose transport are indicated by stars with strain and mutation details as indicated. Consistent with strain representations in Figure 1 light blue stars indicate ancestral mutations present in JA122, purple indicates SNPs in CV101, yellow indicates SNPs in CV103, green indicates those in CV115/116, brown indicates SNPs shared by CV101/CV103/CV115/CV116 and pink denotes SNPs shared by CV101 and CV115/116.

(**Fig. 2**) In earlier work we showed that all evolved isolates were better able to scavenge limiting glucose than their common ancestor [31], and that strain CV103 had a significantly higher rate of glucose uptake than the other evolved isolates [3]. We later attributed these observations to increased expression of LamB glycoporin in all four strains relative to JA122 [51], and significantly higher transcription of *lamB* in CV103 relative to CV101, CV115 and CV116 [30]. In other studies of adaptation under glucose limitation elevated *lamB* expression has repeatedly been tied to mutations that affect the regulation of the Mal operon, specifically mutations that affect expression of the *lamB* regulators Mlc and/or MalT [35], [51], [52]. While we previously found that CV101, CV115 and CV116 share a mutation in *malT* that could up-regulate *lamB* expression, this mutation did not occur in CV103 [30], the strain that best scavenges limiting glucose.

Whole genome sequencing uncovered two other mutations in CV103 that help explain higher transcript levels of *lamB* mRNA and, by extension, its superior ability to scavenge limiting glucose. The first mutation affects the gene for MalK, a negative regulator of MalT [53]. Null mutations in *malK* promote constitutive *mal/lamB* gene expression [54], [55], presumably by disrupting the MalK-MalT interaction. The *malK* SNP in CV103 causes an amino acid change (D297E) in the C-terminal portion of the protein that contains an important part of the MalT interaction domain. Replacement of aa 297 is known to diminish the ability of MalK to inhibit MalT [56], and thus the D297E SNP likely leads to increased *lamB* transcription during glucose-limited growth [57]–[60].

The second CV103 mutation that may influence *lamB* expression occurs in the gene for the RNA chaperone Hfq. Hfq is a global regulator that facilitates binding of small regulatory RNAs (sRNAs) to mRNAs and by doing so affects translation and/or degradation of those transcripts [61], [62]. Details are scant as to how most of these sRNAs regulate their targets and how Hfq enhances or attenuates their activity, but the profound effect these interactions can have on key cellular processes is being increasingly recognized [61], [63]. Hfq interacts with the mRNA of the *E. coli* stationary phase sigma factor, RpoS, and is required for efficient translation of RpoS mRNA [64]. Reduced RpoS expression has been extensively studied as a key adaptation to continuous glucose-limitation [40], [65], [66], and Hfq has recently been identified as an alternative mutational target under glucose limitation in *rpoS* ^+^ strains [67], [68]. In these isolates, a missense mutation in Hfq enhances glucose uptake via PtsG, increases levels of LamB, and apparently reduces the amount of functional RpoS, resulting in lower biomass yield. The *hfq* mutation in CV103, which results in a Q52H substitution, could have similar effects: CV103 has increased uptake of the glucose analog α-MG (**Table 1**), which occurs exclusively via PtsG, increased LamB gene expression, and decreased biomass yield relative to the other evolved clones [3], [30]. Interestingly, the genomic context of the Hfq mutation in CV103 differs from those characterized by others in which defects in Hfq have evolved in an *rpoS* deficient background [67], [68]. CV103 and our other evolved isolates share an ancestral *rpoS*(Am) mutation which likely allows some RpoS translation.

### Mutations that alter glucose transport: The inner membrane

(**Fig. 2**) After entry into the periplasm, glucose is actively transported across the inner membrane using the glucose-specific sugar phosphotransferase system (PTS). Mutations that upregulate an alternative high-affinity glucose transporter, the galactose transporter MglBAC, are frequently observed following prolonged growth under glucose limitation [35], [51], [52]; in this regard, our system is no exception. In fact, the only SNP shared by all of the evolved isolates is in the operator sequence for the MglD repressor (*mglO*, C→A, +3 bp relative to the end of *mglD*), consistent with increased expression of the *mglBAC* transcription unit [30], [35].

**Figure 3:**
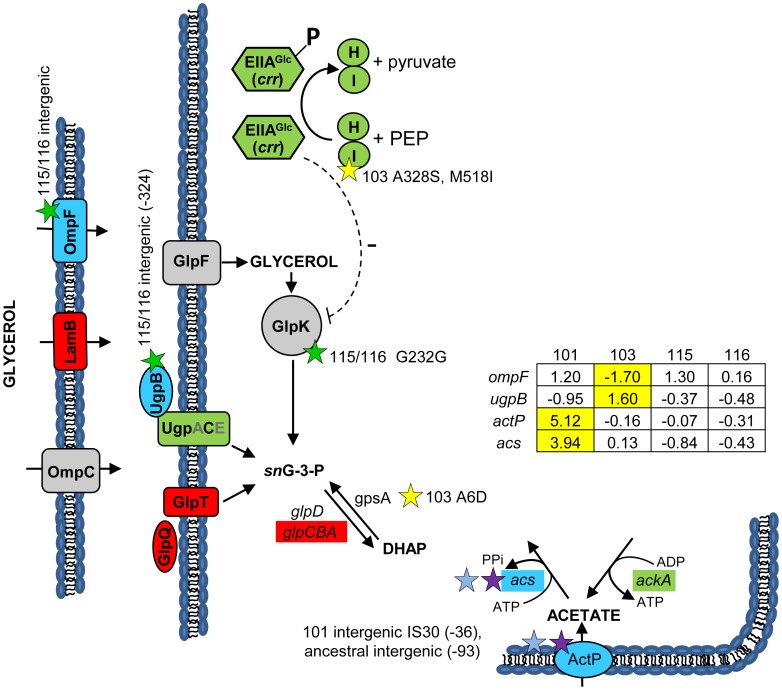
Gene expression and SNPs among loci that mediate glycerol and acetate uptake/metabolism. As in Figure 2, a green color indicates that the gene has a lower transcription level in monoculture chemostats relative to the ancestor JA122. A red color denotes elevated monoculture transcript levels while grey denotes no change in transcript level. Expression levels of loci that vary significantly in a strain-specific manner are shown in blue; for these genes, the normalized log_2_ expression ratios of the evolved strain relative to JA122 are shown in the inset table (for details see Materials and Methods). Positive values indicate increased expression in the evolved isolates while negative values denote decreased expression. Transcript ratios for these genes, relative to the ancestor JA122 grown under identical conditions, are presented in the inset table. Stars and corresponding text denote the location and type of particular SNPs. Consistent with strain representations in Figure 1, light blue stars indicate ancestral mutations present in JA122, purple indicates SNPs present in CV101, yellow indicates SNPs in CV103, green indicates those in CV115/116, brown indicates SNPs shared by CV101/CV103/CV115/CV116 and pink denotes SNPs shared by CV101 and CV115/116.

Because the rate of glucose uptake in CV103 exceeds that of the other clones, we looked for mutations that might affect expression or activity of other inner-membrane glucose transporters. CV103 has two mutations in *ptsI*, which encodes enzyme I of the *E. coli* sugar phosphotransferase system. This protein is active in its dimeric form and participates in glucose uptake by accepting a phosphate group from phophoenolpyruvate (PEP) and passing it via phosphocarrier protein Hpr to sugar-specific enzymes such as PtsG, which then use phosphate to “charge” incoming sugars (**Fig. 2** and **3**) [69], [70]. The mutations in *ptsI* result in two amino acid substitutions (A328S, M518I), both of which occur in the C-terminal region responsible for binding PEP and dimerization. These may be reasonably expected to alter PtsI activity [71], [72]; aa 328 is close to two residues that are part of the PEP binding site (R332 and D335), while aa 518 is close to C502, which is required for phosphotransfer [73], [74]. When ∼PO_4_ is not passed along by PtsI, there can be regulatory consequences: unphosphorylated PtsG can bind the transcriptional regulator Mlc and prevent it from negatively regulating expression of its targets which include *ptsG* itself, *manXYZ* and *malT* [75]–[77]. Thus, these mutations could also explain the up-regulation of *manXYZ* in CV103, and lead to higher expression of the LamB glycoporin though derepression of *malT* transcription [78]. PtsI has also been shown to interact directly with other phospho-enzymes, notably acetate kinase (*ackA*), thus mutations in this protein may also affect acetate excretion [79].

### Mutations in central metabolism may contribute to CV103's superior performance and propensity to create niches

**Figure 4:**
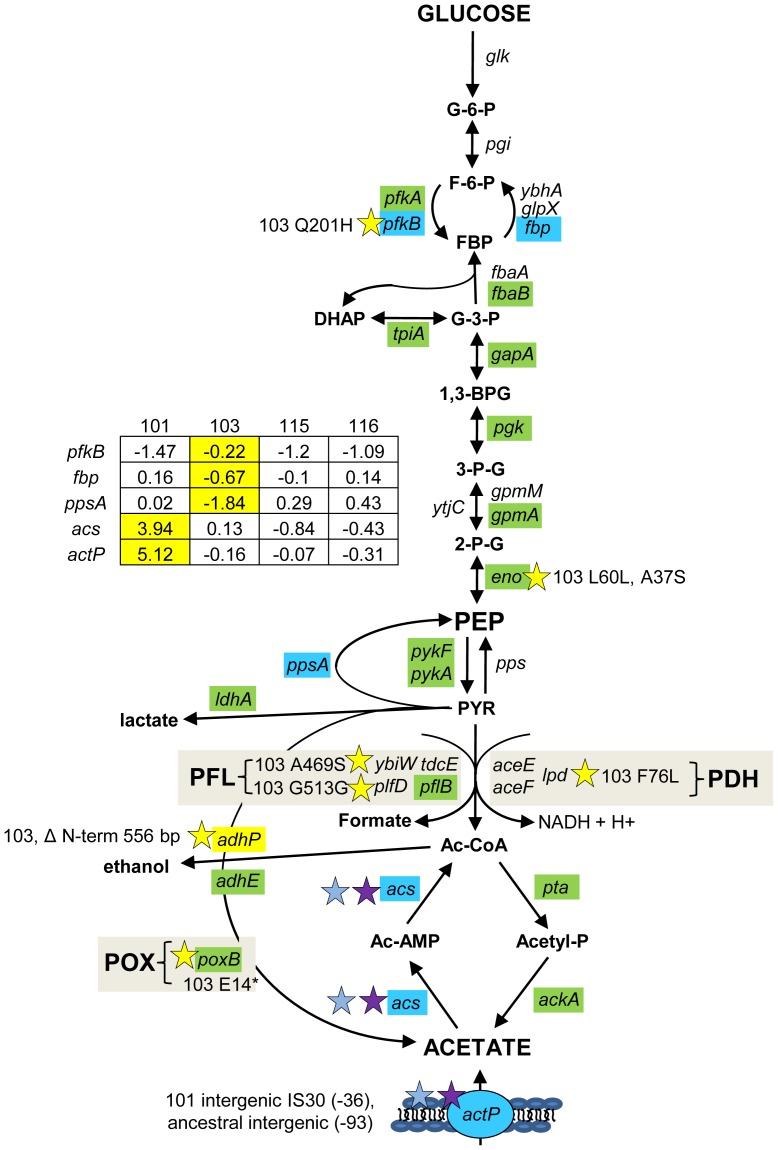
Gene expression and SNPs among loci that mediate glycolysis and fermentation. Mutations that may affect glycolysis and fermentation are restricted to the glucose-scavenger CV103. Green denotes lower transcription level in monoculture chemostats relative to the ancestor JA122. Red indicates higher transcript levels and grey denotes no transcript level change relative to JA122. Blue colored genes are those that show variable expression; normalized log_2_ expression ratios of the evolved strain relative to JA122 are shown in the inset table (for details see Materials and Methods). Positive values indicate increased expression in the evolved isolates while negative values denote decreased expression. Stars and corresponding text denote the location and type of particular SNPs. Consistent with strain representations in Figure 1, light blue stars indicate ancestral mutations present in JA122, purple indicates SNPs present in CV101, yellow indicates SNPs in CV103, green indicates those in CV115/116, brown indicates SNPs shared by CV101/CV103/CV115/CV116 and pink denotes SNPs shared by CV101 and CV115/116.

Although glucose transport is increased in all evolved isolates relative to their common ancestor, and especially so in CV103, expression of many glycolytic genes is decreased (**Fig. 4**). This trend has also been observed in yeast evolution experiments carried out under glucose limitation, and may reflect the selective advantage of an energy conservation strategy under low nutrient conditions [80]. Also, the hypermutator lineage that gave rise to CV103 accumulated a number of mutations predicted to impact conversions in glycolysis, fermentation, and the TCA cycle. Certain of these mutations likely underlie CV103's superior glucose uptake kinetics, but some may also favor excretion of and restricted access to overflow metabolites, which opens up new niches for other genotypes.

### Interconversion of fructose-6-phosphate and fructose-1,6-bisphosphate

In CV103, transcript levels of *pfkB*, which encodes PfkII, a secondary enzyme that converts fructose-6-phosphate into fructose-1,6,-bisphosphate, is more highly expressed in CV103 than in the other strains; CV103 also carries a missense mutation in this gene (Q201H) (**Fig. 4**). It is unclear whether this mutation is beneficial, as PfkII is thought to be responsible for less 5% of the phosphofructokinase activity in *E. coli* [81]. However, PfkII can also use tagatose-6-phosphate as a substrate [82]. CV103 has a surprising number of differences compared to the other clones in the expression and sequence of other genes in the galactitol/tagatose-6-P glycolytic pathway, including downregulation of *gatZY* (encoding tagatose-1,6-bisphosphate aldolase 2) and *gatABC* (galactitol PTS permease) as well as a mutation in the gene for GatY (G49*) (**Table S2**). As in a number of other K12-derived strains, the *gat* operon is likely constitutively expressed in JA122 due to IS3E element insertion in the galactitol regulator (GatR) gene [83]. Interestingly, increased expression of *gat* genes has been observed in experiments where *E. coli* has been evolved under lactulose and/or methyl-galactoside limitation [12], [84], [85].

Enolase, responsible for the conversion of 2-phosphoglycerate into phosphoenolpyruvate (PEP), has two mutations in CV103, one silent and one missense (L60L, A37S) (**Fig. 4** and **Table S2**). Aside from the regulatory role played by its product, PEP, enolase participates in the degradation of certain RNAs as part of the degradosome [86]. In particular, enolase is needed to degrade *ptsG* mRNA when intracellular levels of G-6-P are high (*i.e.*, during phosphosugar stress) [86], [87]. This interaction also involves the sRNA SgrS, Hfq and Pnp (polynucleotide phosphorylase), in which CV103 also has a substitution (P104Q) [50], [87]–[89] (**Table S2**). *ptsG* mRNA is not degraded when Hfq is mutated [90], thus in CV103 the combined action of mutations in enolase, Pnp and Hfq may increase longevity of *ptsG* transcripts.

### From pyruvate to acetyl CoA and/or acetate

Pyruvate is a major metabolite that sits at the branch point between glycolysis, the TCA cycle and fermentation (**Fig. 4**). Measurements of intracellular pyruvate indicated that all of the evolved isolates had significantly less intracellular pyruvate than their common ancestor under glucose-limited conditions, although no significant differences in pyruvate concentration could be detected among the evolved strains (**Table S3**).

**Figure 5:**
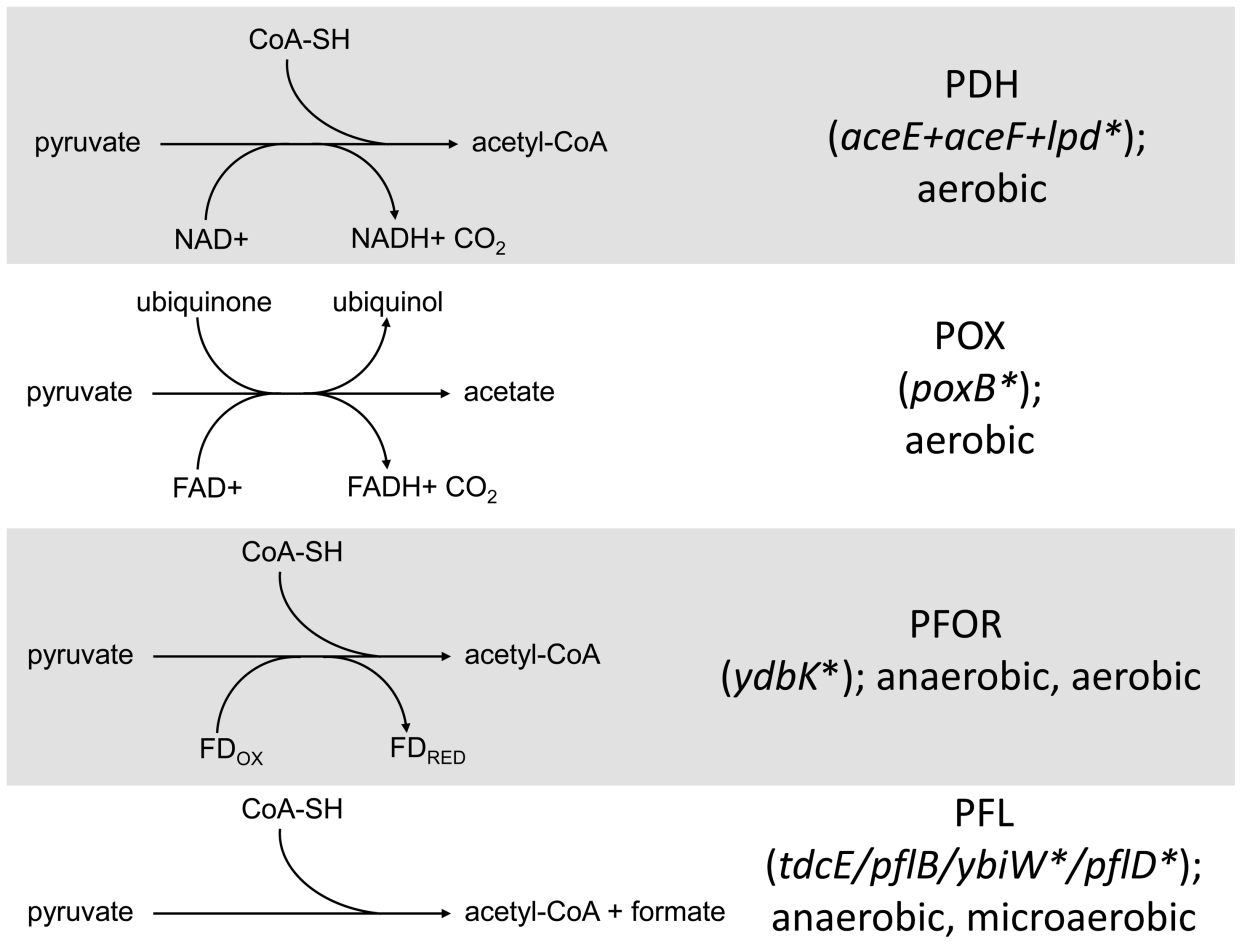
Distribution of SNPs in pathways that mediate pyruvate catabolism. Several SNPs were detected in genes involved in the conversion of pyruvate into acetate/acetyl Co-A in CV103. The four routes with their respective cofactors are shown on the left. Genes involved in the conversion are shown on the right with asterisks denoting those genes that have a SNP in CV103. Stars and corresponding text denote the location and type of particular SNPs. Consistent with strain representations in Figure 1 light blue stars indicate ancestral mutations present in JA122, purple indicates SNPs present in CV101, yellow indicates SNPs in CV103, green indicates those in CV115/116, brown indicates SNPs shared by CV101/CV103/CV115/CV116 and pink denotes SNPs shared by CV101 and CV115/116.

The primary route for oxidation of pyruvate during aerobic growth is pyruvate dehydrogenase (PDH), a three-enzyme complex that catalyzes the conversion of pyruvate into acetyl-CoA and contributes to the redox burden by transferring electrons to NAD+ (**Fig. 4** and **5**). Protein profiling of CV103 suggested the presence of a mutation in one of the three PDH enzymes, *lpd*, severe enough to eliminate the corresponding spot on a 2D gel [51]. Whole genome sequencing confirmed a missense mutation in the CV103 *lpd* gene that results in an amino acid substitution (F76L) in the N-terminal portion of the translated protein (**Table S2**). Because this substitution occurs in the FAD binding domain, it likely affects electron transfer from the reduced co-factor FADH_2_ to NAD+. PDH specific activity in CV103 was previously shown to be 2–3 fold lower than that in the other strains, indicating that this mutation does indeed have a negative effect [51]. Loss of *lpd* activity can lead to phenotypic changes consistent with many of the unique characteristics of CV103. In a screen for *E. coli* knockouts with extended lifespans, *lpd* null mutants were identified that had extended survival compared to wild-type *E. coli* K-12 MG1655 [91]. This enhanced survival was accompanied by reduced growth rate, reduced stationary phase cell density, reduced oxygen consumption, reduced respiration and increased accumulation of extracellular acetate, many of which phenotypes are exhibited by CV103 (**Table 1**, [3]).

Given that CV103 rapidly consumes glucose while excreting acetate, it seems unusual that a mutation affecting the conversion of pyruvate into acetyl-CoA would be retained in this strain. An *lpd* knockout can still grow on glucose and produce acetate, but it does so more slowly by using an alternate route to acetate, pyruvate oxidase (PoxB) [92]. In an effort to determine whether CV103 might be using an alternate enzymatic pathway to convert pyruvate into acetate, we compared gene expression and sequence data for the three alternative pyruvate oxidation pathways: pyruvate oxidase (POX), pyruvate:flavodoxin oxidoreductase (PFOR) and pyruvate formate-lyase (PFL) (**Fig. 5**).

The POX pathway directly converts pyruvate into acetate without concomitant ATP generation (**Fig. 5**). Nevertheless, this pathway is active during growth on glucose and can substitute for PDH if highly expressed [93]. In our system, expression of the gene for POX (*poxB*) is downregulated in all four evolved isolates relative to their common ancestor. This is not unexpected, as other glycolytic genes are downregulated. However, CV103 has a nonsense mutation at amino acid 14 of *poxB* (E14*) which, given its position at the extreme N-terminus, is likely to completely inactivate the protein. While the ancestral supE44 amber suppressor might allow limited *poxB* translation to occur, it is unlikely that the POX pathway produces an appreciable amount of acetate in CV103.

A second gene involved in acetate excretion, *ydbK* is expressed under both anaerobic and aerobic (albeit at very low levels) conditions (**Fig. 5**), [94], [95]. *ydbK* encodes PFOR, which catalyzes the conversion of pyruvate into acetyl-CoA with concomitant reduction of flavodoxin or ferredoxin [96]. Interestingly, both of these reduced molecules can be used to re-activate oxygen sensitive PFL (see below) [94]. CV103 carries a missense mutation of unknown effect (R539L) in *ydbK*; however, because this ORF was not represented on our expression array, its transcription level is unknown.

Finally, acetyl Co-A can also be produced by the cleavage of pyruvate by pyruvate formate-lyase (PFL). PFL activity is primarily associated with the *pflB* gene but is also encoded by *tdcE*, *ybiW* and *pflD* [97]–[99]. PFL is oxygen sensitive and thus the primary route to acetyl-CoA under anaerobic conditions, but it is transcribed, active and useful during microaerobiosis [100]–[103]. Moreover, production of PFL requires a smaller anabolic investment than production of PDH, and may thus be preferred under conditions of nutrient stress [104]. Functional PFL requires both transcriptional (regulated by FNR and ArcA/B) and post-translational activation by the activating enzyme PflA or the alternate activator YfiD [103], [105]. Consistent with the downregulation of many glycolytic genes, transcript levels of both the primary PFL (*pflB*) and its activator (*pflA*) are also downregulated across all of the evolved strains relative to the ancestor JA122. The gene for *pflB* is unchanged, and *pflA* has a silent substitution in CV103 (L205L). By contrast, transcript levels of the three alternate PFLs (*tdcE*, *ybiW*, *pflD*) and their alternate activator *yfiD* are not altered in any of the evolved strains relative to JA122, though CV103 has SNPs in the genes that encode two of the alternate PFLs: YbiW (A469S) and PflD (G513G) (**Fig. 5**).

Perturbations in pyruvate transformation are known to impact a key state variable in central metabolism: cellular redox balance. NADH accumulates in cells with high glycolytic flux because it cannot be re-oxidized as fast as it is generated. This increased redox ratio (NADH/NAD+) creates a cellular response reminiscent of anaerobiosis, stimulating the cell to direct pyruvate toward overflow metabolites and leading to repression of TCA cycle genes such as isocitrate dehydrogenase (*icd*) and citrate synthase (*gltA*) [106]. Given the relatively rapid uptake of glucose and higher rate of acetate production by CV103, stress in the form of high redox ratio may have influenced the evolution of this strain. NAD+ regeneration typically occurs downstream of pyruvate either via the TCA cycle or fermentation, but could also occur via the conversion of DHAP into glycerol (see section on glycerol metabolism below).

### Mutations in CV103 likely diminish flux through aerobic pathways

**Figure 6:**
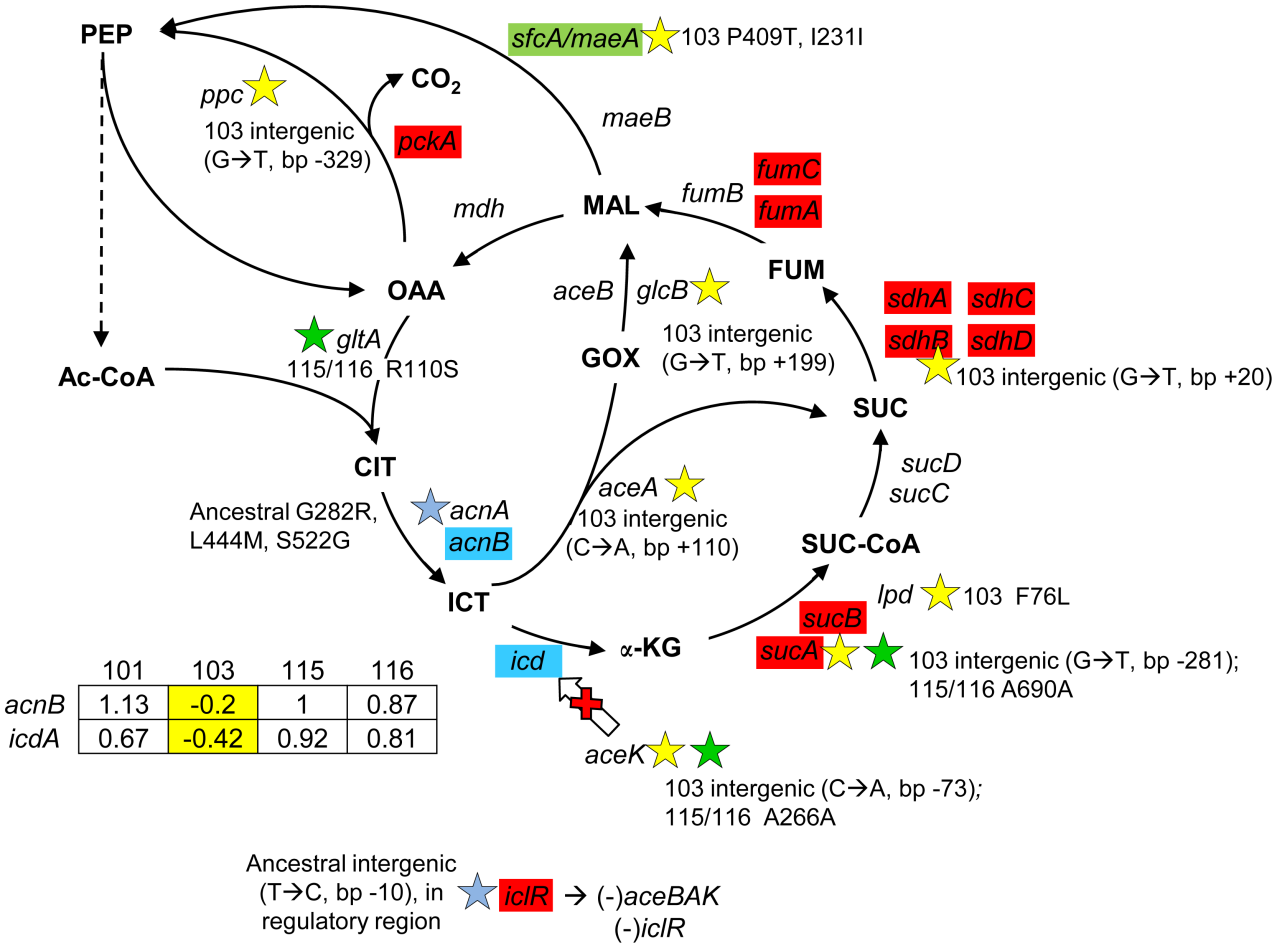
Gene expression and SNPs among loci in the TCA cycle and glyoxylate shunt. Loci that are part of the TCA cycle are associated with SNPs in CV103, CV115/CV116 and the ancestor JA122. Transcriptional profiling SAM analysis shows that many of the TCA cycle genes are up-regulated in the evolved strains relative to their comon ancestor, while two genes that control the TCA/glyoxylate switch point (*icd*, *acnB*) are expressed at a lower level in CV103. Blue colored genes are those that show variable expression among strains; normalized log_2_ expression ratios of the evolved strain relative to JA122 are shown in the inset table (for details see Materials and Methods). Color coding and symbols are the same as for Figures 2–5. Stars and corresponding text denote the location and type of particular SNPs. Consistent with strain representations in Figure 1 light blue stars indicate ancestral mutations present in JA122, purple indicates SNPs in CV101, yellow indicates SNPs in CV103, green indicates those in CV115/116, brown indicates SNPs shared by CV101/CV103/CV115/CV116 and pink denotes SNPs shared by CV101 and CV115/116.

(**Fig. 6**) The TCA cycle consists of eight steps beginning with the conversion of acetyl CoA to citrate and ending with the conversion of malate to oxaloacetate. Expression profiling of each evolved isolate showed that relative to their common ancestor, levels of transcripts for proteins involved in three of these steps (α-ketoglutarate dehydrogenase, succinate dehydrogenase and fumarase) were upregulated across all evolved strains. At two other steps, aconitase (*acnB*) and isocitrate dehydrogenase (*icdA*), transcript levels were elevated in three of four strains, but reduced in CV103. Whole-genome sequencing uncovered intergenic SNPs in CV103 that could impact flux at the *icd* branch point connecting the TCA cycle with the glyoxylate bypass. In the ancestral strain, JA122, the negative regulator of the glyoxylate bypass, *iclR*, has a promoter mutation that likely affects *iclR* negative autoregulation, leading to higher expression and concomitant repression of the glyoxylate shunt genes *aceA*, *aceB* and *aceK*. AceK negatively impacts flux through isocitrate dehydrogenase by phosphorylation, diverting carbon through isocitrate lyase (*aceA*) and malate synthase (*aceB*) (**Fig. 6**; [107]). Thus, in the ancestor increased IclR can reasonably be expected both to decrease transcription of glyoxylate bypass enzymes and to prevent inactivation of Icd by AceK. While CV101 and CV116 exhibit increased relative expression of *icdA* and *acnB*, CV103 shows decreased expression of both, perhaps owing to a C→A mutation that lies between the *aceA* and *aceK* open reading frames.

Cellular pyruvate levels have long been thought to modulate TCA cycle flux, principally by modulating isocitrate deydrogenase activity (**Fig. 6**, [108], [109]). The CV103-specific mutations described above may also affect the pool of pyruvate. *lpd* mutants have been shown to increase Entner-Doudoroff and glyoxylate shunt activities and to decrease TCA cycle activity [92], while *poxB* null mutations have been shown to repress citrate synthase and malate dehydrogenase, and to activate *acs* [110]. CV103 contains mutations in the *lpd* FAD binding domain, which likely accounts for the 2–3 fold reduction of its pyruvate dehydrogenase activity, as well as a nonsense mutation at amino acid 14 of *poxB* (E14*) that likely inactivates this protein (**Table S2**). Together these mutations could be expected to impede flux through the TCA cycle in CV103. Conspicuous among genes differentially expressed between evolved strains and their ancestor, and among the evolved strains themselves, were those encoding respiratory proteins that have a high H+/O coupling ratio. When each was grown by itself in glucose-limited chemostats, transcript levels for genes in the *cyoABCDE* operon that encode cytochrome oxidase subunits were significantly increased in CV101 and CV115/116, but significantly decreased in CV103, relative to their common ancestor JA122 [30].

Finally, CV103 also shows gene expression differences and mutations affecting the respiratory chain. Under conditions of nutrient stress, metabolic cost-benefit analyses predict *E. coli* will shift from the more efficient but anabolically expensive NADH:ubiquinone oxidoreductase I (*nuoABCEFGHIJKLMN*)/cytochrome bo oxidase (*cyoABCD*) chain to the anabolically inexpensive lower-yield NADH:ubiquinone oxidoreductase II (*ndh*)/cytochrome bd oxidase (*cydABX*) pairing [104], [111], [112]. Compared with JA122, expression of *nuoGHIJKL* is 1.1-fold lower in CV103 but 1.3 to 1.5-fold higher in CV101, CV116 and CV115. Similarly, transcript levels of *cyoABC* are 2.1-fold lower in CV103 and 1.3 to 1.9-fold higher in the other evolved strains. As noted, CV103 also has missense mutations that affect NuoM (L336F) and NuoI (R93L) as well as a silent substitution in NuoE (L14L). The effect of these differences in expression and sequence of NDH-I and cytochrome bo oxidase is unknown, but they may impact cellular redox balance.

In summary, by considering the whole genome sequencing data in the light of transcriptome [30], proteome [51], and phenotype data [31] a coherent picture emerges of the dominant clone, CV103, being highly fermentative but impaired in aerobic pathways, resulting in the production of overflow metabolites, which in the case of acetate creates a redox imbalance, but in the case of glycerol/glycerol phosphate, may provide a means to correct this imbalance. Because the other consortium members, like their common ancestor, are respiro-fermentative they are capable of exploiting the new biochemical niches created by CV103.

### The genetics of clonal reinforcement

(**Fig. 3**) We previously showed that differential production and scavenging of acetate, glycerol and/or glycerol phosphate explained stable coexistence of multiple genotypes under glucose limitation [31], [36]. Concerning acetate, whose excretion by CV103 supports growth of CV101, whole genome re-sequencing confirmed previously identified mutations in CV101 that up-regulate acetyl CoA-synthetase (Acs) and the acetate transporter ActP, as well as an ancestral mutation shared by the other clones that prevents efficient re-uptake of acetate by disrupting a CRP regulatory site in the *acs* promoter [30]. No additional mutations affecting acetate excretion or uptake were found in any of the evolved strains' genomes, so it is likely that these two alone explain the ability of CV101 to cross feed on the acetate produced by CV103.

Regarding glycerol and/or glycerol phosphate, previous experiments had shown that chemostat-grown CV116 assimilate radiolabeled glycerol 50% faster than all other strains, and that when CV116 and CV103 were co-cultured under glucose limitation, the addition of exogenous glycerol [31] or glycerol 3-phosphate caused the frequency of CV116 to increase. All of the evolved strains carry an ancestral mutation (G55A) in the DNA-binding transcriptional repressor, GlpR, that renders it constitutively inactive [113]. We also discovered a CV116-specific SNP in *glpK* encoding glycerol kinase, the first step in glycerol assimilation (**Table S2**), though this is a silent substitution at a site (G232G; GGC→GGA) not currently known to be associated with *glpK* regulation. Transcriptional profiling of GlpR regulated genes showed that the transcription unit containing glycerol kinase (*glpFKX*) remained at ancestral levels, while genes for the glycerol-3-phosphate:phosphate antiporter GlpT (*glpT*), and the divergently transcribed anaerobic glycerol-3-phosphate dehydrogenase genes (*glpABC*) were upregulated across all isolates relative to their common ancestor [30].

The absence of differences in *glp* gene expression between CV103 and CV116 led us to explore the possibility that glycerol and/or glycerol phosphate cross-feeding might be mediated by other mechanisms controlling the assimilation and production of these metabolites. For example, post-transcriptional regulation in CV103 could explain why JA122, CV101 and CV116 have three-fold higher specific activity of glycerol kinase, and two-fold higher specific activity of glycerol-3-phoshate dehydrogenase than CV103 [31]. GlpK is known to be post-transcriptionally inactivated by the unphosphorylated version of the glucose-specific PTS enzyme IIA^Glc^ (*crr*), or by an excess of the effector molecule fructose-1,6-bisphosphate [114]–[116]. As described above, IIA^Glc^ is phosphorylated via its interaction with the sugar non-specific EI (*ptsI*)/HPr (*ptsH*) (**Fig. 3**). When glucose is transported through the inner membrane by the Enzyme II^Glc^ complex (*ptsG*/*crr*), IIA^Glc^ (*crr*) transfers its phosphate to IIBC^glc^ (*ptsG*), which then phosphorylates glucose to yield intracellular glucose-6-phosphate (reviewed in [114]). High levels of unphosphorylated IIA^Glc^ signal glucose abundance, and inhibit enzymes needed for catabolism of alternate carbon sources such as glycerol and glycerol phosphate. Specifically, unphosphorylated IIA^Glc^ is known to inhibit glycerol kinase activity [114], [115], [117]. We have already noted two mutations in CV103 *ptsI*, which, by impairing EI, could lead to excess unphosphorylated IIA^Glc^, which would inhibit glycerol kinase (**Fig. 3**), and thereby restrict CV103's access to glycerol.

CV103 may also have an excess of another potent glycerol kinase inhibitor: fructose-1,6-bisphosphate (FBP) [116], [118]. We previously noted that relative to JA122 and the other evolved isolates, CV103 has enhanced expression of *pfkB*, which encodes the minor FBP creating enzyme, and lower levels of *fbp*, which encodes the reverse enzyme fructose bisphosphatase [30]. Whole-genome sequencing revealed a missense mutation of unknown effect in *pfkB* (Q201H). These distinctive features of CV103, combined with its demonstrated capacity for enhanced glucose transport and assimilation, may produce elevated levels of the GlpK inhibitor, FBP, further impeding CV103's ability to assimilate glycerol.

**Figure 7:**
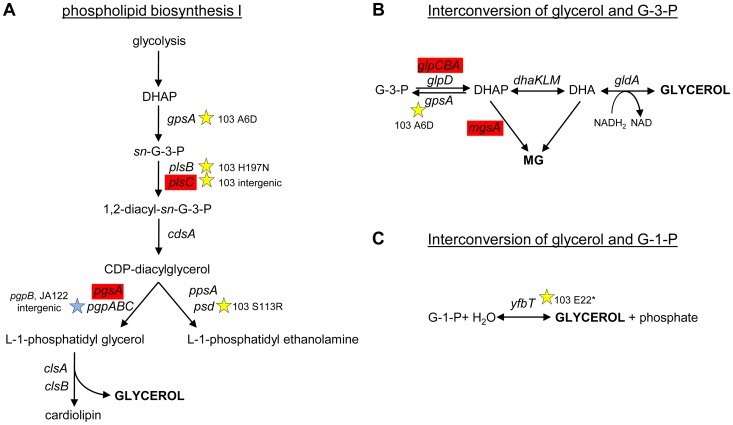
SNPs in genes involved phospholipid biosynthesis and that contribute to glycerol synthesis. Three possible routes to the production of glycerol in *E. coli* are shown along with corresponding gene names and SNPs. CV103 carries mutations that affect (A) phospholipid biosynthesis (B) the interconversion of glycerol-3-phosphate and glycerol and (C) the interconversion of glycerol-1-phosphate and glycerol. A red color denotes elevated monoculture transcript levels while grey denotes no change in transcript level. Stars (★) and corresponding text denote the location and type of particular SNPs. Consistent with strain representations in Figure 1 light blue stars indicate ancestral mutations present in JA122, purple indicates SNPs in CV101, yellow indicates SNPs in CV103, green indicates those in CV115/116, brown indicates SNPs shared by CV101/CV103/CV115/CV116 and pink denotes SNPs shared by CV101 and CV115/116.

To understand why CV103 might release glycerol and/or glycerol phosphate as metabolic by-products, we examined the transcript levels and sequences of genes encoding proteins involved in their production (**Fig. 3** and **Fig. 7**). Glycerol can be generated by *E. coli* as either (1) a by-product of phospholipid synthesis from sn-glycerol-3-phosphate, (2) an end-product of the detoxification of dihydroxyacetone phosphate/methylglyoxal, or (3) via hydrolysis of glycerol-1-phosphate [119]. CV103 has multiple mutations that affect nearly every step of phospholipid biosynthesis (**Fig. 7A**). While the number of SNPs suggests CV103 reaps some benefit from altering phospholipid production, these mutations should also have the effect of restricting glycerol and phospholipid formation from glycerol-3-phosphate. The second route to glycerol, detoxification of DHAP/methylglyoxal, is catalyzed by the reversible glycerol dehydrogenase GldA (**Fig. 7B**). This reaction is likely to be useful for a strain such as CV103 that is consuming glucose at a high rate, because it prevents the buildup of DHAP and subsequent production of toxic methylglyoxal and, significantly, because it re-oxidizes NADH [119], [120]. CV103 may also have higher amounts of intracellular DHAP and methylglyoxal as a consequence of a non-synonymous mutation (A6D) in the gene for glycerol-3-phosphate dehydrogenase GpsA, which favors the production of DHAP from G3P (**Fig. 3** and **7**). No SNPs affecting GldA were found, and *gldA* transcript levels were not up- or downregulated in any of the isolates. The third mechanism for the production of glycerol is via the activity of the enzyme YfbT. YfbT catalyzes the conversion of glycerol-1-phosphate into glycerol and its gene is truncated in CV103 (E22*), resulting in a defect likely expected to increase the pool of glycerol phosphate but not glycerol [121].

In short, there are multiple, non-mutually exclusive reasons to explain why CV103 has both a diminished capacity to assimilate glycerol as well as an increased propensity to produce glycerol and/or glycerol phosphate as overflow metabolites.

### Clonal reinforcement drives the evolution of biocomplexity

Biocomplexity, here defined as stable co-existence of multiple genotypes, can emerge in clonal populations evolving in environments that are spatially structured with respect to their physical features [10], [13] or temporally structured with respect to availability of limiting nutrients [8], [11], [122]. Under these conditions, the emergence of complexity can be explained in terms of classical niche theory [123]. Biocomplexity can also emerge in clonal populations evolving in simple, unstructured environments where reproduction is continuously limited by a single resource [3], [25], [47], [124]. There, population genetic complexity can be maintained by clonal interference, wherein competitive interactions preclude fixation of a fittest genotype [25], or by clonal reinforcement where one clone supports growth by others via the excretion of metabolizable substrates [3]. Our results bear out certain predictions arising from Mazancourt and Schwartz's resource ratio theory of cooperation [125]. In their model, two species initially competing for two resources can evolve towards a cooperative trading relationship one of whose emergent properties is enhanced resource utilization. Conditions that favor this outcome include low mortality, low resource levels and differential efficiency between species at depleting limiting resources. Our community evolved under similar conditions: clonal lineages persisted for many generations under chronic resource limitation and gained differential access to available resources. Two differences between their model and our system are that our population was founded by a single clone initially limited on one resource, and that the mutations which gave one of its descendants preferential access to that resource have pleiotropic effects that favor production of secondary resources on which other clones can thrive. As for when this genotype arose, we know by PCR analysis that key CV103-specific mutations at *malK*, *mutM*, *ptsI*, *hfq* and *lpd* are present in the earliest Helling et al. [3] population sample archived (∼350 generations) (data not shown).

### Clonal reinforcement is driven by the genotype best able to acquire the limiting resource

A category of mutations certain to prove beneficial under nutrient limitation is one that favors increased uptake of the limiting nutrient, in this case glucose. Levels of residual glucose in steady state CV103 monocultures are significantly lower than in those of the other evolved strains and their common ancestor [31], consistent with CV103's more rapid uptake of the non-metabolizable glucose analogue α-MG [3] and its higher expression of LamB glycoporin [51]. The amino acid substitution in MalK (D297E) likely diminishes its ability to deactivate MalT and, by extension, activates *lamB* expression. A mutation in Hfq (Q52H), that may negatively affect translation of the stress response global regulator RpoS, would enhance glucose scavenging via LamB and PtsG [126], [127].

### The evolution of a superior resource acquisition strategy results in trade-offs

Because glucose consumption in most organisms, including *E. coli*, is greater under anaerobic than under aerobic conditions (e.g., [128] and refs within), and because enhanced glucose consumption is an adaptive strategy selected for in glucose-limited chemostats, it is perhaps not surprising that, relative to the other evolved clones and their common ancestor, the metabolic profile of CV103 appears to be fermentative. Multiple lines of evidence support this interpretation: (i) TCA cycle genes encoding aconitase (*acnB*) and isocitrate dehydrogenase (*icd*) are downregulated (**Fig. 6**), (ii) expression of the *cyoABCD* operon encoding cytochrome oxidase is reduced [30], (iii) multiple mutations suggest CV103 is dealing with excess formate production (**Table S4**), and (iv) steady state chemostats of CV103 contain appreciable residual concentrations of overflow metabolites [3], [31]. While we found no CV103-specific mutations that would constitutively repress aerobic pathways, missense mutations at *lpd* and *maeA*, and a nonsense mutation at *poxB* could be expected to diminish TCA cycle flux. Moreover, increased glucose consumption by *E. coli* under glucose limitation has been shown to repress both respiration and the TCA cycle via changes in global regulators such redox balance and pyruvate [106]. High glucose consumption also strongly represses transcription of *acs* encoding acetyl CoA synthetase [106], which helps to explain why no activity of this acetate scavenging enzyme can be detected in CV103 monocultures [31], and why extracellular acetate is present at a dilution rate (D = 0.2 h^−1^) where none is expected. We therefore conclude that CV103's fermentative metabolism arises as a consequence of selection for enhanced glucose consumption.

The CV103 lineage's response to selection for enhanced glucose consumption results in trade-offs that critically determine its role in niche construction. At lower dilution rates *E. coli* growing under aerobic glucose-limitation usually carries out a high-yield metabolism that converts all available glucose to CO_2_ ([129] and refs therein). Indeed, under aerobic conditions the switch from respiratory to respiro-fermentative metabolism typically occurs at high growth rates and glucose concentrations, resulting in the production of overflow metabolites such as acetate. (Under anaerobic conditions, *E. coli* typically ferments all glucose to CO_2_, acetate and ethanol [130].) The excretion of overflow metabolites under conditions of restricted TCA flux creates an imbalance in cellular redox, because acetate production in *E. coli*, unlike ethanol production in yeast and lactate production in animals, fails to regenerate NAD+ from the NADH formed by glycolysis. In CV103 this problem may be further exacerbated by mutations affecting pyruvate transformation, which would place an additional premium on NAD+-generating processes, including reactions that lead to glycerol and glycerol-3-phosphate production. Concerning the apparent pleiotropic effects arising from constitutively high glucose consumption in the CV103 background, it interesting to note that a recently discovered mechanism to limit the production of overflow metabolites is to overexpress the small RNA SgrS, which effectively reduces the rate of glucose consumption [131]. SgrS, complexed with its binding partner, diminishes cells' ability to create new sugar transporters, in particular PtsG [132]. In CV103 the binding partner of SgrS, Hfq, has a missense mutation (Q52H), which may contribute to its rapid uptake, but incomplete assimilation of glucose.

The glucose scavenging adaptations seen in CV103, which contribute to its persistence and its ability to create new niches, contrasts with metabolic adaptations commonly observed in experimentally evolved yeast [21]. Yeast cultured at similar dilutions rates adapt to aerobic glucose limitation by reversing the Pasteur effect, switching from fermentative to respiratory metabolism, which results in an enormous gain in their reproductive output. While yeast evolution experiments typically begin with populations that produce appreciable amounts of overflow metabolites, mainly ethanol, strains quickly evolve a metabolic strategy that produces essentially no residual carbon [21], [133]. This difference in the adaptive trajectory followed by the two species is grounded in fundamental differences in their metabolism, which may preclude evolution of cross-feeding in yeast cultured under glucose limitation.

### The cost paid by one genotype to acquire the limiting resource opens up new niches for others

Superior glucose consumption by the largely fermentative strain CV103 helps to create multiple resources in an environment where initially only one was limiting, thereby providing a selective advantage to genotypes that can access those secondary resources. The mechanism by which strain CV101 gains preferential access to acetate is straightforward and relates to insertion of an IS*30* element in the upstream regulatory region of *acs* encoding the acetate-scavenging enzyme acetyl CoA synthetase [36]. The *acs* locus is misregulated in the ancestral background due to a mutation in the CRP binding site. Indeed, we observed appreciable amounts of acetate in JA122 monocultures at steady state [31], making it likely that selection pressure for acetate scavenging existed at the outset of these experiments. This pressure only increased with the advent of the CV103 lineage, which evolved a superior mechanism for glucose consumption, but which produces even more acetate because it retains the ancestral defect, and because increased flux through fermentative pathways represses *acs* transcription.

The basic CV103 phenotype is additionally supported by mutations, in particular those related to pyruvate oxidation (e.g. *lpd*), that accentuate changes in redox potential typical of fermentative cells whose overflow metabolite is acetate. These changes likely favor the formation of glycerol through side-reactions whose dehydrogenase steps help regenerate NAD+. CV116 has preferential access to this other metabolite because it retains two ancestral mutations: one in *acs* that impairs acetate scavenging, and another in *glpR* that constitutively derepresses enzymes required for glycerol and glycerol phosphate assimilation. By contrast, CV103 likely has limited access to these resources because it has accumulated mutations in *ptsI* and *pfkB*, whose downstream effects include inhibition of glycerol kinase, the rate-limiting step in glycerol assimilation.

### Reinforcement may underlie clonal diversity in the laboratory and in nature

The evolution and persistence of multiple genotypes in a population of asexual organisms supported by a single resource seems to violate the principles of competitive exclusion [134], [135] and periodic selection [1], [136]. Yet this phenomenon has now been observed in a variety of simple experimental systems, in particular those that are temporally [8], [16] or spatially structured [10], [13]. Although continuous nutrient-limited chemostats are unstructured in both respects, a number of studies have now shown that multiple bacterial lineages, some of them mutators, can arise and coexist in a single chemostat vessel [3], [35], [38], [47], [52], [137]. However, none of these studies have provided evidence that biocomplexity can arise and be sustained by means of the trading relationships we call clonal reinforcement. We contend that this mechanism may be quite common, as it is formally analogous to and may sometimes be a precursor to syntrophy, a ubiquitous feature of natural bacterial communities [138]. Clonal reinforcement may also be at work in clinically relevant environments. For example, the dominant clones in tumors are often fermentative, differing markedly from normal (ancestral) tissue in their demand for O_2_ and nutrients, their production of CO_2_ (see [139] and refs therein), and in their release of overflow metabolites that acidify the local environment [140]. Such cells may create opportunities for subpopulations to follow independent evolutionary trajectories that lead to further genetic differentiation, perhaps even to changes in their contact inhibition and drug resistance phenotypes. Chronic bacterial infections are also genetically heterogeneous [141], and can even be supported by syntrophic interactions [142]. We may therefore reasonably ask: to what extent does clonal reinforcement enable subpopulations in tumors and chronic infections to differentiate and become more resistant to chemotherapy and/or less visible to the immune system? The answers to these questions have far-reaching implications.

## Materials and Methods

### Strains, media and culture conditions

*Escherichia coli* JA122, CV101, CV103, CV115 and CV116 were maintained as permanent frozen stocks and stored at −80°C in 20% glycerol. Davis Minimal medium was used for all liquid cultures with 0.025% glucose added for batch cultures and 0.0125% for chemostats [143]. Chemostat cultures were initiated using colonies picked from Tryptone Agar (TA) plates and outgrown in Davis minimal batch medium overnight. For transcriptional profiling, total protein and metabolite assays chemostats were maintained at 30°C with a dilution rate of ≈0.2 h^−1^ for approximately 70 hours (∼15 generations). At the end of each chemostat run, three aliquots of 50 mL culture were rapidly filtered onto 0.2 µm nylon membranes, flash-frozen in liquid nitrogen and stored at −80°C.

### Mutation rate determination

Strains were streaked to colonies on LB agar overnight. Twelve colonies were picked in their entirety for each strain and inoculated into 3 mL of liquid LB, then grown overnight at 37°C. Subsequently, a portion of the liquid cultures was spread on LB plates containing 300 µg mL-1 rifampicin. Colonies were counted 48 h after spreading. To determine the titer, three cultures for each strain were spread on LB agar plates at 10^−7^, 10^−8^ and 10^−9^ dilutions. Colonies were counted and titers determined as the average of the three 10^−8^ dilutions. Mutation rates per cell per generation were calculated using maximum likelihood calculator FALCOR (http://www.keshavsingh.org/protocols/FALCOR.html) [144].

### Pyruvate assays

Following fast filtration and disruption by sonication intracellular pyruvate was determined on chemostat-grown cells using the Pyruvate Assay kit and PicoProbe (Biovision, Milpitas, CA, K609 and K317) as directed by the manufacturer's guidelines. Estimated values were normalized to cell protein, which was determined via the Pierce BCA Protein Assay Kit (Cat. # 23227, Thermo Scientific, Rockford, IL) using BSA as standard.

### Transcriptional profiling

Global gene profiling was described in detail in a previous publication [30]. Briefly, total RNA was extracted from triplicate cultures of chemostat-grown JA122, CV101, CV103 and CV116 (D = 0.2 h^−1^, 0.0125% glucose) and hybridized to full-length open reading frame PCR products spotted onto aminosilane-coated slides. Raw data was analyzed using TIGR MIDAS and MeV software pipelines (www.tm4.org) and Significance Analysis of Microarrays (SAM) [145] was used to examine expression differences between strains using a multi-class comparison consisting of four groups. Similarities among strains were identified using one-class SAM and differences between the strains were examined using a 4-class SAM. δ cutoffs were set at the 0% FDR threshold (i.e. the highest δ value that gave a median false discovery rate of 0%). Average (mean) log_2_ ratios were calculated after SAM analysis using Microsoft Excel and represent the relative expression ratios of each evolved isolate compared to their common ancestor.

### Nucleic acid extraction

Genomic DNA for Illumina sequencing was extracted from cells grown in batch culture using a modification of methods described by Syn and Swarup [146]. Subsequent to DNA precipitation, spun pellets were re-suspended in 1XTE (10 mM Tris, 1 mM EDTA, pH 8.0) containing 50 µg/mL DNAse-free RNAse A and incubated at 37°C for 30 minutes. Samples were re-extracted once with phenol:chloroform (3∶1), once with phenol:chloroform (1∶1) and twice with chloroform and then precipitated with EtOH using standard techniques. Following re-precipitation, DNA was dissolved in TE.

### Illumina sequencing

Single-end 36 bp sequencing libraries were created using the Illumina Genomic DNA Sample Prep Kit according to manufacturer's instructions (5 µg input genomic DNA), and sequencing flow cells were prepared using the Illumina Standard Cluster Generation Kit. Samples were sequenced on the Illumina Genome Analyzer II, and image analysis and data extraction were performed using Illumina RTA 1.5.35.0. Reads (with qualities) were aligned to the K12 reference genome (gi|49175990|ref|NC_000913.2) using BWA v0.5.8 [147] with default parameters. Whole-genome pileup files were generated using SAMtools v0.1.8–18 [148] and single-nucleotide polymorphisms due to the evolution were called using custom perl scripts that compared each evolved strain with the original ancestor. Briefly, SNPs passed the filter if they were represented in at least 40% of reads in the evolved strain and at most 10% in the reference strain, with at least 5 reads covering the position in both strains. Additional heuristic filters included a confirming read from both strands, and no more than one ambiguous SNP call (“N”) or deletion (“*”) at that position. Insertions and deletions relative to the K12 reference sequence were identified using Breseq v. 0.18 with default parameters (http://barricklab.org/breseq; [32]).

### Sanger sequencing

Thirty-seven SNPs identified by Illumina sequencing were verified by Sanger sequencing. PCR products were generated using Fermentas DreamTaq PCR Master Mix (Thermo Scientific, Waltham, MA) following manufacturer's instructions, treated with ExoSAP-IT (Affymetrix, Santa Clara, CA) and sequenced on an ABI3730XL machine by High Throughput Genomics Center (Seattle, WA) (**Table S5**).

## Supporting Information

Table S1 Genes and transcription units (T.U.) affected by more than one mutation.
(PDF)

Table S2 All mutations.
(PDF)

Table S3 Intracellular pyruvate.
(PDF)

Table S4 Mutations associated with formate metabolism.
(PDF)

Table S5 SNP verification primers.
(PDF)

## References

[1] Muller HJ (1932) Some genetic aspects of sex. Am Naturalist 66: 118–138.

[2] Williams GC (1975) Sex and Evolution. Princeton, NJ: Princeton University Press.

[3] Helling RB, Vargas CN, Adams J (1987) Evolution of Escherichia coli during growth in a constant environment. Genetics 116: 349–358. 3301527 10.1093/genetics/116.3.349 PMC1203146

[4] Ferenci T (2008) Bacterial physiology, regulation and mutational adaptation in a chemostat environment. Adv Microb Physiol 53: 169–229. 17707145 10.1016/S0065-2911(07)53003-1

[5] Lang GI, Rice DP, Hickman MJ, Sodergren E, Weinstock GM, et al (2013) Pervasive genetic hitchhiking and clonal interference in forty evolving yeast populations. Nature 500: 571–574. 23873039 10.1038/nature12344 PMC3758440

[6] Le Gac M, Brazas MD, Bertrand M, Tyerman JG, Spencer CC, et al (2008) Metabolic changes associated with adaptive diversification in Escherichia coli. Genetics 178: 1049–1060. 18245349 10.1534/genetics.107.082040 PMC2248342

[7] Spencer CC, Bertrand M, Travisano M, Doebeli M (2007) Adaptive diversification in genes that regulate resource use in Escherichia coli. PLoS Genet 3: e15. 17238290 10.1371/journal.pgen.0030015 PMC1779306

[8] Rozen DE, Lenski RE (2000) Long-Term Experimental Evolution in Escherichia coli. VIII. Dynamics of a Balanced Polymorphism. Am Nat 155: 24–35. 10657174 10.1086/303299

[9] Turner PE, Souza V, Lenski RE (1996) Tests of Ecological Mechanisms Promoting the Stable Coexistence of Two Bacterial Genotypes. Ecology 77: 2119–2129.

[10] Rainey PB, Travisano M (1998) Adaptive radiation in a heterogeneous environment. Nature 394: 69–72. 9665128 10.1038/27900

[11] Herron MD, Doebeli M (2013) Parallel evolutionary dynamics of adaptive diversification in Escherichia coli. PLoS Biol 11: e1001490. 23431270 10.1371/journal.pbio.1001490 PMC3576414

[12] Zhong S, Khodursky A, Dykhuizen DE, Dean AM (2004) Evolutionary genomics of ecological specialization. Proc Natl Acad Sci U S A 101: 11719–11724. 15289609 10.1073/pnas.0404397101 PMC511043

[13] Rainey PB, Buckling A, Kassen R, Travisano M (2000) The emergence and maintenance of diversity: insights from experimental bacterial populations. Trends Ecol Evol 15: 243–247. 10802550 10.1016/s0169-5347(00)01871-1

[14] Rosenzweig F, Sherlock G (2011) Through a Glass, Clearly: Experimental Evolution as a Window on Adaptive Genome Evolution. In: Garland JT, Rose MR, editors. EXPERIMENTAL EVOLUTION: Concepts, Methods, and Applications of Selection Experiments: University of California Press.

[15] Novak M, Pfeiffer T, Lenski RE, Sauer U, Bonhoeffer S (2006) Experimental tests for an evolutionary trade-off between growth rate and yield in E. coli. Am Nat 168: 242–251. 16874633 10.1086/506527

[16] Le Gac M, Plucain J, Hindre T, Lenski RE, Schneider D (2012) Ecological and evolutionary dynamics of coexisting lineages during a long-term experiment with Escherichia coli. Proc Natl Acad Sci U S A 109: 9487–9492. 22645336 10.1073/pnas.1207091109 PMC3386082

[17] Rozen DE, Philippe N, Arjan de Visser J, Lenski RE, Schneider D (2009) Death and cannibalism in a seasonal environment facilitate bacterial coexistence. Ecol Lett 12: 34–44. 19019196 10.1111/j.1461-0248.2008.01257.x

[18] Monod J (1942) Recherche sur la croissance des cultures bacteriennes. Paris: Hermann et Cie.

[19] Kubitschek HE (1970) Introduction to Research with Continuous Cultures. Englewood Cliffs, N.J: Prentice-Hall.

[20] Hutchinson GE (1965) The Ecological Theater and the Evolutionary Play: Yale University Press.

[21] Wenger JW, Piotrowski J, Nagarajan S, Chiotti K, Sherlock G, et al (2011) Hunger artists: yeast adapted to carbon limitation show trade-offs under carbon sufficiency. PLoS Genet 7: e1002202. 21829391 10.1371/journal.pgen.1002202 PMC3150441

[22] Kvitek DJ, Sherlock G (2011) Reciprocal sign epistasis between frequently experimentally evolved adaptive mutations causes a rugged fitness landscape. PLoS Genet 7: e1002056. 21552329 10.1371/journal.pgen.1002056 PMC3084205

[23] Novick A, Szilard L (1950) Experiments with the Chemostat on spontaneous mutations of bacteria. Proc Natl Acad Sci U S A 36: 708–719. 14808160 10.1073/pnas.36.12.708 PMC1063276

[24] Atwood KC, Schneider LK, Ryan FJ (1951) Periodic Selection in Escherichia coli. Proc Natl Acad Sci U S A 37: 146–155. 14808170 10.1073/pnas.37.3.146 PMC1063322

[25] Kao KC, Sherlock G (2008) Molecular characterization of clonal interference during adaptive evolution in asexual populations of Saccharomyces cerevisiae. Nat Genet 40: 1499–1504. 19029899 10.1038/ng.280 PMC2596280

[26] de Visser JA, Rozen DE (2006) Clonal interference and the periodic selection of new beneficial mutations in Escherichia coli. Genetics 172: 2093–2100. 16489229 10.1534/genetics.105.052373 PMC1456385

[27] Gerrish PJ, Lenski RE (1998) The fate of competing beneficial mutations in an asexual population. Genetica 102–103: 127–144. 9720276

[28] Boucher DH, ed. (1985) The Biology of Mutualism. NY: Oxford University Press.

[29] Thompson JN (2005) The geographic mosaic of coevolution. Chicago, Illinois: University of Chicago Press.

[30] Kinnersley MA, Holben WE, Rosenzweig F (2009) E Unibus Plurum: genomic analysis of an experimentally evolved polymorphism in Escherichia coli. PLoS Genet 5: e1000713. 19893610 10.1371/journal.pgen.1000713 PMC2763269

[31] Rosenzweig RF, Sharp RR, Treves DS, Adams J (1994) Microbial evolution in a simple unstructured environment: genetic differentiation in Escherichia coli. Genetics 137: 903–917. 7982572 10.1093/genetics/137.4.903 PMC1206068

[32] Barrick JE, Yu DS, Yoon SH, Jeong H, Oh TK, et al (2009) Genome evolution and adaptation in a long-term experiment with Escherichia coli. Nature 461: 1243–1247. 19838166 10.1038/nature08480

[33] Nghiem Y, Cabrera M, Cupples CG, Miller JH (1988) The mutY gene: a mutator locus in Escherichia coli that generates G.C----T.A transversions. Proc Natl Acad Sci U S A 85: 2709–2713. 3128795 10.1073/pnas.85.8.2709 PMC280068

[34] Ferenci T (2003) What is driving the acquisition of mutS and rpoS polymorphisms in Escherichia coli? Trends Microbiol 11: 457–461. 14557028 10.1016/j.tim.2003.08.003

[35] Notley-McRobb L, Ferenci T (1999) The generation of multiple co-existing mal-regulatory mutations through polygenic evolution in glucose-limited populations of Escherichia coli. Environ Microbiol 1: 45–52. 11207717 10.1046/j.1462-2920.1999.00003.x

[36] Treves DS, Manning S, Adams J (1998) Repeated evolution of an acetate-crossfeeding polymorphism in long-term populations of Escherichia coli. Mol Biol Evol 15: 789–797. 9656481 10.1093/oxfordjournals.molbev.a025984

[37] Maharjan RP, Gaffe J, Plucain J, Schliep M, Wang L, et al (2013) A case of adaptation through a mutation in a tandem duplication during experimental evolution in Escherichia coli. BMC Genomics 14: 441. 23822838 10.1186/1471-2164-14-441 PMC3708739

[38] Maharjan R, Seeto S, Notley-McRobb L, Ferenci T (2006) Clonal adaptive radiation in a constant environment. Science 313: 514–517. 16825532 10.1126/science.1129865

[39] Lin JC, Singh RR, Cox DL (2008) Theoretical study of DNA damage recognition via electron transfer from the [4Fe-4S] complex of MutY. Biophysical journal 95: 3259–3268. 18599627 10.1529/biophysj.108.132183 PMC2547449

[40] Notley-McRobb L, King T, Ferenci T (2002) rpoS mutations and loss of general stress resistance in Escherichia coli populations as a consequence of conflict between competing stress responses. J Bacteriol 184: 806–811. 11790751 10.1128/JB.184.3.806-811.2002 PMC139526

[41] Notley-McRobb L, Pinto R, Seeto S, Ferenci T (2002) Regulation of mutY and nature of mutator mutations in Escherichia coli populations under nutrient limitation. J Bacteriol 184: 739–745. 11790743 10.1128/JB.184.3.739-745.2002 PMC139514

[42] Cox EC (1976) Bacterial mutator genes and the control of spontaneous mutation. Annu Rev Genet 10: 135–156. 797306 10.1146/annurev.ge.10.120176.001031

[43] Cox EC, Gibson TC (1974) Selection for high mutation rates in chemostats. Genetics 77: 169–184. 4603159 10.1093/genetics/77.2.169 PMC1213122

[44] Desai MM, Fisher DS (2011) The balance between mutators and nonmutators in asexual populations. Genetics 188: 997–1014. 21652523 10.1534/genetics.111.128116 PMC3176104

[45] Gibson TC, Scheppe ML, Cox EC (1970) Fitness of an Escherichia coli mutator gene. Science 169: 686–688. 4914168 10.1126/science.169.3946.686

[46] de Visser JA (2002) The fate of microbial mutators. Microbiology 148: 1247–1252. 11988499 10.1099/00221287-148-5-1247

[47] Maharjan RP, Liu B, Li Y, Reeves PR, Wang L, et al (2013) Mutation accumulation and fitness in mutator subpopulations of Escherichia coli. Biol Lett 9: 20120961. 23221876 10.1098/rsbl.2012.0961 PMC3565518

[48] Maloy SR, Stewart VJ, Taylor RK (1996) Genetic analysis of pathogenic bacteria: A laboratory manual: Cold Spring Harbor Laboratory Press.

[49] Raghavan R, Groisman EA, Ochman H (2011) Genome-wide detection of novel regulatory RNAs in E. coli. Genome Res 21: 1487–1497. 21665928 10.1101/gr.119370.110 PMC3166833

[50] Zhang A, Wassarman KM, Rosenow C, Tjaden BC, Storz G, et al (2003) Global analysis of small RNA and mRNA targets of Hfq. Mol Microbiol 50: 1111–1124. 14622403 10.1046/j.1365-2958.2003.03734.x

[51] Kurlandzka A, Rosenzweig RF, Adams J (1991) Identification of adaptive changes in an evolving population of Escherichia coli: the role of changes with regulatory and highly pleiotropic effects. Mol Biol Evol 8: 261–281. 2072859 10.1093/oxfordjournals.molbev.a040650

[52] Notley-McRobb L, Ferenci T (1999) Adaptive mgl-regulatory mutations and genetic diversity evolving in glucose-limited Escherichia coli populations. Environ Microbiol 1: 33–43. 11207716 10.1046/j.1462-2920.1999.00002.x

[53] Joly N, Bohm A, Boos W, Richet E (2004) MalK, the ATP-binding cassette component of the Escherichia coli maltodextrin transporter, inhibits the transcriptional activator malt by antagonizing inducer binding. J Biol Chem 279: 33123–33130. 15180985 10.1074/jbc.M403615200

[54] Kuhnau S, Reyes M, Sievertsen A, Shuman HA, Boos W (1991) The activities of the Escherichia coli MalK protein in maltose transport, regulation, and inducer exclusion can be separated by mutations. J Bacteriol 173: 2180–2186. 2007546 10.1128/jb.173.7.2180-2186.1991 PMC207765

[55] Bukau B, Ehrmann M, Boos W (1986) Osmoregulation of the maltose regulon in Escherichia coli. J Bacteriol 166: 884–891. 2423504 10.1128/jb.166.3.884-891.1986 PMC215209

[56] Bohm A, Diez J, Diederichs K, Welte W, Boos W (2002) Structural model of MalK, the ABC subunit of the maltose transporter of Escherichia coli: implications for mal gene regulation, inducer exclusion, and subunit assembly. J Biol Chem 277: 3708–3717. 11709552 10.1074/jbc.M107905200

[57] Notley L, Ferenci T (1995) Differential expression of mal genes under cAMP and endogenous inducer control in nutrient-stressed Escherichia coli. Mol Microbiol 16: 121–129. 7651130 10.1111/j.1365-2958.1995.tb02397.x

[58] Decker K, Peist R, Reidl J, Kossmann M, Brand B, et al (1993) Maltose and maltotriose can be formed endogenously in Escherichia coli from glucose and glucose-1-phosphate independently of enzymes of the maltose system. J Bacteriol 175: 5655–5665. 8366051 10.1128/jb.175.17.5655-5665.1993 PMC206624

[59] Raibaud O, Vidal-Ingigliardi D, Richet E (1989) A complex nucleoprotein structure involved in activation of transcription of two divergent Escherichia coli promoters. J Mol Biol 205: 471–485. 2538630 10.1016/0022-2836(89)90218-0

[60] Raibaud O, Richet E (1987) Maltotriose is the inducer of the maltose regulon of Escherichia coli. J Bacteriol 169: 3059–3061. 3298211 10.1128/jb.169.7.3059-3061.1987 PMC212348

[61] Vogel J, Luisi BF (2011) Hfq and its constellation of RNA. Nat Rev Microbiol 9: 578–589. 21760622 10.1038/nrmicro2615 PMC4615618

[62] Valentin-Hansen P, Eriksen M, Udesen C (2004) The bacterial Sm-like protein Hfq: a key player in RNA transactions. Mol Microbiol 51: 1525–1533. 15009882 10.1111/j.1365-2958.2003.03935.x

[63] Sobrero P, Valverde C (2012) The bacterial protein Hfq: much more than a mere RNA-binding factor. Crit Rev Microbiol 38: 276–299. 22435753 10.3109/1040841X.2012.664540

[64] Muffler A, Fischer D, Hengge-Aronis R (1996) The RNA-binding protein HF-I, known as a host factor for phage Qbeta RNA replication, is essential for rpoS translation in Escherichia coli. Genes Dev 10: 1143–1151. 8654929 10.1101/gad.10.9.1143

[65] Seeto S, Notley-McRobb L, Ferenci T (2004) The multifactorial influences of RpoS, Mlc and cAMP on ptsG expression under glucose-limited and anaerobic conditions. Res Microbiol 155: 211–215. 15059634 10.1016/j.resmic.2003.11.011

[66] Notley-McRobb L, Seeto S, Ferenci T (2003) The influence of cellular physiology on the initiation of mutational pathways in Escherichia coli populations. Proc Biol Sci 270: 843–848. 12737663 10.1098/rspb.2002.2295 PMC1691312

[67] Maharjan R, Zhou Z, Ren Y, Li Y, Gaffe J, et al (2010) Genomic identification of a novel mutation in hfq that provides multiple benefits in evolving glucose-limited populations of Escherichia coli. J Bacteriol 192: 4517–4521. 20543067 10.1128/JB.00368-10 PMC2937378

[68] Maharjan RP, Ferenci T (2013) Epistatic interactions determine the mutational pathways and coexistence of lineages in clonal Escherichia coli populations. Evolution 67: 2762–2768. 24033182 10.1111/evo.12137

[69] Patel HV, Vyas KA, Mattoo RL, Southworth M, Perler FB, et al (2006) Properties of the C-terminal domain of enzyme I of the Escherichia coli phosphotransferase system. J Biol Chem 281: 17579–17587. 16547354 10.1074/jbc.M508966200

[70] Postma PW, Lengeler JW, Jacobson GR (1993) Phosphoenolpyruvate:carbohydrate phosphotransferase systems of bacteria. Microbiol Rev 57: 543–594. 8246840 10.1128/mr.57.3.543-594.1993 PMC372926

[71] Chauvin F, Fomenkov A, Johnson CR, Roseman S (1996) The N-terminal domain of Escherichia coli enzyme I of the phosphoenolpyruvate/glycose phosphotransferase system: molecular cloning and characterization. Proc Natl Acad Sci U S A 93: 7028–7031. 8692938 10.1073/pnas.93.14.7028 PMC38929

[72] Seok YJ, Lee BR, Zhu PP, Peterkofsky A (1996) Importance of the carboxyl-terminal domain of enzyme I of the Escherichia coli phosphoenolpyruvate: sugar phosphotransferase system for phosphoryl donor specificity. Proc Natl Acad Sci U S A 93: 347–351. 8552636 10.1073/pnas.93.1.347 PMC40235

[73] Teplyakov A, Lim K, Zhu PP, Kapadia G, Chen CC, et al (2006) Structure of phosphorylated enzyme I, the phosphoenolpyruvate:sugar phosphotransferase system sugar translocation signal protein. Proc Natl Acad Sci U S A 103: 16218–16223. 17053069 10.1073/pnas.0607587103 PMC1618308

[74] Garcia-Alles LF, Alfonso I, Erni B (2003) Enzyme I of the phosphotransferase system: induced-fit protonation of the reaction transition state by Cys-502. Biochemistry 42: 4744–4750. 12705838 10.1021/bi034007f

[75] Nam TW, Cho SH, Shin D, Kim JH, Jeong JY, et al (2001) The Escherichia coli glucose transporter enzyme IICB(Glc) recruits the global repressor Mlc. Embo J 20: 491–498. 11157755 10.1093/emboj/20.3.491 PMC133465

[76] Lee SJ, Boos W, Bouche JP, Plumbridge J (2000) Signal transduction between a membrane-bound transporter, PtsG, and a soluble transcription factor, Mlc, of Escherichia coli. Embo J 19: 5353–5361. 11032803 10.1093/emboj/19.20.5353 PMC313994

[77] Zeppenfeld T, Larisch C, Lengeler JW, Jahreis K (2000) Glucose transporter mutants of Escherichia coli K-12 with changes in substrate recognition of IICB(Glc) and induction behavior of the ptsG gene. J Bacteriol 182: 4443–4452. 10913077 10.1128/jb.182.16.4443-4452.2000 PMC94615

[78] Decker K, Plumbridge J, Boos W (1998) Negative transcriptional regulation of a positive regulator: the expression of malT, encoding the transcriptional activator of the maltose regulon of Escherichia coli, is negatively controlled by Mlc. Mol Microbiol 27: 381–390. 9484893 10.1046/j.1365-2958.1998.00694.x

[79] Fox DK, Meadow ND, Roseman S (1986) Phosphate transfer between acetate kinase and enzyme I of the bacterial phosphotransferase system. J Biol Chem 261: 13498–13503. 3020035

[80] Ferea TL, Botstein D, Brown PO, Rosenzweig RF (1999) Systematic changes in gene expression patterns following adaptive evolution in yeast. Proc Natl Acad Sci U S A 96: 9721–9726. 10449761 10.1073/pnas.96.17.9721 PMC22277

[81] Kotlarz D, Garreau H, Buc H (1975) Regulation of the amount and of the activity of phosphofructokinases and pyruvate kinases in Escherichia coli. Biochim Biophys Acta 381: 257–268. 122902 10.1016/0304-4165(75)90232-9

[82] Babul J (1978) Phosphofructokinases from Escherichia coli. Purification and characterization of the nonallosteric isozyme. J Biol Chem 253: 4350–4355. 149128

[83] Nobelmann B, Lengeler JW (1996) Molecular analysis of the gat genes from Escherichia coli and of their roles in galactitol transport and metabolism. J Bacteriol 178: 6790–6795. 8955298 10.1128/jb.178.23.6790-6795.1996 PMC178577

[84] Zhong S, Miller SP, Dykhuizen DE, Dean AM (2009) Transcription, translation, and the evolution of specialists and generalists. Mol Biol Evol 26: 2661–2678. 19706726 10.1093/molbev/msp187 PMC2782325

[85] Zhong S, Khodursky A, Dykhuizen DE, Dean AM (2004) Evolutionary genomics of ecological specialization. Proc Natl Acad Sci U S A 101: 11719–11724. 15289609 10.1073/pnas.0404397101 PMC511043

[86] Morita T, Kawamoto H, Mizota T, Inada T, Aiba H (2004) Enolase in the RNA degradosome plays a crucial role in the rapid decay of glucose transporter mRNA in the response to phosphosugar stress in Escherichia coli. Mol Microbiol 54: 1063–1075. 15522087 10.1111/j.1365-2958.2004.04329.x

[87] Vanderpool CK (2007) Physiological consequences of small RNA-mediated regulation of glucose-phosphate stress. Curr Opin Microbiol 10: 146–151. 17383224 10.1016/j.mib.2007.03.011

[88] Morita T, Maki K, Aiba H (2005) RNase E-based ribonucleoprotein complexes: mechanical basis of mRNA destabilization mediated by bacterial noncoding RNAs. Genes Dev 19: 2176–2186. 16166379 10.1101/gad.1330405 PMC1221888

[89] Vanderpool CK, Gottesman S (2004) Involvement of a novel transcriptional activator and small RNA in post-transcriptional regulation of the glucose phosphoenolpyruvate phosphotransferase system. Mol Microbiol 54: 1076–1089. 15522088 10.1111/j.1365-2958.2004.04348.x

[90] Ikeda Y, Yagi M, Morita T, Aiba H (2011) Hfq binding at RhlB-recognition region of RNase E is crucial for the rapid degradation of target mRNAs mediated by sRNAs in Escherichia coli. Mol Microbiol 79: 419–432. 21219461 10.1111/j.1365-2958.2010.07454.x

[91] Gonidakis S, Finkel SE, Longo VD (2010) Genome-wide screen identifies Escherichia coli TCA-cycle-related mutants with extended chronological lifespan dependent on acetate metabolism and the hypoxia-inducible transcription factor ArcA. Aging Cell 9: 868–881. 20707865 10.1111/j.1474-9726.2010.00618.x PMC2941539

[92] Li M, Ho PY, Yao S, Shimizu K (2006) Effect of lpdA gene knockout on the metabolism in Escherichia coli based on enzyme activities, intracellular metabolite concentrations and metabolic flux analysis by 13C-labeling experiments. J Biotechnol 122: 254–266. 16310273 10.1016/j.jbiotec.2005.09.016

[93] Abdel-Hamid AM, Attwood MM, Guest JR (2001) Pyruvate oxidase contributes to the aerobic growth efficiency of Escherichia coli. Microbiology 147: 1483–1498. 11390679 10.1099/00221287-147-6-1483

[94] Blaschkowski HP, Neuer G, Ludwig-Festl M, Knappe J (1982) Routes of flavodoxin and ferredoxin reduction in Escherichia coli. CoA-acylating pyruvate: flavodoxin and NADPH: flavodoxin oxidoreductases participating in the activation of pyruvate formate-lyase. Eur J Biochem 123: 563–569. 7042345

[95] Eremina NS, Yampolskaya TA, Altman IB, Mashko SV, Stoynova NV (2010) Overexpression of ydbK-encoding Putative Pyruvate Synthase Improves L-valine Production and Aerobic Growth on Ethanol Media by an Escherichia coli Strain Carrying an Oxygen-Resistant Alcohol Dehydrogenase. J Microbial Biochem Technol 2: 077–083.

[96] Akhtar MK, Jones PR (2009) Construction of a synthetic YdbK-dependent pyruvate:H2 pathway in Escherichia coli BL21(DE3). Metab Eng 11: 139–147. 19558967 10.1016/j.ymben.2009.01.002

[97] Sawers G, Hesslinger C, Muller N, Kaiser M (1998) The glycyl radical enzyme TdcE can replace pyruvate formate-lyase in glucose fermentation. J Bacteriol 180: 3509–3516. 9657990 10.1128/jb.180.14.3509-3516.1998 PMC107315

[98] Reizer J, Reizer A, Saier MH Jr (1995) Novel phosphotransferase system genes revealed by bacterial genome analysis–a gene cluster encoding a unique Enzyme I and the proteins of a fructose-like permease system. Microbiology 141 Pt 4: 961–971. 7773398 10.1099/13500872-141-4-961

[99] Pecher A, Blaschkowski HP, Knappe K, Bock A (1982) Expression of pyruvate formate-lyase of Escherichia coli from the cloned structural gene. Arch Microbiol 132: 365–371. 6758723 10.1007/BF00413390

[100] de Graef MR, Alexeeva S, Snoep JL, Teixeira de Mattos MJ (1999) The steady-state internal redox state (NADH/NAD) reflects the external redox state and is correlated with catabolic adaptation in Escherichia coli. J Bacteriol 181: 2351–2357. 10197995 10.1128/jb.181.8.2351-2357.1999 PMC93657

[101] Knappe J, Sawers G (1990) A radical-chemical route to acetyl-CoA: the anaerobically induced pyruvate formate-lyase system of Escherichia coli. FEMS Microbiol Rev 6: 383–398. 2248795 10.1111/j.1574-6968.1990.tb04108.x

[102] Sawers G, Bock A (1989) Novel transcriptional control of the pyruvate formate-lyase gene: upstream regulatory sequences and multiple promoters regulate anaerobic expression. J Bacteriol 171: 2485–2498. 2651404 10.1128/jb.171.5.2485-2498.1989 PMC209925

[103] Alexeeva S, de Kort B, Sawers G, Hellingwerf KJ, de Mattos MJ (2000) Effects of limited aeration and of the ArcAB system on intermediary pyruvate catabolism in Escherichia coli. J Bacteriol 182: 4934–4940. 10940038 10.1128/jb.182.17.4934-4940.2000 PMC111374

[104] Carlson RP (2007) Metabolic systems cost-benefit analysis for interpreting network structure and regulation. Bioinformatics 23: 1258–1264. 17344237 10.1093/bioinformatics/btm082

[105] Sawers G, Watson G (1998) A glycyl radical solution: oxygen-dependent interconversion of pyruvate formate-lyase. Mol Microbiol 29: 945–954. 9767563 10.1046/j.1365-2958.1998.00941.x

[106] Vemuri GN, Altman E, Sangurdekar DP, Khodursky AB, Eiteman MA (2006) Overflow metabolism in Escherichia coli during steady-state growth: transcriptional regulation and effect of the redox ratio. Appl Environ Microbiol 72: 3653–3661. 16672514 10.1128/AEM.72.5.3653-3661.2006 PMC1472329

[107] Cozzone AJ, El-Mansi M (2005) Control of isocitrate dehydrogenase catalytic activity by protein phosphorylation in Escherichia coli. J Mol Microbiol Biotechnol 9: 132–146. 16415587 10.1159/000089642

[108] El-Mansi M, Cozzone AJ, Shiloach J, Eikmanns BJ (2006) Control of carbon flux through enzymes of central and intermediary metabolism during growth of Escherichia coli on acetate. Curr Opin Microbiol 9: 173–179. 16530464 10.1016/j.mib.2006.02.002

[109] el-Mansi EM, Nimmo HG, Holms WH (1986) Pyruvate metabolism and the phosphorylation state of isocitrate dehydrogenase in Escherichia coli. J Gen Microbiol 132: 797–806. 3525743 10.1099/00221287-132-3-797

[110] Li M, Yao S, Shimizu K (2007) Effect of poxB gene knockout on metabolism in Escherichia coli based on growth characteristics and enzyme activities. World Journal of Microbiology and Biotechnology 23: 573–580.

[111] Carlson RP (2009) Decomposition of complex microbial behaviors into resource-based stress responses. Bioinformatics 25: 90–97. 19008248 10.1093/bioinformatics/btn589 PMC2638933

[112] Weidner U, Geier S, Ptock A, Friedrich T, Leif H, et al (1993) The gene locus of the proton-translocating NADH: ubiquinone oxidoreductase in Escherichia coli. Organization of the 14 genes and relationship between the derived proteins and subunits of mitochondrial complex I. J Mol Biol 233: 109–122. 7690854 10.1006/jmbi.1993.1488

[113] Elvin CM, Hardy CM, Rosenberg H (1985) Pi exchange mediated by the GlpT-dependent sn-glycerol-3-phosphate transport system in Escherichia coli. J Bacteriol 161: 1054–1058. 3882662 10.1128/jb.161.3.1054-1058.1985 PMC215006

[114] Deutscher J, Francke C, Postma PW (2006) How phosphotransferase system-related protein phosphorylation regulates carbohydrate metabolism in bacteria. Microbiol Mol Biol Rev 70: 939–1031. 17158705 10.1128/MMBR.00024-06 PMC1698508

[115] Novotny MJ, Frederickson WL, Waygood EB, Saier MH Jr (1985) Allosteric regulation of glycerol kinase by enzyme IIIglc of the phosphotransferase system in Escherichia coli and Salmonella typhimurium. J Bacteriol 162: 810–816. 2985549 10.1128/jb.162.2.810-816.1985 PMC218925

[116] Zwaig N, Lin EC (1966) Feedback inhibition of glycerol kinase, a catabolic enzyme in Escherichia coli. Science 153: 755–757. 5328677 10.1126/science.153.3737.755

[117] de Boer M, Broekhuizen CP, Postma PW (1986) Regulation of glycerol kinase by enzyme IIIGlc of the phosphoenolpyruvate:carbohydrate phosphotransferase system. J Bacteriol 167: 393–395. 3013838 10.1128/jb.167.1.393-395.1986 PMC212891

[118] Lin EC (1976) Glycerol dissimilation and its regulation in bacteria. Annu Rev Microbiol 30: 535–578. 825019 10.1146/annurev.mi.30.100176.002535

[119] Subedi KP, Kim I, Kim J, Min B, Park C (2008) Role of GldA in dihydroxyacetone and methylglyoxal metabolism of Escherichia coli K12. FEMS Microbiol Lett 279: 180–187. 18179582 10.1111/j.1574-6968.2007.01032.x

[120] Applebee MK, Joyce AR, Conrad TM, Pettigrew DW, Palsson BO (2011) Functional and metabolic effects of adaptive glycerol kinase (GLPK) mutants in Escherichia coli. J Biol Chem 286: 23150–23159. 21550976 10.1074/jbc.M110.195305 PMC3123082

[121] Lindner SN, Meiswinkel TM, Panhorst M, Youn JW, Wiefel L, et al (2012) Glycerol-3-phosphatase of Corynebacterium glutamicum. J Biotechnol 159: 216–224. 22353596 10.1016/j.jbiotec.2012.02.003

[122] Rozen DE, Schneider D, Lenski RE (2005) Long-term experimental evolution in Escherichia coli. XIII. Phylogenetic history of a balanced polymorphism. J Mol Evol 61: 171–180. 15999245 10.1007/s00239-004-0322-2

[123] Levine JM, HilleRisLambers J (2009) The importance of niches for the maintenance of species diversity. Nature 461: 254–257. 19675568 10.1038/nature08251

[124] Maharjan RP, Ferenci T, Reeves PR, Li Y, Liu B, et al (2012) The multiplicity of divergence mechanisms in a single evolving population. Genome Biol 13: R41. 22682524 10.1186/gb-2012-13-6-r41 PMC3446313

[125] de Mazancourt C, Schwartz MW (2010) A resource ratio theory of cooperation. Ecol Lett 13: 349–359. 20455920 10.1111/j.1461-0248.2009.01431.x

[126] Updegrove TB, Wartell RM (2011) The influence of Escherichia coli Hfq mutations on RNA binding and sRNA*mRNA duplex formation in rpoS riboregulation. Biochim Biophys Acta 1809: 532–540. 21889623 10.1016/j.bbagrm.2011.08.006

[127] Updegrove T, Wilf N, Sun X, Wartell RM (2008) Effect of Hfq on RprA-rpoS mRNA pairing: Hfq-RNA binding and the influence of the 5′ rpoS mRNA leader region. Biochemistry 47: 11184–11195. 18826256 10.1021/bi800479p

[128] Ko YF, Bentley WE, Weigand WA (1994) A metabolic model of cellular energetics and carbon flux during aerobic Escherichia coli fermentation. Biotechnol Bioeng 43: 847–855. 18615877 10.1002/bit.260430903

[129] van Hoek MJ, Merks RM (2012) Redox balance is key to explaining full vs. partial switching to low-yield metabolism. BMC Syst Biol 6: 22. 22443685 10.1186/1752-0509-6-22 PMC3384451

[130] Tempest D, Neijssel O (1987) Growth Yield and Energy Distribution. In: Neidhardt F, editor. *Escherichia coli* and *Salmonella typhimurium* Washington DC: American Society for Microbiology. pp. 797–806.

[131] Negrete A, Majdalani N, Phue JN, Shiloach J (2013) Reducing acetate excretion from E. coli K-12 by over-expressing the small RNA SgrS. N Biotechnol 30: 269–273. 22107968 10.1016/j.nbt.2011.11.007 PMC3322308

[132] Kawamoto H, Koide Y, Morita T, Aiba H (2006) Base-pairing requirement for RNA silencing by a bacterial small RNA and acceleration of duplex formation by Hfq. Mol Microbiol 61: 1013–1022. 16859494 10.1111/j.1365-2958.2006.05288.x

[133] Piotrowski JS, Nagarajan S, Kroll E, Stanbery A, Chiotti KE, et al (2012) Different selective pressures lead to different genomic outcomes as newly-formed hybrid yeasts evolve. BMC Evol Biol 12: 46. 22471618 10.1186/1471-2148-12-46 PMC3372441

[134] Gause GF (1932) Experimental studies on the struggle for existence: 1. Mixed population of two species of yeast. Journal of Experimental Biology 9: 389–402.

[135] Hardin G (1960) The competitive exclusion principle. Science 131: 1292–1297. 14399717 10.1126/science.131.3409.1292

[136] Crow JF, Kimura K (1965) Evolution in asexual populations. Am Nat 99: 439–450.

[137] Jezequel N, Lagomarsino MC, Heslot F, Thomen P (2013) Long-term diversity and genome adaptation of Acinetobacter baylyi in a minimal-medium chemostat. Genome Biol Evol 5: 87–97. 23254395 10.1093/gbe/evs120 PMC3595037

[138] Morris BE, Henneberger R, Huber H, Moissl-Eichinger C (2013) Microbial syntrophy: interaction for the common good. FEMS Microbiol Rev 37: 384–406. 23480449 10.1111/1574-6976.12019

[139] Seyfried TN, Shelton LM (2010) Cancer as a metabolic disease. Nutrition & metabolism 7: 7. 20181022 10.1186/1743-7075-7-7 PMC2845135

[140] Vaughan RA, Garcia-Smith R, Trujillo KA, Bisoffi M (2013) Tumor necrosis factor alpha increases aerobic glycolysis and reduces oxidative metabolism in prostate epithelial cells. Prostate 73: 1538–1546. 23818177 10.1002/pros.22703

[141] Ciofu O, Mandsberg LF, Bjarnsholt T, Wassermann T, Hoiby N (2010) Genetic adaptation of Pseudomonas aeruginosa during chronic lung infection of patients with cystic fibrosis: strong and weak mutators with heterogeneous genetic backgrounds emerge in mucA and/or lasR mutants. Microbiology 156: 1108–1119. 20019078 10.1099/mic.0.033993-0

[142] Qin X, Zerr DM, McNutt MA, Berry JE, Burns JL, et al (2012) Pseudomonas aeruginosa syntrophy in chronically colonized airways of cystic fibrosis patients. Antimicrob Agents Chemother 56: 5971–5981. 22964251 10.1128/AAC.01371-12 PMC3486535

[143] Helling RB, Kinney T, Adams J (1981) The maintenance of Plasmid-containing organisms in populations of Escherichia coli. J Gen Microbiol 123: 129–141. 7033453 10.1099/00221287-123-1-129

[144] Sarkar S, Ma WT, Sandri GH (1992) On fluctuation analysis: a new, simple and efficient method for computing the expected number of mutants. Genetica 85: 173–179. 1624139 10.1007/BF00120324

[145] Tusher VG, Tibshirani R, Chu G (2001) Significance analysis of microarrays applied to the ionizing radiation response. Proc Natl Acad Sci U S A 98: 5116–5121. 11309499 10.1073/pnas.091062498 PMC33173

[146] Syn CK, Swarup S (2000) A scalable protocol for the isolation of large-sized genomic DNA within an hour from several bacteria. Anal Biochem 278: 86–90. 10640359 10.1006/abio.1999.4410

[147] Li H, Durbin R (2009) Fast and accurate short read alignment with Burrows-Wheeler transform. Bioinformatics 25: 1754–1760. 19451168 10.1093/bioinformatics/btp324 PMC2705234

[148] Li H, Handsaker B, Wysoker A, Fennell T, Ruan J, et al (2009) The Sequence Alignment/Map format and SAMtools. Bioinformatics 25: 2078–2079. 19505943 10.1093/bioinformatics/btp352 PMC2723002

[149] Adams J, Kinney T, Thompson S, Rubin L, Helling RB (1979) Frequency-Dependent Selection for Plasmid-Containing Cells of ESCHERICHIA COLI. Genetics 91: 627–637. 17248902 10.1093/genetics/91.4.627 PMC1216856

