# Supplementary material for: Ex Uno Plures: Clonal Reinforcement Drives Evolution of a Simple Microbial Community
Source: PLoS Genet. 2014 Jun 26;10(6):e1004430. doi: 10.1371/journal.pgen.1004430 (PMC4072538; doi:10.1371/journal.pgen.1004430)
Supplement: Table S1 — Genes and transcription units (T.U.) affected by more than one mutation. (PDF) [file pgen.1004430.s001.pdf]

Table S1. Genes and transcription units (T.U.) affected by more than one mutation

# hits	Strain	T.U.	Genes	amino acid change (codon change)	Gene products
2	CV103	acrD	**acrD** (x2)	A177S (GCC→TCC), S389I (AGC→ATC)	AcrAD-TolC multidrug efflux transport system
2	CV103	caiTABCDE	**caiT** (x2)	Q330K (CAG→AAG), W107C (TGG→TGT)	L-carnitine : γ-butyrobetaine antiporter
2	CV103	cmtBA-yggPFDC	**yggP** (x2)	A279A (GCG→GCT), A125S (GCC→TCC)	predicted dehydrogenase
2	CV103	cobUST	**cobT** (x2)	A322E (GCA→GAA), A106A (GCG→GCT)	nicotinate-nucleotide dimethylbenzimidazole phosphoribosyltransferase
2	CV103	envY-ompT	**ompT** (x2)	G202V (GGC→GTC), E187* (GAA→TAA)	outer membrane protease VII
2	CV103	ggt	**ggt** (x2)	Q542H (CAG→CAT), T416T (ACG→ACT)	γ-glutamyltranspeptidase
2	CV103	htrG-cca	**cca** (x2)	G149C (GGT→TGT), M241I (ATG→ATT)	fused tRNA nucleotidyltransferase / 2',3'-cyclic phosphodiesterase / 2' nucleotidase-phosphatase
5	CV103	hypABCDE-fhlA	**fhlA** (x2), **hypD**(x2), hypE	A54E (GCG→GAG), E76* (GAA→TAA); V83V (GTG→GTT), L295L (CTG→CTT); G103* (GGA→TGA)	FhlA-Formate DNA-binding transcriptional activator; part of HypCD complex involved in hydrogenase maturation
2	CV103	lptFG	**lptG** (x2)	E164* (GAG→TAG), R165L (CGG→CTG)	lipopolysaccharide transport system
2	CV103	maeA	**maeA** (x2)	P409T (CCG→ACG), I231I (ATC→ATA)	malate dehydrogenase, NAD-requiring
2	CV103	malEFG	**malG** (x2)	G179C (GGC→TGC), G169V (GGG→GTG)	maltose ABC transporter
2	CV103	mntR-ybiR	**ybiR** (x2)	A167D (GCT→GAT), P298T (CCG→ACG)	predicted transporter
2	CV103	nanCM	**nanM** (x2)	T166T (ACC→ACA), P125P (CCG→CCT)	N-acetylneuraminate mutarotase
2	CV103	napFDAGHBC-ccmABCDEFGH	**napH** (x2)	P162H (CCC→CAC), D61Y (GAC→TAC)	periplasmic nitrate reductase
2	CV103	ptsHI-crr	**ptsI** (x2)	A328S (GCG→TCG), M518I (ATG→ATT)	phosphoenolpyruvate-protein phosphotransferase PtsI, PTS enzyme I
2	CV103	pyrG-eno	**eno** (x2)	L60L (CTG→CTT), A37S (GCT→TCT)	enzyme, interconversion of 2-phosphoglycerate and phosphoenolpyruvate
2	CV103	tamAB-ytfP	**tamB** (x2)	D141Y (GAC→TAC), Q288H (CAG→CAT)	translocation and assembly module
3	CV103	wza-wzb-wzc-wcaAB	**wza** (x2), wzc	**wza:** K313N (AAG→AAT), R223S (CGC→AGC); **wzc:** A270A (GCC→GCA)	capsular polysaccharide export apparatus (x2)
2	CV103	ybiP	**ybiP** (x2)	Q302H (CAG→CAT), G123G (GGC→GGA)	predicted hydrolase, inner membrane
2	CV103	ydcSTUV	**ydcS** (x2)	S94Y (TCC→TAC), S254Y (TCC→TAC)	YdcS/YdcT/YdcV/YdcU ABC transporter
2	CV103	yfcUTSRQPO	**yfcP** (x2)	G127V (GGA→GTA), G85W (GGG→TGG)	predicted fimbrial-like adhesin protein
2	CV103	yihPO	**yihO** (x2), yihP	**yihO** : A257E (GCG→GAG), A244S (GCT→TCT); **yihP** : R154L (CGC→CTC)	YihO galactose-pentose-hexuronide transporter (x2)
3	CV103	evgAS	**evgS** (x3)	R513S (CGC→AGC), R887L (CGC→CTC), L1063M (CTG→ATG)	sensory histidine kinase

3	CV103	fliLMNOPQR	**fliM** (x3)	E59D (GAG→GAT), A62S (GCC→TCC), E178* (GAG→TAG)	flagellar motor switch protein
2	CV103	agaSYBCDI	agaC, agaS	**agaC**: G195G (GGC→GGA); **agaS**: R38L (CGT→CTT)	galactosamine PTS permease – cryptic; putative tagatose-6-phosphate aldose/ketose isomerase
2	CV103	ddpXABCDF	ddpB, ddpF	**ddpB**: P98P (CCG→CCT); **ddpF**: L302I (CTC→ATC)	YddO/YddP/YddQ/YddR/YddS ABC transporter (x2)
2	CV103	fdrA-ylbE_1E_2F-ybcF	ybcF, ylbE_1	**ybcF**: M87I (ATG→ATT); **ylbE_1**: C→A nt 694 (pseudogene)	predicted carbamate kinase; predicted protein, N-ter fragment
2	CV103	fliFGHIJK	fliF, fliH	**fliF**: A28A (GCC→GCA); **fliH**: E41D (GAG→GAT)	flagellar M-ring protein; flagellar biosynthesis protein
2	CV103	gspCDEFGHIJKLMO	gspF, gspG	**gspF**: R266L (CGC→CTC); **gspG**: E53* (GAA→TAA)	GspC-O secreton complex (x2)
2	CV103	hcaEFCBD	hcaD, hcaE	**hcaD**: A387A (GCG→GCT); **hcaE**: R299S (CGC→AGC)	3-phenylpropionate dioxygenase, predicted ferredoxin reductase subunit; α subunit of same
2	CV103	insC-5D-5-yqiGHI-insCD-5	yqiH, yqiI	**yqiH**: G186W (GGG→TGG); **yqiI**: P151T (CCG→ACG)	predicted periplasmic pilin chaperone; protein involved in detoxification of methylglyoxal
2	CV103	paaABCDEFGHIJK	paaJ, paaK	**paaJ**: S198* (TCA→TAA); **paaK**: F44L (TTC→TTA)	β-ketoadipyl-CoA thiolase; phenylacetate-CoA ligase
2	CV103	rbsDACBKR	rbsC, rbsK	**rbsC**: A257S (GCT→TCT); **rbsK**: P272T (CCA→ACA)	ribose ABC transporter; ribokinase
2	CV103	wcaCDEF-gmd-fcl-gmm-wcaI-cpsBG-wcaJ-wzxC	fcl, wcaE	**fcl**: R20M (AGG→ATG); **wcaE**: S201Y (TCT→TAT)	GDP-fucose synthase; predicted colanic acid biosynthesis glycosyl transferase
2	CV103	ydbK-ompN	ompN, ydbK	**ompN:** S221Y (TCT→TAT); **ydbK**: R539L (CGC→CTC)	outer membrane pore protein N, non-specific; predicted pyruvate:flavodoxin oxidoreductase
3	CV103	yfaXWVU	yfaV, yfaW, yfaX	**yfaV**: A403A (GCG→GCT); **yfaW**: P351T (CCG→ACG); **yfaX**: A203S (GCT→TCT)	predicted transporter; L-rhamnonate dehydratase; predicted DNA-binding transcriptional regulator
2	CV103	ygfB-pepP-ubiH-visC	pepP, ubiH	**pepP**: W89L (TGG→TTG); **ubiH**: G320V (GGA→GTA)	proline aminopeptidase P II; 2-octaprenyl-6-methoxyphenol hydroxylase
2	CV103	yjeFE-amiB-mutL-miaA-hfq-hflXKC	hflC, hfq	**hflC**: G188G (GGC→GGA); **hfq**: Q52H (CAG→CAT)	regulator of FtsH protease; HF-I, host factor for RNA phage Q β replication
2	CV101	hybOABCDEFG	hybE, hybO	**hybE**: G123D (GGC→GAC); **hybO**: G172G (GGC→GGT)	hydrogenase 2-specific chaperone; hydrogenase 2, small subunit
2	CV115-CV116	fdoGHI-fdhE	fdhE, fdoH	**fdhE**: E136D (GAG→GAT); **fdoH**: L260I (CTC→ATC)	formate dehydrogenase formation protein; formate dehydrogenase-O, β subunit
2	CV115-CV116	smtA-mukFEB (smtAp)	**mukB** (x2)	G34G (GGC→GGA), A1057E (GCG→GAG)	cell division protein involved in chromosome partitioning
2	CV115-CV116	yphG	**yphG** (x2)	A527E (GCG→GAG), L347F (TTG→TTT)	conserved protein
2	CV103/CV101-CV115-CV116	rihA	**rihA** (x2)	103 P95P (CCG→CCT), 116 V20F (GTT→TTT)	ribonucleoside hydrolase 1 (pyrimidine-specific)
2	CV103/CV115-CV116	sgrR-sroA-tbpA-thiPQ	**tbpA** (x2)	103 G81W (GGG→TGG); 116 N279K (AAC→AAA)	thiamin ABC transporter - periplasmic binding protein
4	CV103/CV115	nuoABCEFGHIJ	**nuoE** (x2), nuoI,	**nuoE**: 103 L14L	NADH:ubiquinone

	-CV116	KLMN	nuoM	(CTG→CTT),<br>116 A17E (GCA→GAA);<br>**nuoI**: 103 R93L<br>(CGC→CTC);<br>**nuoM:** 103 L336F<br>(TTG→TTT)	oxidoreductase I (x3)
4	CV103/CV115<br>-CV116	ptrA-recBD	ptrA, **recB** (x2),<br>recD	**ptrA**: 103 S753I<br>(AGC→ATC);<br>**recB**:103 G241C<br>(GGT→TGT),<br>116 L979L (CTC→CTA);<br>**recD**: 103 L37L (CTC→CTA)	protease III; component of the RecBCD helicase/nuclease complex (x2)
3	CV103/CV115<br>-CV116	rplKAJL-rpoBC	**rpoB** (x2), rpoC	**rpoB**: 103 I177I<br>(ATC→ATA),<br>116 P567P (CCG→CCT);<br>**rpoC**: 103 V526V<br>(GTG→GTT)	RNA polymerase, β subunit;<br>RNA polymerase, β' subunit
2	CV103/CV115<br>-CV116	bamB-der	**der** (x2)	103 I266I (ATC→ATA),<br>116 A412D (GCC→GAC)	50S ribosomal subunit stability factor
2	CV103/CV115<br>-CV116	csiD-ygaF-<br>gabDTP	**gabP** (x2)	103 Q3K (CAA→AAA),<br>116 T93N (ACC→AAC)	4-aminobutyrate:H^+ symporter
2	CV103/CV115<br>-CV116	fucAO	**fucO** (x2)	103 P126T (CCG→ACG),<br>116 G107V (GGC→GTC)	L-1,2-propanediol oxidoreductase
2	CV103/CV115<br>-CV116	yhjJ	**yhjJ** (x2)	103 S427I (AGC→ATC),<br>116 Q28H (CAG→CAT)	predicted zinc-dependent peptidase
2	CV103/CV115<br>-CV116	glmUS	**glmS** (x2)	103 G593G (GGC→GGA),<br>116 A9S (GCG→TCG)	L-glutamine:D-fructose-6-phosphate aminotransferase
