# Supplementary material for: Ex Uno Plures: Clonal Reinforcement Drives Evolution of a Simple Microbial Community
Source: PLoS Genet. 2014 Jun 26;10(6):e1004430. doi: 10.1371/journal.pgen.1004430 (PMC4072538; doi:10.1371/journal.pgen.1004430)
Supplement: Table S2 — All mutations. (PDF) [file pgen.1004430.s002.pdf]

Table S2. All mutations

cumulative position relative to *E. coli* K12 MG1655	gene	description	annotation	K12	JA122	CV101	CV103	CV115	CV116
310	thrL/thrA	thr operon leader peptide/fused aspartokinase I and homoserine dehydrogenase I	intergenic (+55/‑27)		Δ1 bp	Δ1 bp	Δ1 bp	Δ1 bp	Δ1 bp
393	thrA	fused aspartokinase I and homoserine dehydrogenase I	R19R (CGT→CGG)	T	G	G	G	G	G
588	thrA	fused aspartokinase I and homoserine dehydrogenase I	G84G (GGG→GGA)	G	A	A	A	A	A
774	thrA	fused aspartokinase I and homoserine dehydrogenase I	T146T (ACT→ACC)	T	C	C	C	C	C
867	thrA	fused aspartokinase I and homoserine dehydrogenase I	S177S (AGC→AGT)	C	T	T	T	T	T
939	thrA	fused aspartokinase I and homoserine dehydrogenase I	V201V (GTG→GTA)	G	A	A	A	A	A
966	thrA	fused aspartokinase I and homoserine dehydrogenase I	S210S (TCT→TCC)	T	C	C	C	C	C
969	thrA	fused aspartokinase I and homoserine dehydrogenase I	A211A (GCT→GCG)	T	G	G	G	G	G
1,206	thrA	fused aspartokinase I and homoserine dehydrogenase I	A290A (GCA→GCT)	A	T	T	T	T	T
1,278	thrA	fused aspartokinase I and homoserine dehydrogenase I	N314N (AAC→AAT)	C	T	T	T	T	T
1,299	thrA	fused aspartokinase I and homoserine dehydrogenase I	S321S (TCT→TCC)	T	C	C	C	C	C
1,302	thrA	fused aspartokinase I and homoserine dehydrogenase I	G322G (GGT→GGC)	T	C	C	C	C	C
1,323	thrA	fused aspartokinase I and homoserine dehydrogenase I	V329V (GTC→GTT)	C	T	T	T	T	T
1,341	thrA	fused aspartokinase I and homoserine dehydrogenase I	V335V (GTC→GTG)	C	G	G	G	G	G
1,407	thrA	fused aspartokinase I and homoserine dehydrogenase I	S357S (AGC→AGT)	C	T	T	T	T	T
1,425	thrA	fused aspartokinase I and homoserine dehydrogenase I	P363P (CCA→CCG)	A	G	G	G	G	G
1,509	thrA	fused aspartokinase I and homoserine dehydrogenase I	A391A (GCA→GCG)	A	G	G	G	G	G
1,593	thrA	fused aspartokinase I and homoserine dehydrogenase I	A419A (GCA→GCG)	A	G	G	G	G	G
1,797	thrA	fused aspartokinase I and homoserine dehydrogenase I	Q487Q (CAG→CAA)	G	A	A	A	A	A
1,860	thrA	fused aspartokinase I and homoserine dehydrogenase I	A508A (GCT→GCA)	T	A	A	A	A	A
1,875	thrA	fused aspartokinase I and homoserine dehydrogenase I	V513V (GTA→GTG)	A	G	G	G	G	G
1,884	thrA	fused aspartokinase I and homoserine dehydrogenase I	L516L (CTT→CTA)	T	A	A	A	A	A
2,038	thrA	fused aspartokinase I and homoserine dehydrogenase I	L568L (CTG→TTG)	C	T	T	T	T	T
2,108	thrA	fused aspartokinase I and homoserine dehydrogenase I	Q591L (CAG→CTG)	A	T	T	T	T	T
2,116	thrA	fused aspartokinase I and homoserine dehydrogenase I	Y594H (TAT→CAT)	T	C	C	C	C	C
2,211	thrA	fused aspartokinase I and homoserine dehydrogenase I	A625A (GCA→GCT)	A	T	T	T	T	T
2,247	thrA	fused aspartokinase I and homoserine dehydrogenase I	S637S (TCT→TCA)	T	A	A	A	A	A
2,307	thrA	fused aspartokinase I and homoserine dehydrogenase I	T657T (ACC→ACT)	C	T	T	T	T	T
2,310	thrA	fused aspartokinase I and homoserine dehydrogenase I	T658T (ACG→ACT)	G	T	T	T	T	T
2,343	thrA	fused aspartokinase I and homoserine dehydrogenase I	D669D (GAC→GAT)	C	T	T	T	T	T
2,373	thrA	fused aspartokinase I and homoserine dehydrogenase I	V679V (GTG→GTA)	G	A	A	A	A	A
2,382	thrA	fused aspartokinase I and homoserine dehydrogenase I	K682K (AAA→AAG)	A	G	G	G	G	G
2,469	thrA	fused aspartokinase I and homoserine dehydrogenase I	A711A (GCC→GCT)	C	T	T	T	T	T

2,508	thrA	fused aspartokinase I and homoserine dehydrogenase I	Q724Q (CAA→CAG)	A	G	G	G	G	G
2,595	thrA	fused aspartokinase I and homoserine dehydrogenase I	G753G (GGC→GGT)	C	T	T	T	T	T
2,597	thrA	fused aspartokinase I and homoserine dehydrogenase I	V754A (GTC→GCC)	T	C	C	C	C	C
2,676	thrA	fused aspartokinase I and homoserine dehydrogenase I	F780F (TTC→TTT)	C	T	T	T	T	T
2,709	thrA	fused aspartokinase I and homoserine dehydrogenase I	V791V (GTA→GTG)	A	G	G	G	G	G
2,760	thrA	fused aspartokinase I and homoserine dehydrogenase I	A808A (GCT→GCC)	T	C	C	C	C	C
3,025	thrB	homoserine kinase	E75E (GAA→GAG)	A	G	G	G	G	G
3,028	thrB	homoserine kinase	L76L (CTG→CTT)	G	T	T	T	T	T
3,031	thrB	homoserine kinase	G77G (GGT→GGC)	T	C	C	C	C	C
3,055	thrB	homoserine kinase	T85T (ACC→ACT)	C	T	T	T	T	T
3,073	thrB	homoserine kinase	P91P (CCG→CCA)	G	A	A	A	A	A
3,097	thrB	homoserine kinase	S99S (AGT→AGC)	T	C	C	C	C	C
3,142	thrB	homoserine kinase	C114C (TGC→TGT)	C	T	T	T	T	T
3,196	thrB	homoserine kinase	G132G (GGC→GGA)	C	A	A	A	A	A
3,199	thrB	homoserine kinase	R133R (CGT→CGA)	T	A	A	A	A	A
3,244	thrB	homoserine kinase	L148L (CTC→CTT)	C	T	T	T	T	T
3,424	thrB	homoserine kinase	A208A (GCA→GCT)	A	T	T	T	T	T
3,520	thrB	homoserine kinase	P240P (CCA→CCT)	A	T	T	T	T	T
3,622	thrB	homoserine kinase	E274D (GAA→GAT)	A	T	T	T	T	T
3,763	thrC	threonine synthase	N10N (AAC→AAT)	C	T	T	T	T	T
3,937	thrC	threonine synthase	P68P (CCA→CCG)	A	G	G	G	G	G
4,102	thrC	threonine synthase	G123G (GGT→GGC)	T	C	C	C	C	C
4,336	thrC	threonine synthase	E201E (GAA→GAG)	A	G	G	G	G	G
4,351	thrC	threonine synthase	L206L (CTA→CTG)	A	G	G	G	G	G
4,363	thrC	threonine synthase	S210S (TCG→TCA)	G	A	A	A	A	A
4,420	thrC	threonine synthase	A229A (GCT→GCA)	T	A	A	A	A	A
4,441	thrC	threonine synthase	coding (708‑709/1287 nt)		2 bp→AG	2 bp→AG	2 bp→AG	2 bp→AG	2 bp→AG
4,780	thrC	threonine synthase	T349T (ACT→ACC)	T	C	C	C	C	C
4,804	thrC	threonine synthase	A357A (GCT→GCG)	T	G	G	G	G	G
4,822	thrC	threonine synthase	D363D (GAT→GAC)	T	C	C	C	C	C
5,067	thrC/yaaX	threonine synthase/predicted protein	intergenic (+47/‑167)		Δ1 bp	Δ1 bp	Δ1 bp	Δ1 bp	Δ1 bp
5,099	thrC/yaaX	threonine synthase/predicted protein	intergenic (+79/‑135)	T	C	C	C	C	C
5,171	thrC/yaaX	threonine synthase/predicted protein	intergenic (+151/‑63)	C	T	T	T	T	T
5,183	thrC/yaaX	threonine synthase/predicted protein	intergenic (+163/‑51)	A	C	C	C	C	C
5,234	yaaX	predicted protein	M1M (GTG→ATG)	G	A	A	A	A	A
5,603	yaaX/yaaA	predicted protein/Peroxide resistance protein, lowers intracellular iron	intergenic (+73/+80)	T	G	G	G	G	G
5,642	yaaX/yaaA	predicted protein/Peroxide resistance protein, lowers intracellular iron	intergenic (+112/+41)	A	C	C	C	C	C
5,732	yaaA	Peroxide resistance protein, lowers intracellular iron	D243A (GAT→GCT)	T	G	G	G	G	G
5,806	yaaA	Peroxide resistance protein, lowers intracellular iron	F218F (TTC→TTT)	G	A	A	A	A	A

5,851	yaaA	Peroxide resistance protein, lowers intracellular iron	I203I (ATC→ATA)	G	T	T	T	T	T
6,106	yaaA	Peroxide resistance protein, lowers intracellular iron	L118L (CTC→CTG)	G	C	C	C	C	C
6,239	yaaA	Peroxide resistance protein, lowers intracellular iron	A74E (GCG→GAG)	G	T	T	T	T	T
6,349	yaaA	Peroxide resistance protein, lowers intracellular iron	E37E (GAG→GAA)	C	T	T	T	T	T
6,502	yaaA/yaaJ	Peroxide resistance protein, lowers intracellular iron/predicted transporter	intergenic (-43/+27)	T	A	A	A	A	A
6,574	yaaJ	predicted transporter	G462G (GGT→GGC)	A	G	G	G	G	G
6,595	yaaJ	predicted transporter	P455P (CCA→CCG)	T	C	C	C	C	C
6,748	yaaJ	predicted transporter	S404S (AGC→AGT)	G	A	A	A	A	A
6,850	yaaJ	predicted transporter	Y370Y (TAT→TAC)	A	G	G	G	G	G
6,898	yaaJ	predicted transporter	L354L (CTT→CTC)	A	G	G	G	G	G
6,933	yaaJ	predicted transporter	L343L (CTA→TTA)	G	A	A	A	A	A
7,132	yaaJ	predicted transporter	G276G (GGT→GGG)	A	C	C	C	C	C
7,939	yaaJ	predicted transporter	F7F (TTC→TTT)	G	A	A	A	A	A
8,963	talB	transaldolase B	L242L (CTG→CTT)	G	T	T	T	T	T
8,975	talB	transaldolase B	coding (738-739/954 nt)		2 bp→TA	2 bp→TA	2 bp→TA	2 bp→TA	2 bp→TA
8,996	talB	transaldolase B	A253A (GCG→GCT)	G	T	T	T	T	T
9,488	mog	molybdochelatase incorporating molybdenum into molybdopterin	L61L (CTG→CTA)	G	A	A	A	A	A
9,623	mog	molybdochelatase incorporating molybdenum into molybdopterin	I106I (ATC→ATA)	C	A	A	A	A	A
9,665	mog	molybdochelatase incorporating molybdenum into molybdopterin	V120V (GTG→GTT)	G	T	T	T	T	T
9,668	mog	molybdochelatase incorporating molybdenum into molybdopterin	G121G (GGC→GGT)	C	T	T	T	T	T
9,754	mog	molybdochelatase incorporating molybdenum into molybdopterin	E150A (GAG→GCG)	A	C	C	C	C	C
9,839	mog	molybdochelatase incorporating molybdenum into molybdopterin	P178P (CCG→CCA)	G	A	A	A	A	A
9,851	mog	molybdochelatase incorporating molybdenum into molybdopterin	A182A (GCA→GCT)	A	T	T	T	T	T
10,006	yaaH	inner membrane protein, Grp1_Fun34_YaaH family	S163S (AGC→AGT)	G	A	A	A	A	A
10,015	yaaH	inner membrane protein, Grp1_Fun34_YaaH family	C160C (TGC→TGT)	G	A	A	A	A	A
10,024	yaaH	inner membrane protein, Grp1_Fun34_YaaH family	G157G (GGG→GGT)	C	A	A	A	A	A
10,099	yaaH	inner membrane protein, Grp1_Fun34_YaaH family	T132T (ACC→ACT)	G	A	A	A	A	A
10,260	yaaH	inner membrane protein, Grp1_Fun34_YaaH family	L79L (CTG→TTG)	G	A	A	A	A	A
10,336	yaaH	inner membrane protein, Grp1_Fun34_YaaH family	A53A (GCT→GCC)	A	G	G	G	G	G
10,548	yaaH/yaaW	inner membrane protein, Grp1_Fun34_YaaH family/conserved protein, UPF0174 family	intergenic (-54/+95)	T	A	A	A	A	A
10,637	yaaH/yaaW	inner membrane protein, Grp1_Fun34_YaaH family/conserved protein, UPF0174 family	intergenic (-143/+6)	C	A	A	A	A	A
10,642	yaaH/yaaW	inner membrane protein, Grp1_Fun34_YaaH family/conserved protein, UPF0174 family	intergenic (-148/+1)	A	G	G	G	G	G
10,703	yaaW	conserved protein, UPF0174 family	T218T (ACG→ACA)	C	T	T	T	T	T
10,718	yaaW	conserved protein, UPF0174 family	S213S (AGC→AGT)	G	A	A	A	A	A
10,732	yaaW	conserved protein, UPF0174 family	A209S (GCG→TCG)	C		A			
10,747	yaaW	conserved protein, UPF0174 family	L204L (CTA→TTA)	G	A	A	A	A	A
10,802	yaaW	conserved protein, UPF0174 family	V185V (GTA→GTG)	T	C	C	C	C	C

10,840	yaaW	conserved protein, UPF0174 family	L173L (CTG→TTG)	G	A	A	A	A	A
10,865	yaaW	conserved protein, UPF0174 family	G164G (GGT→GGC)	A	G	G	G	G	G
10,973	yaaW	conserved protein, UPF0174 family	K128N (AAA→AAT)	T	A	A	A	A	A
10,985	yaaW	conserved protein, UPF0174 family	N124N (AAT→AAC)	A	G	G	G	G	G
11,021	yaaW	conserved protein, UPF0174 family	E112E (GAA→GAG)	T	C	C	C	C	C
11,063	yaaW	conserved protein, UPF0174 family	K98K (AAG→AAA)	C	T	T	T	T	T
11,156	yaaW	conserved protein, UPF0174 family	A67A (GCC→GCT)	G	A	A	A	A	A
11,225	yaaW	conserved protein, UPF0174 family	M44I (ATG→ATA)	C	T	T	T	T	T
11,366	yaaW/yaaI	conserved protein, UPF0174 family/conserved protein, UPF0412 family	intergenic (‑10/+16)	C	T	T	T	T	T
11,439	yaaI	conserved protein, UPF0412 family	S116S (TCG→TCC)	C	G	G	G	G	G
11,481	yaaI	conserved protein, UPF0412 family	S102S (AGC→AGT)	G	A	A	A	A	A
11,541	yaaI	conserved protein, UPF0412 family	S82S (AGC→AGT)	G	A	A	A	A	A
11,695	yaaI	conserved protein, UPF0412 family	A31V (GCC→GTC)	G	A	A	A	A	A
11,814	yaaI/dnaK	conserved protein, UPF0412 family/chaperone Hsp70, co‑chaperone with DnaJ	intergenic (‑28/‑349)	G	A	A	A	A	A
11,854	yaaI/dnaK	conserved protein, UPF0412 family/chaperone Hsp70, co‑chaperone with DnaJ	intergenic (‑68/‑309)	A	G	G	G	G	G
11,861	yaaI/dnaK	conserved protein, UPF0412 family/chaperone Hsp70, co‑chaperone with DnaJ	intergenic (‑75/‑302)	C	T	T	T	T	T
11,925	yaaI/dnaK	conserved protein, UPF0412 family/chaperone Hsp70, co‑chaperone with DnaJ	intergenic (‑139/‑238)	A	C	C	C	C	C
12,026	yaaI/dnaK	conserved protein, UPF0412 family/chaperone Hsp70, co‑chaperone with DnaJ	intergenic (‑240/‑137)	T	G	G	G	G	G
12,237	dnaK	chaperone Hsp70, co‑chaperone with DnaJ	R25R (CGC→CGT)	C	T	T	T	T	T
12,309	dnaK	chaperone Hsp70, co‑chaperone with DnaJ	L49L (CTA→CTG)	A	G	G	G	G	G
12,357	dnaK	chaperone Hsp70, co‑chaperone with DnaJ	T65T (ACT→ACC)	T	C	C	C	C	C
12,384	dnaK	chaperone Hsp70, co‑chaperone with DnaJ	G74G (GGT→GGC)	T	C	C	C	C	C
12,507	dnaK	chaperone Hsp70, co‑chaperone with DnaJ	I115I (ATT→ATC)	T	C	C	C	C	C
12,714	dnaK	chaperone Hsp70, co‑chaperone with DnaJ	G184G (GGC→GGT)	C	T	T	T	T	T
12,846	dnaK	chaperone Hsp70, co‑chaperone with DnaJ	G228G (GGG→GGT)	G	T	T	T	T	T
12,879	dnaK	chaperone Hsp70, co‑chaperone with DnaJ	Y239Y (TAT→TAC)	T	C	C	C	C	C
13,014	dnaK	chaperone Hsp70, co‑chaperone with DnaJ	P284P (CCA→CCG)	A	G	G	G	G	G
13,029	dnaK	chaperone Hsp70, co‑chaperone with DnaJ	D289D (GAC→GAT)	C	T	T	T	T	T
13,320	dnaK	chaperone Hsp70, co‑chaperone with DnaJ	V386V (GTA→GTG)	A	G	G	G	G	G
13,539	dnaK	chaperone Hsp70, co‑chaperone with DnaJ	L459L (CTA→CTG)	A	G	G	G	G	G
13,866	dnaK	chaperone Hsp70, co‑chaperone with DnaJ	A568A (GCG→GCA)	G	A	A	A	A	A
13,932	dnaK	chaperone Hsp70, co‑chaperone with DnaJ	E590E (GAA→GAG)	A	G	G	G	G	G
14,223	dnaJ	chaperone Hsp40, co‑chaperone with DnaK	R19H (CGT→CAT)	G	A	A	A	A	A
14,232	dnaJ	chaperone Hsp40, co‑chaperone with DnaK	R22K (AGA→AAA)	G	A	A	A	A	A
14,401	dnaJ	chaperone Hsp40, co‑chaperone with DnaK	G78G (GGC→GGT)	C	T	T	T	T	T
14,647	dnaJ	chaperone Hsp40, co‑chaperone with DnaK	T160T (ACT→ACC)	T	C	C	C	C	C

14,686	dnaJ	chaperone Hsp40, co-chaperone with DnaK	R173R (CGC→CGT)	C	T	T	T	T	T
14,698	dnaJ	chaperone Hsp40, co-chaperone with DnaK	F177F (TTC→TTT)	C	T	T	T	T	T
14,701	dnaJ	chaperone Hsp40, co-chaperone with DnaK	A178A (GCT→GCC)	T	C	C	C	C	C
14,704	dnaJ	chaperone Hsp40, co-chaperone with DnaK	V179V (GTA→GTG)	A	G	G	G	G	G
14,956	dnaJ	chaperone Hsp40, co-chaperone with DnaK	L263L (CTG→CTC)	G	C	C	C	C	C
15,073	dnaJ	chaperone Hsp40, co-chaperone with DnaK	L302L (CTA→CTT)	A	T	T	T	T	T
15,172	dnaJ	chaperone Hsp40, co-chaperone with DnaK	E335E (GAA→GAG)	A	G	G	G	G	G
15,174	dnaJ	chaperone Hsp40, co-chaperone with DnaK	R336K (AGG→AAG)	G	A	A	A	A	A
15,226	dnaJ	chaperone Hsp40, co-chaperone with DnaK	G353G (GGC→GGT)	C	T	T	T	T	T
15,328	dnaJ/insL	chaperone Hsp40, co-chaperone with DnaK/IS186 transposase	intergenic (+30/-117)	C	T	T	T	T	T
15,338	dnaJ/insL	chaperone Hsp40, co-chaperone with DnaK/IS186 transposase	intergenic (+40/-107)	A	T	T	T	T	T
15,369	dnaJ/insL	chaperone Hsp40, co-chaperone with DnaK/IS186 transposase	intergenic (+71/-76)	T	A	A	A	A	A
27,689	rihC	ribonucleoside hydrolase 3	L133*I* (CTT→ATT)	C				A	A
28,943	dapB	dihydrodipicolinate reductase	A190A (GCG→GCT)	G			T		
32,581	carB	carbamoyl-phosphate synthase large subunit	L589M (CTG→ATG)	C			A		
40,944	caiT	predicted transporter	Q330K (CAG→AAG)	G			T		
41,611	caiT	predicted transporter	W107C (TGG→TGT)	C			A		
46,523	yaaU	predicted transporter	R239R (CGC→CGA)	C			A		
47,713	kefF	potassium-efflux system ancillary protein for KefC, glutathione-regulated; quinone oxidoreductase, FMN-dependent	L156L (CTC→CTA)	C			A		
49,254	kefC	potassium:proton antiporter	R496S (CGC→AGC)	C	A	A	A	A	A
54,440	surA	peptidyl-prolyl cis-trans isomerase (PPIase)	G88V (GGA→GTA)	C			A		
63,241	rapA	RNA polymerase-associated helicase protein (ATPase and RNA polymerase recycling factor)	R8R (CGC→CGG)	G	C	C	C	C	C
63,387	rapA/polB	RNA polymerase-associated helicase protein (ATPase and RNA polymerase recycling factor)/DNA polymerase II	intergenic (-123/+42)	C		A			
64,536	polB	DNA polymerase II	V415V (GTG→GTA)	C	T	T	T	T	T
64,539	polB	DNA polymerase II	S414S (TCA→TCG)	T	C	C	C	C	C
64,593	polB	DNA polymerase II	P396P (CCG→CCA)	C	T	T	T	T	T
64,635	polB	DNA polymerase II	H382H (CAT→CAC)	A	G	G	G	G	G
64,641	polB	DNA polymerase II	R380R (CGA→CGT)	T	A	A	A	A	A
64,644	polB	DNA polymerase II	P379P (CCG→CCT)	C	A	A	A	A	A
64,665	polB	DNA polymerase II	A372A (GCA→GCG)	T	C	C	C	C	C
64,677	polB	DNA polymerase II	G368G (GGT→GGA)	A	T	T	T	T	T
64,680	polB	DNA polymerase II	G367G (GGC→GGG)	G	C	C	C	C	C
64,692	polB	DNA polymerase II	V363V (GTG→GTA)	C	T	T	T	T	T
64,749	polB	DNA polymerase II	H344H (CAC→CAT)	G	A	A	A	A	A
64,755	polB	DNA polymerase II	I342I (ATC→ATT)	G	A	A	A	A	A
64,794	polB	DNA polymerase II	A329A (GCA→GCG)	T	C	C	C	C	C
64,883	polB	DNA polymerase II	L300L (CTA→TTA)	G	A	A	A	A	A

64,896	polB	DNA polymerase II	T295T (ACT→ACC)	A	G	G	G	G	G
65,028	polB	DNA polymerase II	S251S (AGC→AGT)	G	A	A	A	A	A
65,031	polB	DNA polymerase II	N250N (AAT→AAC)	A	G	G	G	G	G
65,057	polB	DNA polymerase II	L242I (CTT→ATT)	G	T	T	T	T	T
65,169	polB	DNA polymerase II	L204L (TTG→TTA)	C	T	T	T	T	T
65,171	polB	DNA polymerase II	L204L (TTG→CTG)	A	G	G	G	G	G
65,202	polB	DNA polymerase II	F193F (TTC→TTT)	G	A	A	A	A	A
65,208	polB	DNA polymerase II	L191L (CTT→CTC)	A	G	G	G	G	G
65,213	polB	DNA polymerase II	S190A (TCG→GCG)	A	C	C	C	C	C
65,232	polB	DNA polymerase II	P183P (CCG→CCA)	C	T	T	T	T	T
65,316	polB	DNA polymerase II	I155I (ATA→ATT)	T	A	A	A	A	A
65,381	polB	DNA polymerase II	T134A (ACT→GCT)	T	C	C	C	C	C
65,454	polB	DNA polymerase II	R109R (CGT→CGC)	A	G	G	G	G	G
65,594	polB	DNA polymerase II	F63V (TTT→GTT)	A	C	C	C	C	C
65,785	polB/araD	DNA polymerase II/L‑ribulose‑5‑phosphate 4‑epimerase	intergenic (‑5/+70)	A	T	T	T	T	T
65,788	polB/araD	DNA polymerase II/L‑ribulose‑5‑phosphate 4‑epimerase	intergenic (‑8/+67)	A	T	T	T	T	T
65,811	polB/araD	DNA polymerase II/L‑ribulose‑5‑phosphate 4‑epimerase	intergenic (‑31/+44)	G	T	T	T	T	T
65,905	araD	L‑ribulose‑5‑phosphate 4‑epimerase	D216N (GAT→AAT)	C	T	T	T	T	T
66,024	araD	L‑ribulose‑5‑phosphate 4‑epimerase	coding (527/696 nt)		Δ1 bp	Δ1 bp	Δ1 bp	Δ1 bp	Δ1 bp
66,032	araD	L‑ribulose‑5‑phosphate 4‑epimerase	P173P (CCG→CCA)	C	T	T	T	T	T
66,041	araD	L‑ribulose‑5‑phosphate 4‑epimerase	S170S (TCC→TCT)	G	A	A	A	A	A
66,053	araD	L‑ribulose‑5‑phosphate 4‑epimerase	V166V (GTT→GTC)	A	G	G	G	G	G
66,092	araD	L‑ribulose‑5‑phosphate 4‑epimerase	F153F (TTT→TTC)	A	G	G	G	G	G
66,134	araD	L‑ribulose‑5‑phosphate 4‑epimerase	G139G (GGC→GGT)	G	A	A	A	A	A
66,263	araD	L‑ribulose‑5‑phosphate 4‑epimerase	T96T (ACG→ACA)	C	T	T	T	T	T
66,266	araD	L‑ribulose‑5‑phosphate 4‑epimerase	H95H (CAT→CAC)	A	G	G	G	G	G
66,287	araD	L‑ribulose‑5‑phosphate 4‑epimerase	P88P (CCC→CCG)	G	C	C	C	C	C
66,308	araD	L‑ribulose‑5‑phosphate 4‑epimerase	R81R (CGG→CGA)	C	T	T	T	T	T
66,323	araD	L‑ribulose‑5‑phosphate 4‑epimerase	D76D (GAC→GAT)	G	A	A	A	A	A
66,343	araD	L‑ribulose‑5‑phosphate 4‑epimerase	T70A (ACG→GCG)	T	C	C	C	C	C
66,403	araD	L‑ribulose‑5‑phosphate 4‑epimerase	V50I (GTC→ATC)	C	T	T	T	T	T
66,494	araD	L‑ribulose‑5‑phosphate 4‑epimerase	H19H (CAC→CAT)	G	A	A	A	A	A
66,518	araD	L‑ribulose‑5‑phosphate 4‑epimerase	E11E (GAA→GAG)	T	C	C	C	C	C
66,686	araD/araA	L‑ribulose‑5‑phosphate 4‑epimerase/L‑arabinose isomerase	intergenic (‑136/+149)	C	G	G	G	G	G
66,688	araD/araA	L‑ribulose‑5‑phosphate 4‑epimerase/L‑arabinose isomerase	intergenic (‑138/+147)	G	A	A	A	A	A
66,784	araD/araA	L‑ribulose‑5‑phosphate 4‑epimerase/L‑arabinose isomerase	intergenic (‑234/+51)	A	T	T	T	T	T
66,819	araD/araA	L‑ribulose‑5‑phosphate 4‑epimerase/L‑arabinose isomerase	intergenic (‑269/+16)	T	C	C	C	C	C

66,823	araD/araA	L-ribulose-5-phosphate 4-epimerase/L-arabinose isomerase	intergenic (-273/+12)	G	T	T	T	T	T
66,830	araD/araA	L-ribulose-5-phosphate 4-epimerase/L-arabinose isomerase	intergenic (-280/+5)	G	A	A	A	A	A
66,847	araA	L-arabinose isomerase	G497G (GGG→GGA)	C	T	T	T	T	T
66,898	araA	L-arabinose isomerase	T480T (ACA→ACC)	T	G	G	G	G	G
66,901	araA	L-arabinose isomerase	D479D (GAC→GAT)	G	A	A	A	A	A
66,946	araA	L-arabinose isomerase	Q464Q (CAA→CAG)	T	C	C	C	C	C
66,970	araA	L-arabinose isomerase	A456A (GCA→GCG)	T	C	C	C	C	C
67,111	araA	L-arabinose isomerase	C409C (TGC→TGT)	G	A	A	A	A	A
67,123	araA	L-arabinose isomerase	L405L (CTA→CTG)	T	C	C	C	C	C
67,152	araA	L-arabinose isomerase	L396L (TTG→CTG)	A	G	G	G	G	G
67,171	araA	L-arabinose isomerase	G389G (GGC→GGT)	G	A	A	A	A	A
67,195	araA	L-arabinose isomerase	R381R (CGC→CGA)	G	T	T	T	T	T
67,259	araA	L-arabinose isomerase	A360V (GCA→GTA)	G	A	A	A	A	A
67,264	araA	L-arabinose isomerase	I358I (ATC→ATT)	G	A	A	A	A	A
67,405	araA	L-arabinose isomerase	T311T (ACT→ACC)	A	G	G	G	G	G
67,471	araA	L-arabinose isomerase	P289P (CCT→CCA)	A	T	T	T	T	T
67,596	araA	L-arabinose isomerase	K248E (AAA→GAA)	T	C	C	C	C	C
67,612	araA	L-arabinose isomerase	A242A (GCC→GCA)	G	T	T	T	T	T
67,759	araA	L-arabinose isomerase	G193G (GGC→GGT)	G	A	A	A	A	A
67,798	araA	L-arabinose isomerase	R180R (CGA→CGT)	T	A	A	A	A	A
67,834	araA	L-arabinose isomerase	V168V (GTC→GTT)	G	A	A	A	A	A
71,579	yabI	inner membrane protein, SNARE_assoc family	L77M (CTG→ATG)	C			A		
72,799	thiQ	thiamin transporter subunit	N43N (AAT→AAC)	A	G	G	G	G	G
74,644	thiB	thiamin transporter subunit	N279K (AAC→AAA)	G				T	T
75,240	thiB	thiamin transporter subunit	G81W (GGG→TGG)	C			A		
75,560	thiB/sgrR	thiamin transporter subunit/transcriptional DNA-binding transcriptional activator of sgrS sRNA	intergenic (-80/+84)	G				T	T
77,776	setA	broad specificity sugar efflux system	G52G (GGC→GGA)	C	A	A	A	A	A
78,406	setA	broad specificity sugar efflux system	G262G (GGA→GGT)	A	T	T	T	T	T
78,520	setA	broad specificity sugar efflux system	F300L (TTC→TTA)	C	A	A	A	A	A
78,524	setA	broad specificity sugar efflux system	N302H (AAT→CAT)	A	C	C	C	C	C
78,533	setA	broad specificity sugar efflux system	M305L (ATG→CTG)	A	C	C	C	C	C
80,703	leuC	3-isopropylmalate dehydratase large subunit	P54P (CCG→CCT)	C				A	A
85,170	leuO	DNA-binding transcriptional activator	A268D (GCT→GAT)	C			A		
92,705	ftsI	transpeptidase involved in septal peptidoglycan synthesis (penicillin-binding protein 3)	V431V (GTC→GTA)	C				A	A
106,484	ftsZ/lpxC	GTP-binding tubulin-like cell division protein/UDP-3-O-acyl N-acetylglucosamine deacetylase	intergenic (+28/-73)	C		A			
128,139	lpd	lipoamide dehydrogenase, E3 component is part of three enzyme complexes	F76L (TTC→TTA)	C			A		
130,123	yacH	predicted protein	R380R (CGG→AGG)	G			T		

141,593	hpt	hypoxanthine phosphoribosyltransferase	E55* (GAA→TAA)	G			T		
151,742	yadL	predicted fimbrial-like adhesin protein	A164T (GCG→ACG)	C	T	T	T	T	T
151,836	yadL	predicted fimbrial-like adhesin protein	V132V (GTC→GTA)	G			T		
156,040	ecpD	predicted periplasmic pilin chaperone	P54P (CCG→CCT)	C			A		
164,403	hrpB	predicted ATP-dependent helicase	D767Y (GAT→TAT)	G		T			
177,200	yadS	inner membrane protein, UPF0126 family	P142H (CCC→CAC)	G			T		
177,207	yadS	inner membrane protein, UPF0126 family	E140* (GAA→TAA)	C	A	A	A	A	A
177,288	yadS	inner membrane protein, UPF0126 family	E113K (GAA→AAA)	C	T	T	T	T	T
194,855	dxr/ispU	1-deoxy-D-xylulose 5-phosphate reductoisomerase/undecaprenyl pyrophosphate synthase	intergenic (+138/-48)	C			A		
202,559	fabZ/lpxA	(3R)-hydroxymyristol acyl carrier protein dehydratase/UDP-N-acetylglucosamine acetyltransferase	intergenic (+3/-1)	C			A		
217,716	proS	prolyl-tRNA synthetase	A354S (GCG→TCG)	C			A		
225,750	alaV/rrlH	tRNA-Ala/23S ribosomal RNA of rrnH operon	intergenic (+175/-9)	G	A	A	A	A	A
229,552	dkgB	2,5-diketo-D-gluconate reductase B	S129F (TCC→TTC)	C	T	T	T	T	T
232,660	mltD	predicted membrane-bound lytic murein transglycosylase D	A432A (GCG→GCT)	C			A		
247,143	yafL	predicted lipoprotein and C40 family peptidase	G144G (GGC→GGA)	C			A		
249,848	lfhA	pseudogene, flagellar system protein, promoterless fragment; flagellar biosynthesis	pseudogene (223/1713 nt)	C			A		
250,330	lafU	pseudogene, lateral flagellar motor protein fragment	pseudogene (259/756 nt)	G			T		
252,236	yafN	antitoxin of the YafO-YafN toxin-antitoxin system	R78S (CGC→AGC)	C			A		
256,601	frsA	fermentation-respiration switch protein; PTS Enzyme IIA(Glc)-binding protein; pNP-butyrate esterase activity	R25R (CGC→CGA)	C			A		
258,145	crl	sigma factor-binding protein, stimulates RNA polymerase holoenzyme formation	coding (317-325/402 nt)		IS1 (+) +9 bp	IS1 (+) +9 bp	IS1 (+) +9 bp	IS1 (+) +9 bp	IS1 (+) +9 bp
264,533	ykfF	CP4-6 prophage; predicted protein	R79S (CGC→AGC)	G			T		
265,846	ykfL	pseudogene, CP4-6 putative prophage remnant;Phage or Prophage Related	pseudogene (153/222 nt)	C			A		
276,892	mmuM/afuC	CP4-6 prophage; S-methylmethionine:homocysteine methyltransferase/CP4-6 prophage; predicted ferric transporter subunit	intergenic (+21/+88)	G			T		
282,198	yagE	2-keto-3-deoxy gluconate (KDG) aldolase; CP4-6 prophage	G233R (GGG→AGG)	G	A	A	A	A	A
307,754	yagX	predicted aromatic compound dioxygenase	S268Y (TCC→TAC)	G			T		
313,005	ykgP	pseudogene, oxidoreductase family	pseudogene (25/90 nt)	G				T	T
316,175	ykgA	pseudogene, AraC family;putative regulator; Not classified; putative ARAC-type regulatory protein	pseudogene (219/684 nt)	G			T		
317,603	ykgI	predicted protein	S63S (TCG→TCT)	C				A	A
321,979	ykgF	predicted electron transport protein with ferridoxin-like domai	E140* (GAA→TAA)	G			T		
322,675	[ykgF]–[lacY]	[ykgF], ykgG, ykgH, betA, betB, betI, betT, yahA, yahB, yahC, yahD, yahE, yahF, yahG, yahI, yahJ, yahK, yahL, yahM, yahN, yahO, prpR, prpB, prpC, prpD, prpE, codB, codA, cynR, cynT, cynS, cynX, lacA, [lacY]	Δ38,752 bp		Δ38,752 bp	Δ38,752 bp	Δ38,752 bp	Δ38,752 bp	Δ38,752 bp
364,817	lacZ	beta-D-galactosidase	R238L (CGC→CTC)	C			A		
370,374	mhpB	2,3-dihydroxyphenylpropionate 1,2-dioxygenase	G292W (GGG→TGG)	G			T		
376,192	yaiL	predicted protein	A66E (GCG→GAG)	C			A		

378,408	frmA	alcohol dehydrogenase class III/glutathione-dependent formaldehyde dehydrogenase	Q130K (CAG→AAG)	G		T		T	T
386,065	tauB	taurine transporter subunit	R212L (CGG→CTG)	G			T		
394,510	ampH	penicillin-binding protein	F334L (TTC→TTA)	G			T		
396,909	sbmA	microcin B17 transporter	L349L (CTC→CTA)	C			A		
400,621	iraP	anti-RssB factor, RpoS stabilzer during Pi starvation; anti-adapter protein	L4L (CTC→CTA)	C			A		
402,161	phoA	bacterial alkaline phosphatase	Q397H (CAG→CAT)	G				T	T
414,905	sbcC	exonuclease, dsDNA, ATP-dependent	E25* (GAG→TAG)	C				A	A
419,549	brnQ	Branched-chain amino acid transport system 2 carrier protein; LIV-II transport system for Ile, Leu, and Val	A245A (GCG→GCT)	G			T		
435,231	thiL	thiamin-monophosphate kinase	R125L (CGT→CTT)	G			T		
439,625	ispA	geranyltranstransferase	T234K (ACG→AAG)	G			T		
445,056	yajR	predicted transporter	Q279K (CAA→AAA)	G				T	T
454,097	bolA/tig	stationary-phase morphogene, transcriptional repressor for mreB; also regulator for dacA, dacC, and ampC/peptidyl-prolyl cis/trans isomerase (trigger factor)	intergenic (+84/-260)	C			A		
457,393	clpX	ATPase and specificity subunit of ClpX-ClpP ATP-dependent serine protease	G248G (GGC→GGA)	C			A		
459,229	lon	DNA-binding ATP-dependent protease La	T373I (ACC→ATC)	C	T	T	T	T	T
465,142	ybaE	predicted transporter subunit: periplasmic-binding component of ABC superfamily	G465G (GGC→GGA)	G			T		
478,024	ylaC	inner membrane protein, DUF1449 family	R151H (CGC→CAC)	C	T	T	T	T	T
486,482	kefA	fused conserved protein	A241A (GCG→GCA)	G	A	A	A	A	A
490,134	ybaN	inner membrane protein, DUF454 family	G10V (GGC→GTC)	G			T		
493,916	recR	gap repair protein	E96D (GAG→GAT)	G			T		
502,389	ybaL	predicted transporter with NAD(P)-binding Rossmann-fold domain	A25D (GCC→GAC)	G			T		
503,946	fsr/ushA	predicted fosmidomycin efflux system/bifunctional UDP-sugar hydrolase/5'-nucleotidase	intergenic (-26/-192)	G			T		
504,911	ushA	bifunctional UDP-sugar hydrolase/5'-nucleotidase	C258* (TGC→TGA)	C			A		
507,040	ybaP	GumN family protein	R89S (CGC→AGC)	G				T	T
520,848	ybbP	predicted ABC transporter permease	L403L (CTG→CTT)	G				T	T
526,365	rhsD	rhsD element protein	A1294V (GCG→GTG)	C	T	T	T	T	T
529,626	selU	tRNA 2-selenouridine synthase, selenophosphate-dependent	W275* (TGG→TGA)	C	T	T	T	T	T
534,419	gcl	glyoxylate carboligase	A427D (GCT→GAT)	C			A		
540,005	ybbY	predicted uracil/xanthine transporter	G73R (GGG→AGG)	G	A	A	A	A	A
544,560	allD	ureidoglycolate dehydrogenase	T343K (ACG→AAG)	G				T	T
547,694	ylbE	predicted protein, C-ter fragment (pseudogene)	pseudogene (139/252 nt)	A	G	G	G	G	G
547,835	ylbE	predicted protein, C-ter fragment (pseudogene)	pseudogene (1005/1008 nt)		=+G	=+G	=+G	=+G	=+G
548,146	ylbE	predicted protein, C-ter fragment (pseudogene)	pseudogene (694/1008 nt)	C			A		
549,922	ybcF	predicted carbamate kinase	M87I (ATG→ATT)	G			T		
558,360	sfmC	pilin chaperone, periplasmic	R55L (CGC→CTC)	G				T	T

565,637	peaD	DLP12 prophage; predicted replication protein fragment (pseudogene);Phage or Prophage Related	pseudogene (39/312 nt)	A	R	R	R	R	R
565,788	peaD	DLP12 prophage; predicted replication protein fragment (pseudogene);Phage or Prophage Related	pseudogene (190/312 nt)	A	R	R	R	R	R
565,793	peaD	DLP12 prophage; predicted replication protein fragment (pseudogene);Phage or Prophage Related	pseudogene (195/312 nt)	A	M	M	M	M	M
572,854	rusA	DLP12 prophage; endonuclease RUS	F87L (TTC→TTA)	C			A		
577,083	rrrD	DLP12 prophage; predicted lysozyme	A83D (GCC→GAC)	C			A		
577,828	borD	DLP12 prophage; predicted lipoprotein	K97Q (AAA→CAA)	T	G	G	G	G	G
578,034	borD	DLP12 prophage; predicted lipoprotein	Q28?	T	K	K	K	K	K
578,680	ybcV	DLP12 prophage; predicted protein	I46I (ATT→ATC)	A	R	R	R	R	R
578,725	ybcV	DLP12 prophage; predicted protein	N31N (AAT→AAC)	A	R	R	R	R	R
578,940	ybcV/ybcW	DLP12 prophage; predicted protein/DLP12 prophage; predicted protein	intergenic (-123/-163)	A	C	C	C	C	C
579,525	ylcI	hypothetical protein	R48R (AGG→AGA)	C	Y	Y	Y	Y	Y
584,252	ompT	DLP12 prophage; outer membrane protease VII (outer membrane protein 3b)	G202V (GGC→GTC)	C			A		
584,298	ompT	DLP12 prophage; outer membrane protease VII (outer membrane protein 3b)	E187* (GAA→TAA)	C			A		
588,230	nfrA	bacteriophage N4 receptor, outer membrane subunit	A650T (GCA→ACA)	C	T	T	T	T	T
588,893	nfrA	bacteriophage N4 receptor, outer membrane subunit	H429Y (CAT→TAT)	G	A	A	A	A	A
590,113	nfrA	bacteriophage N4 receptor, outer membrane subunit	S22N (AGT→AAT)	C	T	T	T	T	T
594,596	cusR	DNA-binding response regulator in two-component regulatory system with CusS	G24V (GGT→GTT)	C		A			
597,658	cusB	copper/silver efflux system, membrane fusion protein	T319T (ACC→ACT)	C	T	T	T	T	T
598,796	cusA	copper/silver efflux system, membrane component	G287V (GGG→GTG)	G		T			
612,893	fes	enterobactin/ferric enterobactin esterase	H286N (CAT→AAT)	C				A	A
613,963	entF	enterobactin synthase multienzyme complex component, ATP-dependent	A195E (GCA→GAA)	C			A		
615,475	entF	enterobactin synthase multienzyme complex component, ATP-dependent	A699E (GCA→GAA)	C				A	A
628,980	entH/cstA	thioesterase required for efficient enterobactin production/carbon starvation protein	intergenic (+44/-137)	G			T		
629,611	cstA	carbon starvation protein	V165V (GTG→GTT)	G			T		
634,275	ybdM	conserved protein	E109* (GAA→TAA)	C			A		
660,973	lipB	octanoyltransferase; octanoyl-[ACP]:protein N-octanoyltransferase	A177S (GCT→TCT)	C	A	A	A	A	A
676,198	ybeR	predicted protein	A89S (GCT→TCT)	G				T	T
683,351	rihA	ribonucleoside hydrolase 1	P95P (CCG→CCT)	C			A		
683,578	rihA	ribonucleoside hydrolase 1	V20F (GTT→TTT)	C		A		A	A
686,782	gltI	glutamate, aspartate binding protein, periplasmic; part of GltJKLI ABC transporter	V63V (GTG→GTT)	C			A		
690,305	ybeX	predicteed ion transport	E235* (GAA→TAA)	C				A	A
692,279	ybeZ	predicted protein with nucleoside triphosphate hydrolase domain	A108E (GCG→GAG)	G			T		
695,094	ubiF	2-octaprenyl-3-methyl-6-methoxy-1,4-benzoquinol oxygenase	L257L (CTG→CTT)	G			T		
695,999	glnW	tRNA-Gln	noncoding (55/75 nt)	G			T		
708,110	chiP	chitoporin, uptake of chitosugars	A185D (GCT→GAT)	C			A		

720,582	kdpE	DNA-binding response regulator in two-component regulatory system with KdpD	P125P (CCC→CCA)	G			T		
743,504	ybgK	predicted enzyme subunit	V13V (GTG→GTT)	G			T		
746,360	abrB	regulator of aidB expression; inner membrane protein	M211I (ATG→ATT)	C			A		
753,364	gltA	citrate synthase	R110S (CGT→AGT)	G				T	T
757,648	sdhB/sucA	succinate dehydrogenase, FeS subunit/2-oxoglutarate decarboxylase, thiamin-requiring	intergenic (+20/-281)	G			T		
759,998	sucA	2-oxoglutarate decarboxylase, thiamin-requiring	A690A (GCC→GCA)	C				A	A
772,087	cydA	cytochrome d terminal oxidase, subunit I	G469G (GGC→GGA)	C			A		
773,524	ybgT	conserved protein	D36Y (GAC→TAC)	G			T		
793,774	modE	DNA-binding transcriptional repressor for the molybdenum transport operon modABC	G32C (GGT→TGT)	C			A		
799,107	ybhD	predicted DNA-binding transcriptional regulator	G231V (GGA→GTA)	C			A		
804,666	ybhJ	predicted hydratase	A647A (GCG→GCT)	G				T	T
807,953	bioA	7,8-diaminopelargonic acid synthase, PLP-dependent	P176P (CCC→CCA)	G			T		
810,464	bioF	8-amino-7-oxononanoate synthase	E287D (GAG→GAT)	G			T		
823,120	ybhP	conserved protein, endo/exonuclease/phosphatase family PFAM PF03372	H201N (CAC→AAC)	G			T		
824,990	ybhR	predicted transporter subunit: membrane component of ABC superfamily	T114T (ACC→ACA)	G			T		
827,186	ybhF	fused predicted transporter subunits of ABC superfamily: ATP-binding components	G340V (GGG→GTG)	C			A		
838,743	fiu	catecholate siderophore receptor Fiu	A671E (GCA→GAA)	G			T		
844,744	ybiO/glnQ	predicted mechanosensitive channel/glutamine transporter subunit	intergenic (-41/+220)	G			T		
850,915	ybiP	predicted hydrolase, inner membrane	Q302H (CAG→CAT)	C			A		
851,452	ybiP	predicted hydrolase, inner membrane	G123G (GGC→GGA)	G			T		
853,369	ybiR	predicted transporter	A167D (GCT→GAT)	C			A		
853,761	ybiR	predicted transporter	P298T (CCG→ACG)	C			A		
857,112	ybiU	predicted protein	F391L (TTC→TTA)	G				T	T
858,330	ybiU/ybiV	predicted protein/sugar phosphatase; preference for fructose-1-P, ribose-5-P and glucose-6-P	intergenic (-46/+106)	G	A	A	A	A	A
860,425	ybiW	predicted pyruvate formate lyase	A469S (GCG→TCG)	C			A		
870,390	gsiC	glutathione transporter, permease component, ABC superfamily	H67Q (CAC→CAA)	C			A		
872,277	yliE	predicted cyclic-di-GMP phosphodiesterase, inner membrane protein	L26I (CTT→ATT)	C				A	A
875,315	yliF	predicted diguanylate cyclase	R253I (AGA→ATA)	G			T		
877,536	bssR	repressor of biofilm formation by indole transport regulation	A22A (GCC→GCA)	C			A		
885,261	ybjI	FMN and erythrose-4-P phosphatase	Q32K (CAA→AAA)	G			T		
901,102	artQ	arginine transporter subunit	R124R (CGG→CGT)	C				A	A
910,233	poxB	pyruvate dehydrogenase (pyruvate oxidase), thiamin-dependent, FAD-binding	E14* (GAA→TAA)	C			A		
917,614	ybjX	conserved protein	Q244K (CAG→AAG)	G			T		
945,381	ycaD	predicted MFS-type transporter	S96S (AGC→AGT)	C	T	T	T	T	T
949,208	ycaK	conserved protein	W106* (TGG→TGA)	G	A	A	A	A	A

949,689	pflA	pyruvate formate lyase activating enzyme 1	L205L (CTG→CTT)	C			A		
953,136	focA	formate channel	G185V (GGC→GTC)	C			A		
954,219	ycaO	ribosomal protein S12 methylthiotransferase accessory factor	G546V (GGC→GTC)	C				A	
975,650	mukB	chromosome condensin MukBEF, ATPase and DNA-binding subunit	G34G (GGC→GGA)	C				A	A
978,718	mukB	chromosome condensin MukBEF, ATPase and DNA-binding subunit	A1057E (GCG→GAG)	C				A	A
981,132	ycbB	murein L,D-transpeptidase	A288D (GCT→GAT)	C				A	A
984,696	aspC	aspartate aminotransferase, PLP-dependent	Q79H (CAG→CAT)	C			A		
986,288	ompF/asnS	outer membrane porin 1a (Ia;b;F)/asparaginyl tRNA synthetase	intergenic (-83/+520)	G				T	T
992,020	pepN	aminopeptidase N	P726S (CCT→TCT)	C	T	T	T	T	T
992,492	pepN/ssuB	aminopeptidase N/alkanesulfonate transporter subunit	intergenic (+35/+8)	C		A			
1,000,474	elfC	predicted outer membrane usher protein	Q679P (CAA→CCA)	A	C	C	C	C	C
1,005,501	zapC	FtsZ-associated protein	P109P (CCG→CCT)	G		T			
1,006,451	ycbX	predicted 2Fe-2S cluster-containing protein; 6-N-hydroxylaminopurine resistance protein	P125T (CCA→ACA)	G			T		
1,009,305	uup	fused predicted transporter subunits of ABC superfamily: ATP-binding components	A40E (GCA→GAA)	C			A		
1,023,139	yccF	inner membrane protein, DUF307 family	R145S (CGT→AGT)	G			T		
1,030,767	yccA/serT	HflBKC-binding inner membrane protein, UPF0005 family/tRNA-Ser	intergenic (-126/+81)	C		A			
1,033,488	hyaB	hydrogenase 1, large subunit	D338N (GAT→AAT)	G	A	A	A	A	A
1,047,820	gfcC	conserved protein	G32V (GGC→GTC)	C			A		
1,050,035	insB/cspH	IS1 transposase B/stress protein, member of the CspA-family	intergenic (+282/+151)	C			A		
1,051,923	yccM	predicted 4Fe-4S membrane protein	A221A (GCC→GCA)	G			T		
1,054,822	torS	hybrid sensory histidine kinase in two-component regulatory system with TorR	D194Y (GAT→TAT)	C			A		
1,062,393	cbpA	curved DNA-binding protein, DnaJ homologue that functions as a co-chaperone of DnaK	P202P (CCG→CCT)	C			A		
1,071,426	rutB	ureidoacrylate amidohydrolase	L221I (CTT→ATT)	G			T		
1,076,556	putA	fused DNA-binding transcriptional regulator/proline dehydrogenase/pyrroline-5-carboxylate dehydrogenase	G517V (GGC→GTC)	C			A		
1,081,656	efeO	inactive ferrous ion transporter EfeUOB	A64E (GCG→GAG)	C			A		
1,086,784	pgaC	predicted glycosyl transferase	A96T (GCC→ACC)	C	T	T	T	T	T
1,102,807	csgD/csgB	DNA-binding transcriptional activator for csgBA/curlin nucleator protein, minor subunit in curli complex	intergenic (-388/-367)	C			A		
1,110,016	opgG	osmoregulated periplasmic glucan (OPG) biosynthesis periplasmic protein	E487* (GAA→TAA)	G			T		
1,114,034	mdtG	predicted drug efflux system	T227N (ACT→AAT)	G			T		
1,148,195	fabH	3-oxoacyl-[acyl-carrier-protein] synthase III	Q72* (CAG→TAG)	C	T	T	T	T	T
1,156,595	ycfH	predicted DNAse	R199L (CGG→CTG)	G			T		
1,166,206	ndh	respiratory NADH dehydrogenase 2/cupric reductase	P300Q (CCG→CAG)	C	A				
1,166,661	ndh/ycfJ	respiratory NADH dehydrogenase 2/cupric reductase/predicted protein	intergenic (+49/-161)	G	A	A	A	A	A
1,167,163	ycfJ	predicted protein	G114G (GGC→GGA)	C			A		
1,167,680	ycfQ	repressor for bhsA(ycfR)	L126I (CTC→ATC)	G		T			
1,169,059	ycfS	L,D-transpeptidase linking Lpp to murein	L180P (CTG→CCG)	A	G	G	G	G	G
1,173,009	mfd	transcription-repair coupling factor	S60I (AGC→ATC)	C			A		

1,189,203	phoP	DNA‑binding response regulator in two‑component regulatory system with PhoQ	T156T (ACT→ACC)	A	G	G	G	G	G
1,192,955	mnmA	tRNA (5‑methylaminomethyl‑2‑thiouridylate)‑methyltransferase	G14G (GGC→GGA)	G			T		
1,204,278	aaaE	pseudogene, phage terminase protein A family, e14 prophage;Phage or Prophage Related	pseudogene (652/1134 nt)	G			T		
1,215,793	ariR	connector protein for RcsB regulation of biofilm and acid‑resistance	L68I (CTC→ATC)	C			A		
1,216,499	ymgC/ycgG	predicted protein/predicted cyclic‑di‑GMP phosphodiesterase	intergenic (+280/‑52)	G			T		
1,220,265	ycgH	Probable pseudogene; putative ATP‑binding component of a transport system	pseudogene (1442/2648 nt)	G			T		
1,253,601	ycgV	predicted adhesin	S525R (AGC→AGA)	G			T		
1,260,746	prs	phosphoribosylpyrophosphate synthase	S118Y (TCC→TAC)	G			T		
1,263,147	hemA	glutamyl tRNA reductase	R71S (CGC→AGC)	C			A		
1,268,319	kdsA/ldrA	3‑deoxy‑D‑manno‑octulosonate 8‑phosphate synthase/toxic polypeptide, small	intergenic (+77/+72)	C			A		
1,269,344	ldrB/ldrC	toxic polypeptide, small/toxic polypeptide, small	intergenic (‑311/+117)	G			T		
1,275,269	narX	sensory histidine kinase in two‑component regulatory system with NarL	Q525K (CAA→AAA)	G			T		
1,283,826	narH	nitrate reductase 1, beta (Fe‑S) subunit	A334S (GCA→TCA)	G			T		
1,286,263	narI/tpr	nitrate reductase 1, gamma (cytochrome b(NR)) subunit/protamine‑like protein	intergenic (+514/+47)	G	A	A	A	A	A
1,290,123	rssB	response regulator binding RpoS to initiate proteolysis by ClpXP; required for the PcnB‑degradosome interaction during stationary phase	coding (659‑667/1014 nt)		IS1 (–) +9 bp	IS1 (–) +9 bp	IS1 (–) +9 bp	IS1 (–) +9 bp	IS1 (–) +9 bp
1,293,719	insZ	pseudogene, transposase homolog	pseudogene (497/567 nt)	G			T		
1,300,016	oppA	oligopeptide transporter subunit	N271Y (AAT→TAT)	A	T	T	T	T	T
1,303,173	oppD	oligopeptide transporter subunit	M132I (ATG→ATT)	G			T		
1,304,760	oppF	oligopeptide transporter subunit	S325A (TCC→GCC)	T	G	G	G	G	G
1,307,548	kch	voltage‑gated potassium channel	C249F (TGC→TTC)	C			A		
1,311,909	yciC/ompW	inner membrane protein, UPF0259 family/outer membrane protein W	intergenic (‑222/‑135)	C			A		
1,326,283	btuR	cob(I)alamin adenolsyltransferase/cobinamide ATP‑dependent adenolsyltransferase	V33V (GTC→GTA)	G			T		
1,334,698	acnA	aconitate hydratase 1	G282R (GGG→AGG)	G	A	A	A	A	A
1,335,184	acnA	aconitate hydratase 1	L444M (CTG→ATG)	C	A	A	A	A	A
1,335,418	acnA	aconitate hydratase 1	S522G (AGC→GGC)	A	G	G	G	G	G
1,337,339	ribA/pgpB	GTP cyclohydrolase II/phosphatidylglycerophosphatase B	intergenic (‑155/‑15)	C	T	T	T	T	T
1,339,839	yciM/pyrF	TPR‑repeats‑containing protein/orotidine‑5'‑phosphate decarboxylase	intergenic (+88/‑106)	C	A	A	A	A	A
1,351,274	sapD	antimicrobial peptide transport ABC system ATP‑binding protein	Q127K (CAG→AAG)	G			T		
1,353,276	sapB	antimicrobial peptide transport ABC transporter permease	V73V (GTC→GTA)	G			T		
1,355,161	sapA/ymjA	antimicrobial peptide transport ABC transporter periplasmic binding protein/predicted protein	intergenic (‑27/+286)	G		T			
1,356,883	puuP	putrescine importer	Y110C (TAT→TGT)	T	C	C	C	C	C
1,358,097	puuA	gamma‑Glu‑putrescine synthase	S279I (AGC→ATC)	C			A		
1,359,543	puuD	gamma‑Glu‑GABA hydrolase	E134* (GAA→TAA)	G				T	T

1,365,974	pspF/pspA	DNA-binding transcriptional activator/regulatory protein for phage-shock-protein operon	intergenic (-38/-129)	C				A	A
1,374,420	ycjR	predicted enzyme	D121E (GAC→GAA)	C			A		
1,421,268	rzpR	pseudogene, Rac prophage; predicted defective peptidase;Phage or Prophage Related; putative Rac prophage endopeptidase	pseudogene (44/444 nt)	G	S	S	S	S	S
1,424,487	ynaA	Rac prophage; pseudogene, tail protein homology;Phage or Prophage Related; putative alpha helix protein	pseudogene (10/933 nt)	C				A	A
1,426,949	lomR	pseudogene, Rac prophage lom homolog;Phage or Prophage Related; interrupted by IS5 and N-ter deletion	pseudogene (60/190 nt)	C	Y	Y	Y	Y	Y
1,427,912	stfR	Rac prophage; predicted tail fiber protein	N280N (AAC→AAT)	C	N	N	N	N	N
1,429,603	stfR	Rac prophage; predicted tail fiber protein	R844L (CGC→CTC)	G			T		
1,432,802	ynaE/ttcC	cold shock protein, function unknown, Rac prophage/pseudogene, prophage Rac integration site ttcA duplication;Phage or Prophage Related	intergenic (-554/+180)	G				T	T
1,434,256	ompN	outer membrane pore protein N, non-specific	S221Y (TCT→TAT)	G			T		
1,437,193	ydbK	fused predicted pyruvate-flavodoxin oxidoreductase: conserved protein/conserved protein/FeS binding protein	R539L (CGC→CTC)	C			A		
1,449,764	paaZ	fused oxepin-CoA hydrolase/3-oxo-5,6-dehydrosuberyl-CoA semialdehyde dehydrogenase	L635M (CTG→ATG)	G			T		
1,459,509	paaJ	3-oxoadipyl-CoA/3-oxo-5,6-dehydrosuberyl-CoA thiolase	S198* (TCA→TAA)	C			A		
1,460,280	paaK	phenylacetyl-CoA ligase	F44L (TTC→TTA)	C			A		
1,461,968	paaX	DNA-binding transcriptional repressor of phenylacetic acid degradation paa operon, phenylacetyl-CoA inducer	G136C (GGC→TGC)	G	T	T	T	T	T
1,469,654	ydbA	pseudogene, autotransporter homolog; interrupted by IS2 and IS30	pseudogene (2384/3497 nt)	G	T	T	T	T	T
1,482,417	hrpA	predicted ATP-dependent helicase	L445M (CTG→ATG)	C			A		
1,485,952	ydcF	conserved SAM-binding protein, DUF218 superfamily	D232Y (GAT→TAT)	G		T			
1,486,039	ydcF	conserved SAM-binding protein, DUF218 superfamily	E261K (GAA→AAA)	G	A	A	A	A	A
1,499,152	tehA	potassium-tellurite ethidium and proflavin transporter	R186S (CGT→AGT)	C				A	A
1,509,958	ydcS	polyhydroxybutyrate (PHB) synthase, ABC transporter periplasmic binding protein homolog	S94Y (TCC→TAC)	C			A		
1,510,438	ydcS	polyhydroxybutyrate (PHB) synthase, ABC transporter periplasmic binding protein homolog	S254Y (TCC→TAC)	C			A		
1,520,230	[yncD]-[adhP]	[yncD], yncE, ansP, yncG, yncH, rhsE, ydcD, yncI, yncI, ydcC, pptA, yddH, nhoA, yddE, narV, narW, narY, narZ, narU, yddJ, yddK, yddL, yddG, fdnG, fdnH, fdnI, yddM, [adhP]	Δ31,130 bp				Δ31,130 bp		
1,547,701	fdnG	formate dehydrogenase-N, alpha subunit, nitrate-inducible	S759S (TCC→TCA)	C				A	A
1,552,469	maeA	malate dehydrogenase, (decarboxylating, NAD-requiring) (malic enzyme)	P409T (CCG→ACG)	G			T		
1,553,001	maeA	malate dehydrogenase, (decarboxylating, NAD-requiring) (malic enzyme)	I231I (ATC→ATA)	G			T		
1,555,159	ddpF	D,D-dipeptide permease system, ATP-binding component	L302I (CTC→ATC)	G			T		
1,558,660	ddpB	D-ala-D-ala transporter subunit	P98P (CCG→CCT)	C			A		
1,575,946	yddA	fused predicted multidrug transporter subunits of ABC superfamily: membrane component/ATP-binding component	A474V (GCT→GTT)	G	A	A	A	A	A
1,581,040	ydeO	transcriptional activator for mdtEF	G224G (GGC→GGA)	G				T	T

1,585,488	ydeQ	predicted fimbrial-like adhesin protein	L91I (CTA→ATA)	G			T		
1,598,515	lsrR	lsr operon transcriptional repressor	A251S (GCA→TCA)	C	A	A	A	A	A
1,613,782	yneJ/yneK	predicted DNA-binding transcriptional regulator/predicted protein	intergenic (+73/-5)	C		A			
1,622,109	ydeH/ydeI	diguanylate cyclase, required for pgaD induction/conserved protein	intergenic (-235/+20)	G			T		
1,629,174	ydfJ	pseudogene, MFS transporter family; interrupted by Qin prophage;Phage or Prophage Related; putative transport protein	pseudogene (1136/1284 nt)	G	A	A	A	A	A
1,632,373	tfaQ	Qin prophage; predicted tail fibre assembly protein	T179T (ACG→ACA)	C	T	T	T	T	T
1,632,394	tfaQ	Qin prophage; predicted tail fibre assembly protein	V172V (GTA→GTG)	T	C	C	C	C	C
1,633,392	stfQ	Qin prophage; predicted side tail fibre assembly protein	K160K (AAA→AAG)	T	Y	Y	Y	Y	Y
1,641,703	ydfU	Qin prophage; predicted protein	L209Q (CTG→CAG)	A	T	T	T	T	T
1,646,448	dicA/ydfA	Qin prophage; predicted regulator for DicB/Qin prophage; predicted protein	intergenic (+83/-84)	C			A		
1,647,454	ydfC/dicB	conserved protein, Qin prophage/Qin prophage; cell division inhibition protein	intergenic (+389/-175)				IS2 (+) +5 bp		
1,650,355	intQ	pseudogene, Qin prophage; predicted defective integrase;Phage or Prophage Related	pseudogene (781/1158 nt)	T	C	C	C	C	C
1,661,010	ynfF/ynfG	S- and N-oxide reductase, A subunit, periplasmic/oxidoreductase, Fe-S subunit	intergenic (+7/-4)	G				T	T
1,678,367	ydgI	predicted arginine/ornithine antiporter transporter	E263* (GAA→TAA)	G			T		
1,696,407	malI	transcriptional repressor of Mal regulon	G266G (GGG→GGT)	C		A			
1,710,095	nth	DNA glycosylase and apyrimidinic (AP) lyase (endonuclease III)	G183G (GGG→GGT)	G				T	T
1,715,995	pdxH	pyridoxine 5'-phosphate oxidase	L13M (CTG→ATG)	G			T		
1,729,286	lhr	predicted ATP-dependent helicase	G726C (GGC→TGC)	G			T		
1,743,876	ydhQ	conserved protein	L92L (CTC→CTA)	G			T		
1,744,951	ydhR	predicted monooxygenase	L76L (CTC→CTT)	C	T	T	T	T	T
1,761,878	sufB	component of SufBCD complex	E52D (GAG→GAT)	C			A		
1,781,858	fadK	short chain acyl-CoA synthetase, anaerobic	A268A (GCG→GCT)	G				T	T
1,791,519	btuD	vitamin B12 transporter subunit : ATP-binding component of ABC superfamily	E22K (GAG→AAG)	C	T	T	T	T	T
1,791,673	btuE	glutathione peroxidase	R154K (AGG→AAG)	C	T	T	T	T	T
1,804,996	pfkB	6-phosphofructokinase II	Q201H (CAG→CAT)	G			T		
1,812,290	katE	catalase HPII, heme d-containing	A134S (GCT→TCT)	G			T		
1,818,442	chbC	N,N'-diacetylchitobiose-specific enzyme IIC component of PTS	W266L (TGG→TTG)	C			A		
1,821,478	nadE/cho	NAD synthetase, NH3/glutamine-dependent/endonuclease of nucleotide excision repair	intergenic (+169/-61)	C	T	T	T	T	T
1,825,937	astB	succinylarginine dihydrolase	P116L (CCA→CTA)	G	A	A	A	A	A
1,827,911	astA	arginine succinyltransferase	A293A (GCC→GCT)	G	A	A	A	A	A
1,834,099	ynjB	conserved protein	M1I (ATG→ATT)	G			T		
1,837,144	ynjD	predicted transporter subunit: ATP-binding component of ABC superfamily	G125V (GGA→GTA)	G				T	T
1,840,515	gdhA	glutamate dehydrogenase, NADP-specific	Q41* (CAA→TAA)	C	T	T	T	T	T
1,840,943	gdhA	glutamate dehydrogenase, NADP-specific	M183I (ATG→ATT)	G			T		
1,855,879	ydjJ	predicted oxidoreductase, Zn-dependent and NAD(P)-binding	A327T (GCA→ACA)	C	T	T	T	T	T

1,884,280	yeaX	predicted oxidoreductase	D138Y (GAC→TAC)	G			T		
1,892,863	pabB	aminodeoxychorismate synthase, subunit I	L12P (CTC→CCC)	T	C	C	C	C	C
1,894,221	nudL	predicted NUDIX hydrolase	D10Y (GAT→TAT)	G				T	T
1,899,770	yoaE/manX	fused predicted membrane protein/conserved protein/fused mannose-specific PTS enzymes: IIA component/IIB component	intergenic (-161/-302)	C		A			
1,905,533	yobF	predicted protein	A28D (GCC→GAC)	G		T			
1,905,630	yobF/yebO	predicted protein/predicted inner membrane protein	intergenic (-15/+655)	C				A	A
1,911,654	prc	carboxy-terminal protease for penicillin-binding protein 3	G396V (GGC→GTC)	C			A		
1,918,448	rsmF	16S rRNA m(5)C1407 methyltransferase, SAM-dependent	P68T (CCG→ACG)	C			A		
1,920,472	pphA	serine/threonine-specific protein phosphatase 1	G174G (GGG→GGT)	C			A		
1,943,326	ruvB	ATP-dependent DNA helicase, component of RuvABC resolvasome	A19T (GCA→ACA)	C	T	T	T	T	T
1,952,717	torZ	trimethylamine N-oxide reductase system III, catalytic subunit	P772H (CCC→CAC)	G			T		
1,962,476	flhA	predicted flagellar export pore protein	G200V (GGG→GTG)	C		A		A	A
1,964,340	flhB/cheZ	flagellin export apparatus, substrate specificity protein/chemotaxis regulator, protein phosphatase for CheY	intergenic (-125/+77)	G			T		
1,976,527	insB–insA	insB, insA	Δ776 bp			Δ776 bp	Δ776 bp	Δ776 bp	Δ776 bp
1,984,587	araF/ftnB	L-arabinose transporter subunit/ferritin B, probable ferrous iron reservoir	intergenic (-435/-362)	C			A		
1,985,574	yecJ	predicted protein	A70E (GCG→GAG)	G			T		
1,987,811	tyrP	tyrosine transporter	T36M (ACG→ATG)	C	T	T	T	T	T
1,994,968	sdiA/yecC	quorum-sensing transcriptional activator/predicted transporter subunit: ATP-binding component of ABC superfamily	intergenic (-112/+118)	C			A		
2,011,336	fliF	flagellar basal-body MS-ring and collar protein	A28A (GCC→GCA)	C			A		
2,014,014	fliH	negative regulator of FliI ATPase activity	E41D (GAG→GAT)	G			T		
2,015,853	fliI	flagellum-specific ATP synthase	P426T (CCG→ACG)	C				A	A
2,018,287	fliM	flagellar motor switching and energizing component	E59D (GAG→GAT)	G			T		
2,018,294	fliM	flagellar motor switching and energizing component	A62S (GCC→TCC)	G			T		
2,018,642	fliM	flagellar motor switching and energizing component	E178* (GAG→TAG)	G			T		
2,026,335	yodC	predicted protein	V20V (GTC→GTA)	G			T		
2,032,106	yedS	pseudogene, outer membrane protein homology; putative outer membrane protein	pseudogene (632/663 nt)	C	A	A	A	A	A
2,038,457	yedY	membrane-anchored, periplasmic TMAO, DMSO reductase	A319D (GCC→GAC)	C	A	A	A	A	A
2,040,195	zinT/yodB	zinc and cadmium binding protein, periplasmic/cytochrome b561 homolog	intergenic (+146/-197)	G	A	A	A	A	A
2,042,666	asnT/yeeJ	tRNA-Asn/probable adhesin	intergenic (+18/-296)	T	C	C	C	C	C
2,044,464	yeeJ	probable adhesin	T501T (ACC→ACA)	C	A	A	A	A	A
2,045,621	yeeJ	probable adhesin	A887E (GCG→GAG)	C			A		
2,056,771	yeeO	predicted multdrug exporter, MATE family	A304E (GCG→GAG)	G			T		
2,061,527	cobT	nicotinate-nucleotide--dimethylbenzimidazole phosphoribosyltransferase	A322E (GCA→GAA)	G			T		
2,062,174	cobT	nicotinate-nucleotide--dimethylbenzimidazole phosphoribosyltransferase	A106A (GCG→GCT)	C			A		
2,077,721	yeeA	inner membrane protein, FUSC family	E299* (GAA→TAA)	C			A		
2,079,471	dacD	D-alanyl-D-alanine carboxypeptidase (penicillin-binding protein 6b)	T367T (ACC→ACT)	G	A	A	A	A	A

2,081,006	sbcB	exonuclease I	A76E (GCG→GAG)	C			A		
2,086,031	yeeY	predicted DNA-binding transcriptional regulator	A84A (GCG→GCT)	C				A	A
2,097,139	ugd	UDP-glucose 6-dehydrogenase	A167S (GCA→TCA)	C			A		
2,098,163	gnd	6-phosphogluconate dehydrogenase, decarboxylating	A377D (GCT→GAT)	G			T		
2,106,762	rfbX	predicted polisoprenol-linked O-antigen transporter	R283S (CGT→AGT)	G			T		
2,109,283	rfbD	dTDP-4-dehydrorhamnose reductase subunit, NAD(P)-binding, of dTDP-L-rhamnose synthase	coding (718/900 nt)		Δ1 bp	Δ1 bp	Δ1 bp	Δ1 bp	Δ1 bp
2,114,499	wcaL	predicted glycosyl transferase	A218E (GCG→GAG)	G				T	T
2,115,847	wcaK	Colanic acid biosynthesis protein	S194R (AGC→AGA)	G			T		
2,118,177	wzxC	predicted colanic acid exporter	S2R (AGC→AGA)	G				T	T
2,120,418	cpsG	phosphomannomutase	L196I (CTC→ATC)	G			T		
2,125,156	fcl	bifunctional GDP-fucose synthetase: GDP-4-dehydro-6-deoxy-D-mannose epimerase/ GDP-4-dehydro-6-L-deoxygalactose reductase	R20M (AGG→ATG)	C			A		
2,127,073	wcaE	predicted glycosyl transferase	S201Y (TCT→TAT)	G			T		
2,132,867	wzc	protein-tyrosine kinase	A270A (GCC→GCA)	G			T		
2,133,716	wzb	protein-tyrosine phosphatase	R136L (CGC→CTC)	C				A	A
2,134,329	wza	lipoprotein required for capsular polysaccharide translocation through the outer membrane	K313N (AAG→AAT)	C			A		
2,134,601	wza	lipoprotein required for capsular polysaccharide translocation through the outer membrane	R223S (CGC→AGC)	G			T		
2,136,192	yegH	inner membrane protein	I89I (ATC→ATA)	C			A		
2,143,435	yegE	predicted diguanylate cyclase, GGDEF domain signaling protein	L716M (CTG→ATG)	C		A		A	A
2,157,494	mdtC	multidrug efflux system, subunit C	P362H (CCC→CAC)	C		A			
2,158,854	mdtC	multidrug efflux system, subunit C	S815S (TCG→TCT)	G				T	T
2,171,470	gatC	galactitol-specific enzyme IIC component of PTS	Q277Q (CAG→CAA)	C	T	T	T	T	T
2,175,082	gatY	D-tagatose 1,6-bisphosphate aldolase 2, catalytic subunit	G49* (GGA→TGA)	C			A		
2,192,552	metG	methionyl-tRNA synthetase	M77I (ATG→ATT)	G			T		
2,212,278	yehU	predicted sensory kinase in two-component system with YehT, inner membrane protein	R130L (CGC→CTC)	C		A		A	A
2,230,034	cdd	cytidine/deoxycytidine deaminase	L57I (CTT→ATT)	C		A			
2,233,307	preA	Dihydropyrimidine dehydrogenase, NADH-dependent, subunit B	S7S (TCG→TCT)	G			T		
2,238,647	mglB/galS	methyl-galactoside transporter subunit/DNA-binding transcriptional repressor	intergenic (-277/+3)	C		A	A	A	A
2,243,436	cirA	catecholate siderophore receptor CirA	S452R (AGC→AGA)	G				T	T
2,246,327	lysP	lysine transporter	M76I (ATG→ATT)	C			A		
2,257,048	psuK	pseudouridine kinase	G91* (GGA→TGA)	C			A		
2,260,331	fruK	fructose-1-phosphate kinase	F19L (TTC→TTA)	G			T		
2,262,269	setB	lactose/glucose efflux system	L129I (CTT→ATT)	C			A		
2,279,314	yejH	predicted ATP-dependent DNA or RNA helicase	L221M (CTG→ATG)	C				A	A
2,286,602	yejO	pseudogene, autotransporter outer membrane homology;putative transport; Not classified; putative ATP-binding component of a transport system	pseudogene (335/2525 nt)	G			T		

2,288,744	narP	DNA-binding response regulator in two-component regulatory system with NarQ or NarX	R75S (CGC→AGC)	C			A		
2,297,116	napH	ferredoxin-type protein essential for electron transfer from ubiquinol to periplasmic nitrate reductase (NapAB)	P162H (CCC→CAC)	G			T		
2,297,420	napH	ferredoxin-type protein essential for electron transfer from ubiquinol to periplasmic nitrate reductase (NapAB)	D61Y (GAC→TAC)	C			A		
2,300,614	napA	nitrate reductase, periplasmic, large subunit	G54G (GGC→GGA)	G			T		
2,308,939	apbE	predicted thiamine biosynthesis lipoprotein	R206R (CGC→CGA)	G			T		
2,356,966	rhmT	predicted L-rhamnonate transporter	A403A (GCG→GCT)	C			A		
2,358,386	rhmD	L-rhamnonate dehydratase	P351T (CCG→ACG)	G			T		
2,359,627	rhmR	predicted DNA-binding transcriptional regulator for the rhm operon	A203S (GCT→TCT)	C			A		
2,370,320	arnT	4-amino-4-deoxy-L-arabinose transferase	A464E (GCA→GAA)	C				A	A
2,371,198	arnF	undecaprenyl phosphate-alpha-L-ara4N exporter; flippase ArnEF subunit	P95P (CCC→CCA)	C			A		
2,387,435	yfbP	TPR-like repeats-containing protein	A100S (GCA→TCA)	G				T	T
2,390,056	nuoM	NADH:ubiquinone oxidoreductase, membrane subunit M	L336F (TTG→TTT)	C			A		
2,394,195	nuoI	NADH:ubiquinone oxidoreductase, chain I	R93L (CGC→CTC)	C			A		
2,400,025	nuoE	NADH:ubiquinone oxidoreductase, chain E	A17E (GCA→GAA)	G				T	T
2,400,033	nuoE	NADH:ubiquinone oxidoreductase, chain E	L14L (CTG→CTT)	C			A		
2,410,048	yfbT	sugar phosphatas	E22* (GAA→TAA)	C			A		
2,423,134	hisM	histidine/lysine/arginine/ornithine transporter subunit	A41E (GCG→GAG)	G			T		
2,431,842	accD	acetyl-CoA carboxylase, beta (carboxyltransferase) subunit	R36H (CGC→CAC)	C	T	T	T	T	T
2,433,869	usg	predicted semialdehyde dehydrogenase	G268V (GGC→GTC)	C			A		
2,448,233	yfcP	predicted fimbrial-like adhesin protein	G127V (GGA→GTA)	C			A		
2,448,360	yfcP	predicted fimbrial-like adhesin protein	G85W (GGG→TGG)	C			A		
2,466,320	gtrB	CPS-53 (KpLE1) prophage; bactoprenol glucosyl transferase	coding (85-87/921 nt)		Δ3 :: IS3 (+) +3 bp	Δ3 :: IS3 (+) +3 bp	Δ3 :: IS3 (+) +3 bp	Δ3 :: IS3 (+) +3 bp	Δ3 :: IS3 (+) +3 bp
2,468,170	gtrS	serotype-specific glucosyl transferase, CPS-53 (KpLE1) prophage	E340* (GAA→TAA)	G			T		
2,473,250	yfdR	CPS-53 (KpLE1) prophage; conserved protein	P82Q (CCA→CAA)	C			A		
2,480,217	emrK	EmrKY-TolC multidrug resistance efflux pump, membrane fusion protein component	S382* (TCG→TAG)	G			T		
2,483,932	evgS	hybrid sensory histidine kinase in two-component regulatory system with EvgA	R513S (CGC→AGC)	C			A		
2,485,055	evgS	hybrid sensory histidine kinase in two-component regulatory system with EvgA	R887L (CGC→CTC)	G			T		
2,485,582	evgS	hybrid sensory histidine kinase in two-component regulatory system with EvgA	L1063M (CTG→ATG)	C			A		
2,489,374	oxc	oxalyl CoA decarboxylase, ThDP-dependent	S200* (TCA→TAA)	G			T		
2,491,959	yfdX	predicted protein	G156C (GGT→TGT)	C			A		
2,519,807	xapR	DNA-binding transcriptional activator for for xapAB	P231P (CCG→CCT)	C			A		
2,533,069	ptsI	PEP-protein phosphotransferase of PTS system (enzyme I)	A328S (GCG→TCG)	G			T		
2,533,641	ptsI	PEP-protein phosphotransferase of PTS system (enzyme I)	M518I (ATG→ATT)	G			T		
2,539,668	cysW	sulfate/thiosulfate ABC transporter subunit	A12S (GCG→TCG)	C			A		
2,546,830	yfeW	weak penicillin binding protein PBP4B, predicted periplasmic esterase	G236V (GGC→GTC)	G			T		

2,550,399	amiA	N‑acetylmuramoyl‑l‑alanine amidase I	T9K (ACA→AAA)	C	A	A	A	A	A
2,556,721	intZ–[eutA]	intZ, yffL, yffM, yffN, yffO, yffP, yffQ, yffR, yffS, [eutA]	Δ6,790 bp		Δ6,790 bp	Δ6,790 bp	Δ6,790 bp	Δ6,790 bp	Δ6,790 bp
2,569,189	eutE	aldehyde oxidoreductase, ethanolamine utilization protein	P195P (CCG→CCT)	C			A		
2,585,134	narQ	sensory histidine kinase in two‑component regulatory system with NarP (NarL)	A461V (GCA→GTA)	C	T	T	T	T	T
2,586,145	acrD	aminoglycoside/multidrug efflux system	A177S (GCC→TCC)	G			T		
2,586,782	acrD	aminoglycoside/multidrug efflux system	S389I (AGC→ATC)	G			T		
2,588,870	ypfM	hypothetical protein	G7W (GGG→TGG)	C			A		
2,594,277	ypfJ	conserved protein	Q161H (CAG→CAT)	C				A	A
2,595,695	purC/bamC	phosphoribosylaminoimidazole‑succinocarboxamide synthetase/lipoprotein required for OM biogenesis, in BamABCDE complex	intergenic (‑55/+158)	G			T		
2,612,001	focB	predicted formate transporter	A16S (GCG→TCG)	G			T		
2,623,267	ppx	exopolyphosphatase	R44L (CGG→CTG)	G				T	T
2,626,193	yfgF	cyclic‑di‑GMP phosphodiesterase, anaerobic	W256C (TGG→TGT)	C			A		
2,628,032	yfgH	outer membrane lipoprotein	G73G (GGC→GGA)	C				A	A
2,634,144	der	GTPase; multicopy suppressor of ftsJ	A412D (GCC→GAC)	G				T	T
2,634,581	der	GTPase; multicopy suppressor of ftsJ	I266I (ATC→ATA)	G			T		
2,645,187	pbpC	penicillin‑binding protein PBP1C murein transglycosylase; inactive transpeptidase domain	W54L (TGG→TTG)	C			A		
2,667,948	hcaE	3‑phenylpropionate dioxygenase, large (alpha) subunit	R299S (CGC→AGC)	C			A		
2,671,229	hcaD	phenylpropionate dioxygenase, ferredoxin reductase subunit	A387A (GCG→GCT)	G			T		
2,672,558	yphB	conserved protein	V51V (GTG→GTT)	C			A		
2,679,188	yphG	conserved protein	A527E (GCG→GAG)	G				T	T
2,679,727	yphG	conserved protein	L347F (TTG→TTT)	C				A	A
2,697,209	yfhH	predicted DNA‑binding transcriptional regulator	S143S (TCG→TCT)	G				T	T
2,714,045	eamB/yfiD	cysteine and O‑acetylserine exporter/autonomous glycyl radical cofactor	intergenic (+13/+43)	G	A	A	A	A	A
2,718,021	yfiQ	inhibiting acetyltransferase for acetyl‑CoA synthetase	A16E (GCG→GAG)	C			A		
2,723,871	kgtP/rrfG	alpha‑ketoglutarate transporter/5S ribosomal RNA of rrnG operon	intergenic (‑103/+220)	C				A	A
2,738,773	aroF	3‑deoxy‑D‑arabino‑heptulosonate‑7‑phosphate synthase, tyrosine‑repressible	L134M (CTG→ATG)	G			T		
2,745,535	ffh	Signal Recognition Particle (SRP) component with 4.5S RNA (ffs)	L95M (CTG→ATG)	G				T	T
2,751,041	recN	recombination and repair protein	A409S (GCC→TCC)	G			T		
2,753,092	smpB	trans‑translation protein	R59S (CGT→AGT)	C			A		
2,757,189	yfjI	CP4‑57 prophage; predicted protein	P61P (CCG→CCT)	G			T		
2,765,964	yfjP	CP4‑57 prophage; predicted GTP‑binding protein	R78L (CGC→CTC)	G				T	T
2,766,418	yfjP	CP4‑57 prophage; predicted GTP‑binding protein	P229P (CCC→CCA)	C			A		
2,774,009	yfjX	CP4‑57 prophage; predicted antirestriction protein	Q23H (CAG→CAT)	G			T		
2,779,548	ypjA	adhesin‑like autotransporter	G401C (GGC→TGC)	C			A		
2,792,281	gabP	gamma‑aminobutyrate transporter	Q3K (CAA→AAA)	C			A		
2,792,552	gabP	gamma‑aminobutyrate transporter	T93N (ACC→AAC)	C				A	A

2,799,482	nrdE	ribonucleoside-diphosphate reductase 2, alpha subunit	R38L (CGC→CTC)	G			T		
2,808,674	ygaH	probable L-valine exporter, norvaline resistance	L103L (CTC→CTA)	C	A	A	A	A	A
2,812,814	luxS/gshA	S-ribosylhomocysteine lyase/glutamate-cysteine ligase	intergenic (-59/+91)	C			A		
2,817,088	csrA	pleiotropic regulatory protein for carbon source metabolism	G27G (GGC→GGA)	G			T		
2,817,811	alaS	alanyl-tRNA synthetase	Q741H (CAG→CAT)	C		A			
2,828,497	gutQ	D-arabinose 5-phosphate isomerase	A221A (GCG→GCT)	G					T
2,833,970	hypF	carbamoyl phosphate phosphatase and maturation protein for [NiFe] hydrogenases	G493V (GGC→GTC)	C			A		
2,844,556	hycD	hydrogenase 3, membrane subunit	G293G (GGG→GGA)	C	T	T	T	T	T
2,850,406	hypD	protein required for maturation of hydrogenases	V83V (GTG→GTT)	G			T		
2,851,042	hypD	protein required for maturation of hydrogenases	L295L (CTG→CTT)	G			T		
2,851,582	hypE	carbamoyl dehydratase, hydrogenases 1,2,3 maturation protein	G103* (GGA→TGA)	G			T		
2,852,520	fhlA	DNA-binding transcriptional activator	A54E (GCG→GAG)	C			A		
2,852,585	fhlA	DNA-binding transcriptional activator	E76* (GAA→TAA)	G			T		
2,859,916	ygbJ	predicted dehydrogenase, with NAD(P)-binding Rossmann-fold domain	P155P (CCC→CCA)	C			A		
2,864,449	ygbN	predicted transporter	G443* (GGA→TGA)	G			T		
2,865,477	rpoS	RNA polymerase, sigma S (sigma 38) factor	Q33* (CAG→TAG)	G	A	A	A	A	A
2,870,603	ftsB	cell division protein	P80P (CCG→CCT)	C			A		
2,875,059	iap	aminopeptidase in alkaline phosphatase isozyme conversion	E153* (GAA→TAA)	G			T		
2,878,665	casD	CRISP RNA (crRNA) containing Cascade antiviral complex protein	A136S (GCA→TCA)	C				A	A
2,902,810	queE	7-carboxy-7-deazaguanine synthase; queosine biosynthesis	R211S (CGT→AGT)	G			T		
2,903,199	queE	7-carboxy-7-deazaguanine synthase; queosine biosynthesis	R81L (CGC→CTC)	C				A	A
2,905,784	eno	enolase	L60L (CTG→CTT)	C			A		
2,905,855	eno	enolase	A37S (GCT→TCT)	C			A		
2,919,938	gudP	predicted D-glucarate transporter	S62Y (TCT→TAT)	G			T		
2,924,794	ygdH	conserved protein, UPF0717 family	G155G (GGC→GGA)	C	A	A	A	A	A
2,929,251	ygdG	Ssb-binding protein, misidentified as ExoIX	R59S (CGT→AGT)	C			A		
2,930,660	fucO	L-1,2-propanediol oxidoreductase	P126T (CCG→ACG)	G			T		
2,930,716	fucO	L-1,2-propanediol oxidoreductase	G107V (GGC→GTC)	C				A	A
2,938,418	rlmM	23S rRNA C2498 ribose 2'-O-methyltransferase, SAM-dependent	A283E (GCG→GAG)	G			T		
2,941,561	csdA	cysteine sulfinate desulfinase	A68V (GCA→GTA)	C	T	T	T	T	T
2,950,373	recD	exonuclease V (RecBCD complex), alpha chain	L37L (CTC→CTA)	G			T		
2,951,089	recB	exonuclease V (RecBCD complex), beta subunit	L979L (CTC→CTA)	G				T	T
2,953,305	recB	exonuclease V (RecBCD complex), beta subunit	G241C (GGT→TGT)	C			A		
2,954,649	ptrA	protease III	S753I (AGC→ATC)	C			A		
2,961,613	ppdB	conserved protein	T42T (ACC→ACA)	G			T		
2,964,432	ptsP	fused PTS enzyme: PEP-protein phosphotransferase (enzyme I)/GAF domain containing protein	P675P (CCC→CCA)	G			T		
2,987,970	yqeK	predicted protein	T138I (ACC→ATC)	G	A	A	A	A	A

3,000,873	xdhB	xanthine dehydrogenase, FAD-binding subunit	L80I (CTA→ATA)	C			A		
3,008,980	hyuA	D-stereospecific phenylhydantoinase	A311T (GCA→ACA)	G	A	A	A	A	A
3,017,009	ygfK	predicted oxidoreductase, Fe-S subunit	F976F (TTC→TTT)	C	T	T	T	T	T
3,017,295	ssnA	predicted chlorohydrolase/aminohydrolase	T38K (ACG→AAG)	C			A		
3,021,812	xdhD	probable hypoxanthine oxidase, molybdopterin-binding/Fe-S binding	T825T (ACG→ACT)	G			T		
3,032,016	lysS	lysine tRNA synthetase, constitutive	P394Q (CCG→CAG)	G			T		
3,040,740	yqfA	inner membrane protein, hemolysin III family HylIII	T144I (ACC→ATC)	G	A	A	A	A	A
3,050,582	ubiH	2-octaprenyl-6-methoxyphenol hydroxylase, FAD/NAD(P)-binding	G320V (GGA→GTA)	C			A		
3,052,597	pepP	proline aminopeptidase P II	W89L (TGG→TTG)	C			A		
3,057,185	rpiA	ribose 5-phosphate isomerase, constitutive	A55S (GCT→TCT)	C			A		
3,066,559	argO	arginine transporter	A91E (GCA→GAA)	G			T		
3,074,642	yggP	predicted dehydrogenase	A279A (GCG→GCT)	C			A		
3,075,106	yggP	predicted dehydrogenase	A125S (GCC→TCC)	C			A		
3,101,930	mutY	adenine DNA glycosylase	L299* (TTA→TAA)	T	A	A	A	A	A
3,105,606	speC	ornithine decarboxylase, constitutive	E524D (GAG→GAT)	C			A		
3,107,699	yqgA	predicted inner membrane protein, DUF554 family	S42* (TCG→TAG)	C			A		
3,119,457	glcA/glcB	glycolate transporter/malate synthase G	intergenic (-156/+199)	G			T		
3,126,054	glcD/glcC	glycolate oxidase subunit, FAD-linked/DNA-binding transcriptional dual regulator, glycolate-binding	intergenic (-11/-240)	C		A		A	A
3,126,288	glcD/glcC	glycolate oxidase subunit, FAD-linked/DNA-binding transcriptional dual regulator, glycolate-binding	intergenic (-245/-6)	C			A		
3,135,831	gss	fused glutathionylspermidine amidase/glutathionylspermidine synthetase	V238V (GTG→GTA)	C	T	T	T	T	T
3,137,154	yghU	predicted S-transferase	D136N (GAT→AAT)	G	A	A	A	A	A
3,138,454	hybE	hydrogenase 2-specific chaperone	G123D (GGC→GAC)	C		T			
3,143,768	hybO	hydrogenase 2, small subunit	G172G (GGC→GGT)	G		A			
3,146,370	gpr	L-glyceraldehyde 3-phosphate reductase	S151Y (TCT→TAT)	C			A		
3,150,516	metC	cystathionine beta-lyase, PLP-dependent	A87S (GCG→TCG)	G	T	T	T	T	T
3,153,409	yqhD	aldehyde reductase, NADPH-dependent	R11R (CGC→CGA)	C			A		
3,159,268	ygiQ/ftsP	conserved protein/septal ring component that protects the divisome from stress; multicopy suppressor of ftsI(Ts)	intergenic (-100/+11)	G			T		
3,161,663	plsC/parC	1-acyl-sn-glycerol-3-phosphate acyltransferase/DNA topoisomerase IV, subunit A	intergenic (-160/+74)	G			T		
3,170,530	ygiZ/mdaB	inner membrane protein/NADPH quinone reductase	intergenic (-297/-22)	C				A	A
3,174,757	cpdA	3',5' cAMP phosphodiesterase	L33L (CTG→CTT)	C			A		
3,188,458	yqiH	predicted periplasmic pilin chaperone	G186W (GGG→TGG)	G			T		
3,189,104	yqiI	conserved protein	P151T (CCG→ACG)	C			A		
3,197,472	glnE	fused deadenylyltransferase/adenylyltransferase for glutamine synthetase	D64E (GAC→GAA)	G				T	T
3,200,357	cca	fused tRNA nucleotidyl transferase/2'3'-cyclic phosphodiesterase/2'nucleotidase and phosphatase	G149C (GGT→TGT)	G			T		

3,200,635	cca	fused tRNA nucleotidyl transferase/2'3'-cyclic phosphodiesterase/2'nucleotidase and phosphatase	M241I (ATG→ATT)	G			T		
3,204,708	ttdA	L-tartrate dehydratase, alpha subunit	G75V (GGG→GTG)	G			T		
3,205,571	ttdB	L-tartrate dehydratase, beta subunit	coding (179/606 nt)		Δ1 bp	Δ1 bp	Δ1 bp	Δ1 bp	Δ1 bp
3,209,075	rpsU/dnaG	30S ribosomal subunit protein S21/DNA primase	intergenic (+57/-54)	C		A		A	A
3,211,144	rpoD	RNA polymerase, sigma 70 (sigma D) factor	E26* (GAG→TAG)	G	T	T	T	T	T
3,212,445	rpoD	RNA polymerase, sigma 70 (sigma D) factor	T459T (ACC→ACA)	C			A		
3,213,772	yqjH	predicted siderophore interacting protein	A248S (GCG→TCG)	C			A		
3,218,619	patA	putrescine:2-oxoglutaric acid aminotransferase, PLP-dependent	D368E (GAC→GAA)	C			A		
3,221,439	ebgA	cryptic beta-D-galactosidase, alpha subunit	A262D (GCC→GAC)	C			A		
3,224,705	ygjI	predicted transporter	G150G (GGG→GGT)	G			T		
3,231,977	higA	antitoxinof the HigB-HigA toxin-antitoxin system	A64S (GCG→TCG)	C			A		
3,233,576	rlmG	23S rRNA mG1835 methyltransferase, SAM-dependent	G108C (GGT→TGT)	C			A		
3,242,383	uxaC	uronate isomerase	I127I (ATC→ATA)	G		T			
3,249,539	yqjG	predicted S-transferase	A165E (GCG→GAG)	C			A		
3,250,723	yhaH/yhaI	inner membrane protein, DUF805 family/inner membrane protein, DUF805 family	intergenic (+32/-210)	C			A		
3,252,661	yhaK	redox-sensitive bicupin	S107R (AGC→AGA)	C			A		
3,280,110	agaS	tagatose-6-phosphate ketose/aldose isomerase	R38L (CGT→CTT)	G			T		
3,283,291	agaC	N-acetylgalactosamine-specific enzyme IIC component of PTS	G195G (GGC→GGA)	C			A		
3,286,499	yraI	predicted periplasmic pilin chaperone	V130I (GTA→ATA)	G	A	A	A	A	A
3,290,629	rsmI	16S rRNA C1402 ribose 2'-O-methyltransferase, SAM-dependent	P243P (CCC→CCA)	G			T		
3,295,539	yraQ	predicted permease	D208Y (GAC→TAC)	C			A		
3,298,172	yhbQ	conserved protein, GIY-YIG nuclease superfamily	R62L (CGG→CTG)	G			T		
3,302,127	yhbW	predicted enzyme	A220T (GCG→ACG)	G	A	A	A	A	A
3,308,880	pnp	polynucleotide phosphorylase/polyadenylase	P104Q (CCG→CAG)	G	T	T	T	T	T
3,311,127	rbfA	30s ribosome binding factor	R25L (CGT→CTT)	C			A		
3,327,765	dacB	D-alanyl-D-alanine carboxypeptidase	D261Y (GAT→TAT)	G	T	T	T	T	T
3,348,578	elbB/arcB	isoprenoid biosynthesis protein with amidotransferase-like domain/aerobic respiration control sensor histidine protein kinase, cognate to two-component response regulators ArcA and RssB	intergenic (-97/+126)		IS1 (–) +8 bp	IS1 (–) +8 bp	IS1 (–) +8 bp	IS1 (–) +8 bp	IS1 (–) +8 bp
3,371,879	nanR	DNA-binding transcriptional repressor of the nan operon, induced by sialic acid	L211L (CTG→CTT)	C		A			
3,386,063	aaeB	p-hydroxybenzoic acid efflux system component	T50P (ACG→CCG)	T	G	G	G	G	G
3,386,727	aaeA	p-hydroxybenzoic acid efflux system component	A141E (GCG→GAG)	G				T	T
3,388,167	aaeR	transcriptional regulator for aaeXAB operon	S209* (TCG→TAG)	C			A		
3,389,673	tldD	predicted peptidase	L126L (CTG→CTT)	C			A		
3,392,264	yhdP	conserved membrane protein, predicted transporter	G673C (GGC→TGC)	C			A		
3,406,737	panF	pantothenate:sodium symporter	S370* (TCG→TAG)	C			A		
3,428,010	yrdB	conserved protein	coding (34-36/258 nt)		IS3 (–) +3 bp	IS3 (–) +3 bp	IS3 (–) +3 bp	IS3 (–) +3 bp	IS3 (–) +3 bp

3,434,165	rsmB	16S rRNA m(5)C967 methyltransferase, S-adenosyl-L-methionine-dependent	E313* (GAG→TAG)	G			T		
3,439,772	rpsK	30S ribosomal subunit protein S11	P117T (CCT→ACT)	G			T		
3,448,216	rplV	50S ribosomal subunit protein L22	A14S (GCT→TCT)	C			A		
3,451,465	rpsJ/gspB	30S ribosomal subunit protein S10/part of gsp divergon involved in type II protein secretion	intergenic (-173/+65)	C			A		
3,452,331	gspA	general secretory pathway component, cryptic	V364F (GTT→TTT)	C				A	A
3,458,635	gspF	general secretory pathway component, cryptic	R266L (CGC→CTC)	G			T		
3,459,201	gspG	pseudopilin, cryptic, general secretion pathway	E53* (GAA→TAA)	G			T		
3,466,288	chiA	periplasmic endochitinase	P530T (CCA→ACA)	G			T		
3,467,468	chiA	periplasmic endochitinase	G136G (GGC→GGT)	G	A	A	A	A	A
3,471,641	rpsG	30S ribosomal subunit protein S7	R155S (CGT→AGT)	G			T		
3,478,649	kefG	potassium-efflux system ancillary protein for KefB, glutathione-regulated	L179M (CTG→ATG)	G			T		
3,482,407	yheU	conserved protein	S56S (TCG→TCT)	G			T		
3,499,177	frlA	predicted fructoselysine transporter	G416C (GGC→TGC)	G			T		
3,501,507	frlD	fructoselysine 6-kinase	E107* (GAG→TAG)	G				T	T
3,524,973	yrfF	inner membrane protein	S161R (AGC→AGA)	C			A		
3,534,615	ompR/greB	DNA-binding response regulator in two-component regulatory system with EnvZ/transcript cleavage factor	intergenic (-9/-219)	C	T	T	T	T	T
3,535,227	greB	transcript cleavage factor	E132* (GAA→TAA)	G			T		
3,543,101	gntX	protein required for the utilization of DNA as a carbon source	T66T (ACG→ACT)	G			T		
3,543,949	nfuA	Fe/S biogenesis protein; possible scaffold/chaperone for damaged Fe/S proteins	A102S (GCA→TCA)	G				T	T
3,550,006	malP	maltodextrin phosphorylase	R164S (CGC→AGC)	G			T		
3,551,264	malT	DNA-binding transcriptional activator for the mal regulon and maltotriose-ATP-binding protein	A53E (GCG→GAG)	C		A		A	A
3,558,465	glpR	DNA-binding transcriptional repressor	G55A (GGT→GCT)	C	G	G	G	G	G
3,575,445	gntK	gluconate kinase 2	L57L (CTG→CTT)	C			A		
3,580,564	yhhZ	conserved protein	A227S (GCA→TCA)	G		T			
3,583,221	ggt	gamma-glutamyltranspeptidase	Q542H (CAG→CAT)	C			A		
3,583,599	ggt	gamma-glutamyltranspeptidase	T416T (ACG→ACT)	C			A		
3,590,672	ugpB/livF	glycerol-3-phosphate transporter subunit/leucine/isoleucine/valine transporter subunit	intergenic (-324/+75)	G				T	T
3,593,746	livH	leucine/isoleucine/valine transporter subunit	Y227* (TAC→TAA)	G				T	T
3,594,303	livH	leucine/isoleucine/valine transporter subunit	A42S (GCC→TCC)	C				A	A
3,596,474	yhhK/livJ	pantothenate synthesis protein, predicted acetyltransferase/leucine/isoleucine/valine transporter subunit	intergenic (+84/+104)	C				A	A
3,601,447	ftsY	Signal Recognition Particle (SRP) receptor	E274* (GAG→TAG)	C			A		
3,601,930	ftsY	Signal Recognition Particle (SRP) receptor	P113S (CCG→TCG)	G	A	A	A	A	A
3,613,396	nikB	nickel transporter subunit	P45S (CCG→TCG)	C	T	T	T	T	T
3,627,276	rbbA	fused ribosome-associated ATPase: ATP-binding protein/ATP-binding protein/predicted membrane protein	L96M (CTG→ATG)	G				T	T

3,628,180	yhiI	predicted membrane fusion protein (MFP) of efflux pump	S149Y (TCT→TAT)	G			T		
3,644,819	gor	glutathione oxidoreductase	L166F (TTG→TTT)	G			T		
3,656,633	gadE	DNA‑binding transcriptional activator	A82D (GCT→GAT)	C				A	A
3,666,768	yhjA	predicted cytochrome C peroxidase	L148L (CTG→CTT)	C			A		
3,678,684	yhjJ	predicted zinc‑dependent peptidase	S427I (AGC→ATC)	C			A		
3,679,880	yhjJ	predicted zinc‑dependent peptidase	Q28H (CAG→CAT)	C				A	A
3,687,331	bcsZ	endo‑1,4‑D‑glucanase	R318R (CGC→CGA)	G			T		
3,695,908	bcsE	cellulose production protein	L476F (TTG→TTT)	G			T		
3,702,686	dppC	dipeptide/heme transporter	V33V (GTC→GTA)	G				T	T
3,705,218	dppA	dipeptide transporter	E171* (GAA→TAA)	C	A	A	A	A	A
3,705,906	dppA/proK	dipeptide transporter/tRNA‑Pro	intergenic (‑178/+733)	G	A	A	A	A	A
3,705,970	dppA/proK	dipeptide transporter/tRNA‑Pro	intergenic (‑242/+669)	C	A	A	A	A	A
3,717,308	yiaF/yiaG	conserved protein/predicted transcriptional regulator, HTH_CROC1 family	intergenic (‑241/‑193)	A	T	T	T	T	T
3,722,603	glyQ	glycine tRNA synthetase, alpha subunit	R247C (CGT→TGT)	G	A	A	A	A	A
3,727,282	xylB	xylulokinase	P38Q (CCA→CAA)	G			T		
3,747,784	yiaR	predicted L‑xylulose 5‑phosphate 3‑epimerase	W177L (TGG→TTG)	G			T		
3,754,429	aldB	aldehyde dehydrogenase B	P36T (CCT→ACT)	G				T	T
3,755,847	yiaY	predicted Fe‑containing alcohol dehydrogenase, Pfam00465 family	A2T (GCA→ACA)	C	T	T	T	T	T
3,767,371	yibV	hypothetical protein, no homologs	pseudogene (4/439 nt)	G	A	A	A	A	A
3,767,528	yibV	hypothetical protein, no homologs	pseudogene (161/439 nt)	C	T	T	T	T	T
3,768,454	yibH	predicted protein	V317I (GTC→ATC)	C	T	T	T	T	T
3,769,123	yibH	predicted protein	L94I (CTC→ATC)	G			T		
3,772,522	mtlD	mannitol‑1‑phosphate dehydrogenase, NAD‑dependent	Q26K (CAA→AAA)	C			A		
3,780,587	cysE/gpsA	serine acetyltransferase/glycerol‑3‑phosphate dehydrogenase (NAD+)	intergenic (‑2/+78)	G			T		
3,781,668	gpsA	glycerol‑3‑phosphate dehydrogenase (NAD+)	A6D (GCT→GAT)	G			T		
3,786,113	envC	activator of AmiB,C murein hydrolases, septal ring factor	G418V (GGA→GTA)	G			T		
3,796,121	rfaL	O‑antigen ligase	G384V (GGG→GTG)	G			T		
3,800,559	rfaI	UDP‑D‑galactose:(glucosyl)lipopolysaccharide‑alpha‑1,3‑D‑galactosyltransferase	A175S (GCG→TCG)	C				A	A
3,801,960	rfaB	UDP‑D‑galactose:(glucosyl)lipopolysaccharide‑1, 6‑D‑galactosyltransferase	L67L (TTG→TTA)	C	T	T	T	T	T
3,809,009	mutM	formamidopyrimidine/5‑formyluracil/ 5‑hydroxymethyluracil DNA glycosylase	A56D (GCT→GAT)	G			T		
3,813,906	rph	defective ribonuclease PH	coding (667/687 nt)		+C	+C	+C	+C	+C
3,814,496	rph	defective ribonuclease PH	G26V (GGC→GTC)	C				A	A
3,817,515	ligB	DNA ligase, NAD(+)‑dependent	P560H (CCT→CAT)	G			T		
3,828,821	yicH	conserved protein	T114T (ACC→ACA)	C			A		
3,829,828	yicH	conserved protein	A450E (GCG→GAG)	C			A		
3,838,096	nlpA/yicS	cytoplasmic membrane lipoprotein‑28/predicted periplasmic protein	intergenic (‑80/‑142)	G			T		

3,842,569	ade	cryptic adenine deaminase	G195W (GGG→TGG)	G	T	T	T	T	T
3,864,459	yidE/ibpB	predicted transporter/heat shock chaperone	intergenic (‑163/+33)	C	T	T	T	T	T
3,871,957	dgoK	2‑oxo‑3‑deoxygalactonate kinase	E181K (GAA→AAA)	C	T	T	T	T	T
3,872,058	dgoK	2‑oxo‑3‑deoxygalactonate kinase	A147D (GCC→GAC)	G		T		T	T
3,876,253	gyrB	DNA gyrase, subunit B	V630V (GTG→GTA)	C	T	T	T	T	T
3,879,413	dnaN	DNA polymerase III, beta subunit	S311N (AGC→AAC)	C	T	T	T	T	T
3,886,841	tnaA	tryptophanase/L‑cysteine desulfhydrase, PLP‑dependent	A30E (GCA→GAA)	C				A	A
3,888,220	tnaA/tnaB	tryptophanase/L‑cysteine desulfhydrase, PLP‑dependent/tryptophan transporter of low affinity	intergenic (+52/‑39)	C	T	T	T	T	T
3,906,864	pstA	phosphate transporter subunit	A200E (GCG→GAG)	G			T		
3,909,913	glmS	L‑glutamine:D‑fructose‑6‑phosphate aminotransferase	G593G (GGC→GGA)	G			T		
3,911,667	glmS	L‑glutamine:D‑fructose‑6‑phosphate aminotransferase	A9S (GCG→TCG)	C				A	A
3,920,965	atpI/rsmG	ATP synthase, membrane‑bound accessory factor/16S rRNA m(7)G527 methyltransferase, SAM‑dependent; glucose‑inhibited cell‑division protein	intergenic (‑502/+115)	C	T	T	T	T	T
3,922,044	mnmG	5‑methylaminomethyl‑2‑thiouridine modification at tRNA U34	A538E (GCG→GAG)	G			T		
3,923,664	mnmG/mioC	5‑methylaminomethyl‑2‑thiouridine modification at tRNA U34/FMN‑binding protein MioC	intergenic (‑8/+371)	G				T	T
3,930,842	kup	potassium transporter	H502Y (CAT→TAT)	C	T	T	T	T	T
3,934,079	rbsC	D‑ribose transporter subunit	A257S (GCT→TCT)	G			T		
3,935,547	rbsK	ribokinase	L77L (CTC→CTT)	C	T	T	T	T	T
3,936,130	rbsK	ribokinase	P272T (CCA→ACA)	C			A		
3,937,537	hsrA	predicted multidrug or homocysteine efflux system	A367T (GCT→ACT)	C	T	T	T	T	T
3,953,165	ilvD	dihydroxyacid dehydratase	G555G (GGC→GGT)	C	T	T	T	T	T
3,957,957	ppiC/yifN	peptidyl‑prolyl cis‑trans isomerase C (rotamase C)/conserved protein (pseudogene)	intergenic (‑121/+78)	C	T	T	T	T	T
3,964,701	rho	transcription termination factor	R88S (CGT→AGT)	C				A	A
3,965,478	rho	transcription termination factor	R347S (CGT→AGT)	C			A		
3,972,717	rffC	TDP‑fucosamine acetyltransferase	P76P (CCC→CCA)	C	A	A	A	A	A
3,975,204	wzxE	O‑antigen translocase	P302S (CCG→TCG)	C	T	T	T	T	T
3,981,100	aslB	predicted regulator of arylsulfatase activity	K40N (AAG→AAT)	G			T		
3,984,336	aslA/hemY	acrylsulfatase‑like enzyme/predicted protoheme IX synthesis protein	intergenic (‑306/+373)	G	T	T	T	T	T
4,000,496	yigF	predicted inner membrane protein	T109T (ACG→ACA)	C	T	T	T	T	T
4,002,604	yigI	conserved protein, 4HBT family of thioesterases	F39L (TTC→TTA)	G			T		
4,013,102	metE	5‑methyltetrahydropteroyltriglutamate‑homocysteine S‑methyltransferase	S676L (TCG→TTG)	C	T	T	T	T	T
4,017,853	yigP	conserved protein, SCP2 family	C69C (TGC→TGT)	C	T	T	T	T	T
4,020,728	tatB	TatABCE protein translocation system subunit	P163H (CCT→CAT)	C			A		
4,022,509	rfaH	DNA‑binding transcriptional antiterminator	P112P (CCG→CCT)	C			A		
4,051,447	hemN/yshB	coproporphyrinogen III oxidase, SAM and NAD(P)H dependent, oxygen‑independent/expressed protein	intergenic (+6/+223)	G			T		
4,061,916	ompL	outer membrane porin L	D135N (GAT→AAT)	C	T	T	T	T	T
4,063,020	yihO	predicted transporter	A257E (GCG→GAG)	G			T		

4,063,060	yihO	predicted transporter	A244S (GCT→TCT)	C			A		
4,064,757	yihP	predicted transporter	R154L (CGC→CTC)	C			A		
4,066,347	yihQ	alpha‑glucosidase	S318* (TCA→TAA)	G			T		
4,071,328	yihU	gamma‑hydroxybutyrate dehydrogenase, NADH‑dependent	A89A (GCG→GCT)	C			A		
4,076,440	yiiD	predicted acetyltransferase	E323D (GAG→GAT)	G			T		
4,078,844	fdhE	formate dehydrogenase formation protein	E136D (GAG→GAT)	C				A	A
4,079,366	fdoI	formate dehydrogenase‑O, cytochrome b556 subunit	W173L (TGG→TTG)	C			A		
4,080,005	fdoH	formate dehydrogenase‑O, Fe‑S subunit	L260I (CTC→ATC)	G				T	T
4,080,348	fdoH	formate dehydrogenase‑O, Fe‑S subunit	F145F (TTC→TTT)	G	A	A	A	A	A
4,088,069	frvX	predicted peptidase	G294W (GGG→TGG)	C			A		
4,091,055	frvA/rhaM	predicted enzyme IIA component of PTS/L‑rhamnose mutarotase	intergenic (‑209/+92)	G	A	A	A	A	A
4,095,569	rhaB/rhaS	rhamnulokinase/DNA‑binding transcriptional activator for rhaBAD and rhaT, L‑rhamnose‑binding	intergenic (‑98/‑190)	G		T			
4,102,284	cpxA	sensory histidine kinase in two‑component regulatory system with CpxR	Q239K (CAG→AAG)	G			T		
4,104,023	cpxP	inhibitor of the cpx response; periplasmic adaptor protein	D61Y (GAT→TAT)	G			T		
4,114,550	glpK	glycerol kinase	G232G (GGC→GGA)	G				T	T
4,118,840	hslU	molecular chaperone and ATPase component of HslUV protease	Q311K (CAG→AAG)	G			T		
4,121,607	cytR	DNA‑binding transcriptional dual regulator	P291P (CCG→CCT)	C				A	A
4,132,508	katG	catalase‑peroxidase HPI, heme b‑containing	L217L (CTG→CTT)	G			T		
4,143,556	pflD	predicted formate acetyltransferase 2 (pyruvate formate lyase II)	G513G (GGC→GGA)	C			A		
4,151,450	ppc/argE	phosphoenolpyruvate carboxylase/acetylornithine deacetylase	intergenic (‑329/+269)	G			T		
4,153,719	argC	N‑acetyl‑gamma‑glutamylphosphate reductase, NAD(P)‑binding	L232L (CTG→CTT)	G			T		
4,158,020	sthA	pyridine nucleotide transhydrogenase, soluble	L265Q (CTG→CAG)	A	T	T	T	T	T
4,158,102	sthA	pyridine nucleotide transhydrogenase, soluble	E238* (GAA→TAA)	C				A	A
4,160,648	trmA	tRNA m(5)U54 methyltransferase, SAM‑dependen	E216* (GAG→TAG)	C		A			
4,161,773	btuB	vitamin B12/cobalamin outer membrane transporter	P38T (CCG→ACG)	C	A	A	A	A	A
4,169,780	rrfB/murB	5S ribosomal RNA of rrnB operon/UDP‑N‑acetylenolpyruvoylglucosamine reductase, FAD‑binding	intergenic (+1/‑300)	C	T	T	T	T	T
4,172,095	birA/coaA	bifunctional biotin‑[acetylCoA carboxylase] holoenzyme synthetase/ DNA‑binding transcriptional repressor, bio‑5'‑AMP‑binding/pantothenate kinase	intergenic (+25/+4)	C		A		A	A
4,179,798	rpoB	RNA polymerase, beta subunit	I177I (ATC→ATA)	C			A		
4,180,968	rpoB	RNA polymerase, beta subunit	P567P (CCG→CCT)	G				T	T
4,184,950	rpoC	RNA polymerase, beta prime subunit	V526V (GTG→GTT)	G			T		
4,188,253	yjaZ	stationary phase growth adaptation protein	P149S (CCC→TCC)	C	T	T	T	T	T
4,192,019	thiE	thiamin phosphate synthase (thiamin phosphate pyrophosphorylase)	D70A (GAT→GCT)	T	G	G	G	G	G
4,205,112	purH	fused IMP cyclohydrolase/phosphoribosylaminoimidazolecarboxamide formyltransferase	V148V (GTG→GTT)	C			A		
4,216,546	aceA/aceK	isocitrate lyase/isocitrate dehydrogenase kinase/phosphatase	intergenic (+110/‑73)	C			A		
4,217,416	aceK	isocitrate dehydrogenase kinase/phosphatase	A266A (GCG→GCT)	G				T	T

4,221,661	iclR/metH	DNA‑binding transcriptional repressor/homocysteine‑N5‑methyltetrahydrofolate transmethylase, B12‑dependent	intergenic (‑10/‑190)	T	C	C	C	C	C
4,241,005	malG	maltose transporter subunit	G179C (GGC→TGC)	C			A		
4,241,034	malG	maltose transporter subunit	G169V (GGG→GTG)	C			A		
4,245,697	malK	fused maltose transport subunit, ATP‑binding component of ABC superfamily/regulatory protein	D297E (GAC→GAA)	C			A		
4,249,025	yjbI	pseudogene, pentapeptide repeat‑related	pseudogene (1282/1323 nt)	G	T	T	T	T	T
4,249,451	yjbI	pseudogene, pentapeptide repeat‑related	pseudogene (856/1323 nt)	G			T		
4,250,729	ubiC	chorismate‑‑pyruvate lyase	L67L (CTG→CTT)	G			T		
4,253,901	plsB	glycerol‑3‑phosphate O‑acyltransferase	H197N (CAC→AAC)	G			T		
4,256,680	dinF	DNA‑damage‑inducible SOS response protein	A306S (GCC→TCC)	G		T			
4,258,676	yjbM	predicted protein	D19Y (GAC→TAC)	G				T	T
4,265,314	tyrB	tyrosine aminotransferase, tyrosine‑repressible, PLP‑dependent	P60S (CCT→TCT)	C	T	T	T	T	T
4,267,856	aphA	acid phosphatase/phosphotransferase, class B, non‑specific	S140S (AGC→AGT)	C	T	T	T	T	T
4,274,236	yjcC	predicted cyclic‑di‑GMP phosphodiesterase	P248L (CCC→CTC)	C	T	T	T	T	T
4,278,107	yjcE	predicted cation/proton antiporter	I35I (ATC→ATA)	C		A			
4,285,430	acs/nrfA	acetyl‑CoA synthetase/nitrite reductase, formate‑dependent, cytochrome	intergenic (‑36/‑356)			IS30 (–) +2 bp			
4,285,487	acs/nrfA	acetyl‑CoA synthetase/nitrite reductase, formate‑dependent, cytochrome	intergenic (‑93/‑300)	T	A	A	A	A	A
4,285,831	nrfA	nitrite reductase, formate‑dependent, cytochrome	L15F (TTG→TTT)	G			T		
4,290,833	nrfE	heme lyase (NrfEFG) for insertion of heme into c552, subunit NrfE	P433P (CCG→CCT)	G				T	T
4,291,956	nrfG	heme lyase (NrfEFG) for insertion of heme into c552, subunit NrfG	A131S (GCA→TCA)	G				T	T
4,293,560	gltP	glutamate/aspartate:proton symporter	G353W (GGG→TGG)	G			T		
4,296,358	fdhF	formate dehydrogenase‑H, selenopolypeptide subunit	M344I (ATG→ATT)	C			A		
4,298,919	mdtP	outer membrane factor of efflux pump	A45A (GCC→GCA)	G				T	T
4,323,667	yjdN	conserved protein	S33N (AGC→AAC)	C	T	T	T	T	T
4,333,901	adiC	arginine:agmatin	P385Q (CCG→CAG)	G				T	T
4,339,300	melR	DNA‑binding transcriptional dual regulator	H118N (CAC→AAC)	G			T		
4,348,818	dcuS	sensory histidine kinase in two‑component regulatory system with DcuR, regulator of anaerobic fumarate respiration	R290S (CGC→AGC)	G			T		
4,357,644	cadB	predicted lysine/cadaverine transporter	M137I (ATG→ATT)	C				A	A
4,358,231	cadB/cadC	predicted lysine/cadaverine transporter/DNA‑binding transcriptional activator	intergenic (‑177/+188)	C			A		
4,358,397	cadB/cadC	predicted lysine/cadaverine transporter/DNA‑binding transcriptional activator	intergenic (‑343/+22)	G			T		
4,358,577	cadC	DNA‑binding transcriptional activator	E461K (GAA→AAA)	C	T	T	T	T	T
4,362,486	dsbD	fused thiol:disulfide interchange protein: activator of DsbC/conserved protein	V194L (GTG→TTG)	C				A	A
4,388,045	psd	phosphatidylserine decarboxylase	S113R (AGC→AGA)	G			T		
4,398,466	hfq	HF‑I, host factor for RNA phage Q beta replication	Q52H (CAG→CAT)	G			T		
4,401,886	hflC	modulator for HflB protease specific for phage lambda cII repressor	G188G (GGC→GGA)	C			A		
4,411,621	yjfC	glutathionylspermidine synthase homolog	Q191K (CAG→AAG)	C			A		
4,414,324	yjfN/bsmA	predicted protein/confers peroxide resistance in bioflms	intergenic (‑9/+140)	C			A		

4,414,652	bsmA	confers peroxide resistance in bioflms	D48Y (GAC→TAC)	C				A	A
4,427,507	fklB	FKBP-type peptidyl-prolyl cis-trans isomerase (rotamase)	R184S (CGC→AGC)	C		A			
4,429,523	ytfE	iron-sulfur cluster repair protein RIC	E162* (GAA→TAA)	C				A	A
4,434,245	cpdB	2':3'-cyclic-nucleotide 2'-phosphodiesterase	G115V (GGC→GTC)	C				A	A
4,441,818	ytfM	predicted outer membrane protein and surface antigen	G472S (GGC→AGC)	G	A	A	A	A	A
4,442,555	ytfN	large conserved protein, DUF490 family	D141Y (GAC→TAC)	G			T		
4,442,998	ytfN	large conserved protein, DUF490 family	Q288H (CAG→CAT)	G			T		
4,446,613	chpS	antitoxin of the ChpBS toxin-antitoxin system	R48R (AGG→AGA)	G	A	A	A	A	A
4,449,762	ytfR	predicted sugar transporter subunit: ATP-binding component of ABC superfamily	E228* (GAA→TAA)	G			T		
4,456,264	pmbA	predicted peptidase required for the maturation and secretion of the antibiotic peptide MccB17	A95S (GCG→TCG)	G			T		
4,463,846	treB	fused trehalose(maltose)-specific PTS enzyme: IIB component/IIC component	P120S (CCC→TCC)	G	A	A	A	A	A
4,465,396	treR/mgtL	DNA-binding transcriptional repressor/regulatory leader peptide for mgtA	intergenic (-127/-58)	G			T		
4,468,308	mgtA	magnesium transporter	V887V (GTG→GTT)	G			T		
4,471,923	yjgI	predicted oxidoreductase with NAD(P)-binding Rossmann-fold domain	G52R (GGA→AGA)	C	T	T	T	T	T
4,473,516	yjgL	predicted protein	F19F (TTT→TTC)	T	C	C	C	C	C
4,484,447	lptF	lipopolysaccharide export ABC permease of the LptBFGC export complex	F69L (TTC→TTA)	C				A	A
4,485,830	lptG	lipopolysaccharide export ABC permease of the LptBFGC export complex	E164* (GAG→TAG)	G			T		
4,485,834	lptG	lipopolysaccharide export ABC permease of the LptBFGC export complex	R165L (CGG→CTG)	G			T		
4,497,555	insD/yjgX	IS2 transposase TnpB/KpLE2 phage-like element; predicted protein, C-ter fragment (pseudogene)	intergenic (+32/+61)	G			T		
4,507,841	yjhV	pseudogene, KpLE2 phage-like element	pseudogene (15/330 nt)	C				A	A
4,508,261	yjhV/fecE	pseudogene, KpLE2 phage-like element/KpLE2 phage-like element; iron-dicitrate transporter subunit	intergenic (+105/+452)	T	G	G	G	G	G
4,511,900	fecB	KpLE2 phage-like element; iron-dicitrate transporter subunit	G144G (GGC→GGA)	G			T		
4,511,916	fecB	KpLE2 phage-like element; iron-dicitrate transporter subunit	S139F (TCT→TTT)	G	A	A	A	A	A
4,515,890	fecI	KpLE2 phage-like element; RNA polymerase, sigma 19 factor	G123G (GGC→GGA)	G			T		
4,524,078	yjhI/sgcR	KpLE2 phage-like element; predicted DNA-binding transcriptional regulator/KpLE2 phage-like element; predicted DNA-binding transcriptional regulator	intergenic (-252/+51)	G			T		
4,536,291	nanM	N-acetylneuraminic acid mutarotase	T166T (ACC→ACA)	G			T		
4,536,414	nanM	N-acetylneuraminic acid mutarotase	P125P (CCG→CCT)	C			A		
4,538,315	nanC/fimB	N-acetylnuraminic acid outer membrane channel protein/tyrosine recombinase/inversion of on/off regulator of fimA	intergenic (-791/-665)	G	A	A	A	A	A
4,540,065	fimE	tyrosine recombinase/inversion of on/off regulator of fimA	coding (6-13/597 nt)		IS1 (+) +8 bp	IS1 (+) +8 bp	IS1 (+) +8 bp	IS1 (+) +8 bp	IS1 (+) +8 bp
4,540,749	fimE/fimA	tyrosine recombinase/inversion of on/off regulator of fimA/major type 1 subunit fimbrin (pilin)	intergenic (+93/-389)	C				A	A
4,543,108	fimC/fimD	chaperone, periplasmic/outer membrane usher protein, type 1 fimbrial synthesis	intergenic (+56/-11)	C				A	A
4,546,338	fimG	minor component of type 1 fimbriae	A11T (GCG→ACG)	G	A	A	A	A	A

4,546,928	fimH	minor component of type 1 fimbriae	P33H (CCT→CAT)	C	A	A	A	A	A
4,547,014	fimH	minor component of type 1 fimbriae	Q62K (CAA→AAA)	C				A	A
4,555,786	qseD	Cell density-dependent motility repressor	P176H (CCT→CAT)	G	T	T	T	T	T
4,561,801	yjiK/yjiL	conserved protein/predicted ATPase, activator of (R)-hydroxyglutaryl-CoA	intergenic (-175/+144)		Δ1 bp	Δ1 bp	Δ1 bp	Δ1 bp	Δ1 bp
4,562,397	yjiL	predicted ATPase, activator of (R)-hydroxyglutaryl-CoA dehydratase	Q106K (CAG→AAG)	G		T			
4,573,252	yjiV	pseudogene; conserved hypothetical protein	pseudogene (1311/2937 nt)	G			T		
4,576,088	mcrB	5-methylcytosine-specific restriction enzyme McrBC, subunit McrB	L425F (CTT→TTT)	G	A	A	A	A	A
4,577,111	mcrB	5-methylcytosine-specific restriction enzyme McrBC, subunit McrB	D84Y (GAC→TAC)	C			A		
4,579,064	hsdS	specificity determinant for hsdM and hsdR	G141D (GGT→GAT)	C	T	T	T	T	T
4,592,308	yjjL	L-galactonate transporter	P146P (CCG→CCT)	C			A		
4,603,196	fhuF	ferric iron reductase involved in ferric hydroximate transport	A164E (GCG→GAG)	G			T		
4,607,458	prfC	peptide chain release factor RF-3	Q8K (CAA→AAA)	C			A		
4,614,692	yjjI	conserved protein	L133I (CTA→ATA)	G	T	T	T	T	T
4,619,149	deoD	purine-nucleoside phosphorylase	G82C (GGC→TGC)	G			T		
4,623,518	serB	3-phosphoserine phosphatase	A201S (GCG→TCG)	G	T	T	T	T	T
4,631,607	yjjX	inosine/xanthosine triphosphatase	G54G (GGC→GGA)	G		T			
4,637,525	creD	inner membrane protein	T442M (ACG→ATG)	C	T	T	T	T	T
4,637,533	creD	inner membrane protein	E445Q (GAG→CAG)	G	C	C	C	C	C
4,637,535	creD	inner membrane protein	E445E (GAG→GAA)	G	A	A	A	A	A
4,637,631	arcA	DNA-binding response regulator in two-component regulatory system with ArcB or CpxA	C233C (TGC→TGT)	G	A	A	A	A	A
4,637,655	arcA	DNA-binding response regulator in two-component regulatory system with ArcB or CpxA	I225I (ATT→ATC)	A	G	G	G	G	G
4,637,742	arcA	DNA-binding response regulator in two-component regulatory system with ArcB or CpxA	P196P (CCG→CCA)	C	T	T	T	T	T
4,637,787	arcA	DNA-binding response regulator in two-component regulatory system with ArcB or CpxA	S181S (TCC→TCT)	G	A	A	A	A	A
4,637,979	arcA	DNA-binding response regulator in two-component regulatory system with ArcB or CpxA	L117L (CTA→CTG)	T	C	C	C	C	C
4,638,808	yjjY/yjtD	predicted protein/predicted rRNA methyltransferase	intergenic (+243/-157)	C	T	T	T	T	T
4,638,887	yjjY/yjtD	predicted protein/predicted rRNA methyltransferase	intergenic (+322/-78)	A	G	G	G	G	G
4,638,931	yjjY/yjtD	predicted protein/predicted rRNA methyltransferase	intergenic (+366/-34)	G	T	T	T	T	T
4,638,947	yjjY/yjtD	predicted protein/predicted rRNA methyltransferase	intergenic (+382/-18)	A	G	G	G	G	G
4,639,045	yjtD	predicted rRNA methyltransferase	G27G (GGG→GGT)	G	T	T	T	T	T
4,639,054	yjtD	predicted rRNA methyltransferase	D30E (GAT→GAA)	T	A	A	A	A	A
4,639,154	yjtD	predicted rRNA methyltransferase	L64L (TTG→CTG)	T	C	C	C	C	C
4,639,231	yjtD	predicted rRNA methyltransferase	A89A (GCC→GCT)	C	T	T	T	T	T
4,639,240	yjtD	predicted rRNA methyltransferase	V92V (GTT→GTA)	T	A	A	A	A	A
4,639,249	yjtD	predicted rRNA methyltransferase	V95V (GTG→GTA)	G	A	A	A	A	A
4,639,319	yjtD	predicted rRNA methyltransferase	L119L (TTG→CTG)	T	C	C	C	C	C

4,639,375	yjtD	predicted rRNA methyltransferase	A137A (GCG→GCA)	G	A	A	A	A	A
4,639,405	yjtD	predicted rRNA methyltransferase	A147A (GCG→GCA)	G	A	A	A	A	A
4,639,451	yjtD	predicted rRNA methyltransferase	A163T (GCG→ACG)	G	A	A	A	A	A
4,639,462	yjtD	predicted rRNA methyltransferase	coding (498-499/687 nt)		2 bp-->CA	2 bp-->CA	2 bp-->CA	2 bp-->CA	2 bp-->CA
4,639,506	yjtD	predicted rRNA methyltransferase	A181V (GCC→GTC)	C	T	T	T	T	T
4,639,511	yjtD	predicted rRNA methyltransferase	T183A (ACA→GCA)	A	G	G	G	G	G
4,639,567	yjtD	predicted rRNA methyltransferase	Q201Q (CAA→CAG)	A	G	G	G	G	G
