# Supplementary material for: Ex Uno Plures: Clonal Reinforcement Drives Evolution of a Simple Microbial Community
Source: PLoS Genet. 2014 Jun 26;10(6):e1004430. doi: 10.1371/journal.pgen.1004430 (PMC4072538; doi:10.1371/journal.pgen.1004430)
Supplement: Table S3 — Intracellular pyruvate. (PDF) [file pgen.1004430.s003.pdf]

Table S3. Intracellular pyruvate

Strain	Protein, mg/ml	SD	[PYR] nmol/mg	SD
JA122	1.16	0.09	5.61	0.34
101	1.28	0.10	1.68	0.10
103	1.36	0.11	1.61	0.10
116	1.15	0.09	2.22	0.13
community	0.94	0.07	1.95	0.12
