# Supplementary material for: Ex Uno Plures: Clonal Reinforcement Drives Evolution of a Simple Microbial Community
Source: PLoS Genet. 2014 Jun 26;10(6):e1004430. doi: 10.1371/journal.pgen.1004430 (PMC4072538; doi:10.1371/journal.pgen.1004430)
Supplement: Table S4 — Mutations associated with formate metabolism. (PDF) [file pgen.1004430.s004.pdf]

Table S4. Mutations associated with formate metabolism

Position	SNP	aa change (codon change)	Gene name	Function	Strain affected
860,425	C→A	A469S (GCG→TCG)	ybiW	predicted pyruvate formate lyase	103
949,689	C→A	L205L (CTG→CTT)	pflA	pyruvate formate lyase activating enzyme 1	103
4,143,556	C→A	G513G (GGC→GGA)	pflD	predicted formate acetyltransferase 2 (pyruvate formate lyase II)	103
953,136	C→A	G185V (GGC→GTC)	focA	formate channel	103
2,612,001	G→T	A16S (GCG→TCG)	focB	predicted formate transporter	103
1,547,701	C→A	S759S (TCC→TCA)	fdnG	formate dehydrogenase-N, alpha subunit, nitrate-inducible	115/116
deleted	N/A	N/A	fdnG	formate dehydrogenase-N, α subunit	103
deleted	N/A	N/A	fdnH	formate dehydrogenase-N, (β) subunit	103
deleted	N/A	N/A	fdnI	formate dehydrogenase-N, (γ) subunit	103
4,078,844	C→A	E136D (GAG→GAT)	fdhE	formate dehydrogenase formation protein	115/116
4,296,358	C→A	M344I (ATG→ATT)	fdhF	formate dehydrogenase-H, selenopolypeptide subunit	103
4,079,366	C→A	W173L (TGG→TTG)	fdoI	formate dehydrogenase-O, cytochrome b556 subunit	103
4,080,005	G→T	L260I (CTC→ATC)	fdoH	formate dehydrogenase-O, Fe-S subunit	115/116
4,080,348	G→A	F145F (TTC→TTT)	fdoH	formate dehydrogenase-O, Fe-S subunit	JA122
