# Supplementary material for: Ex Uno Plures: Clonal Reinforcement Drives Evolution of a Simple Microbial Community
Source: PLoS Genet. 2014 Jun 26;10(6):e1004430. doi: 10.1371/journal.pgen.1004430 (PMC4072538; doi:10.1371/journal.pgen.1004430)
Supplement: Table S5 — SNP verification primers. (PDF) [file pgen.1004430.s005.pdf]

Table S5. SNP verification primers

Name	Sequence (5' →3')
aceA/aceK F	TTGACCTGGCAAACGCCTAT
aceA/aceK R	CGACCAGACCAACGTGATGA
cpxAF	AACTGTCAACGACTTGCCCA
cpxAR	CGCGTAAGCTGAAAAACGCT
cpxPF	TGATGCGCATAGTTACCGCT
cpxPR	GTCACGCAACTGCTCCATTC
enoF	AGCCAGAGATACAGCCAGGA
enoR	GCTGTTTGCAGGCTTTGTGA
fdoIF	TATCCGCCGTACGTTTCTCG
fdoIR	TGGGCGGCGATTATTTTCCT
fhlAF	TCGATTTACCACACGCCGAA
fhlAR	ACTCTTTTTCGCTCCAGGGG
frmAF	GTTGTGTACCGCGCCAATAC
frmAR	AAATTGACGTTGCACCACCG
gltAF	GCAATTTCACGGTGACGAGG
gltAR	TCACCTTTGACCCAGGCTTC
gpsAF	GAAAAGGCACATCGGGGAGA
gpsAR	TTTCTCCATCGCGGGTATCG
iclR/metH F	TGCCGACCATAAAGGCATGT
iclR/metH R	ACGCAGTTGTTCCACTTTGC
lipB F	AACGGAACGCTTTTGCTCAC
lipB R	ATTCACCCAAGGTCAGGCAG
lpdF	TTCTGGGTGCAGCAAGGTAG
lpdR	GCAGGCGTTCTGGTACTTCT
maeAF	ACCATACCTTCGCCGTTCAG
maeAR	GCTTTGGCTTGCTGACTGAC
malGF	TTGCGGGTTGAGGTATTGCT
malGR	GTGGCTGTGGAACTCGGTAA
malKF	CGCAATCGATCAAGTGCAGG
malKR	CTTAAACGCCCGGCTCCTTA
malPF	TTACGATAGCCGACAACGGG
malPR	ACCTGTTGAATCTCGGCTGG
malTF	GGTTCGACTCGACCATACCG
malTR	GAAGGTTGCGTGACAACACC
mutMF	GTCCAAGATGGGTCAGCACA
mutMR	TTCGATCCAGTTGTTCGCCA
mutY F	GAAATGTTCGCTCTGTCCGC
mutY R	GCCCAGGCTTCTTTGGAGAT
narPF	GATGCCAAAGCGAACGACAA

narPR	TGACGCGTTCGCTAAAGACT
ompR/greB F	ACGCGGGTTAAACGGTTTTG
ompR/greB R	AGGGGCGTTTTCATCTCGTT
poxBF	GCGGAATAGCTCTTGTGGGT
poxBR	AAGGTTACGTACTGGCCTGC
ptsIF	TGCGCTAACATTGGTACGGT
ptsIR	TAAATTGGGCCGCATCTCGT
rhoF	GATTTGGTTTCCTCCGTTCCG
rhoR	CTGCAGTTCTTCCTGAGTCGT
rpoD F	AACGGCTTAAGTGCCGAAGA
rpoD R	GTGCGCCCGATTTCAGATTC
rpoS F	AACGGCGGGCAATTTTTACC
rpoS R	TGTTCCGTCAAGGGATCACG
sdhB/sucA F	CAGAGCAGCGCGAAAAACTC
sdhB/sucA R	TTGTCTACGTAGTGGCTCGC
yfiQF	CTTTCCCGGCTTTCTGCCTA
yfiQR	GATCTTCGTGTTGCGATGCC
glpKF	CGGAATCGTAGGCGTCGTTA
glpKR	TAACGCCATTGTCTGGCAGT
gutQF	GATGATCGAAAGCCGCGATG
gutQR	AGCGTTCCAGCGTGAAACTA
fdnGF	GCTGAGTAGCTTTGCGCATC
fdnGR	TTGTTGATGGCAAACAGGCG
glpRF	AATCAAGGCGGAACTGGGAG
glpRR	GATGGGCTACGTCTGGCTAC
